# An equivalence approach to the integrative analysis of feature lists

**DOI:** 10.1186/s12859-019-3008-x

**Published:** 2019-08-27

**Authors:** Alex Sánchez-Pla, Miquel Salicrú, Jordi Ocaña

**Affiliations:** Genetics, Microbiology and Statistics Department, Universitat de Barcelona, Avinguda Diagonal, 648, Barcelona, 08028 Spain

**Keywords:** Gene lists, Feature lists, Equivalence tests, Functional profiles

## Abstract

**Background:**

Although a few comparison methods based on the biological meaning of gene lists have been developed, the goProfiles approach is one of the few that are being used for that purpose. It consists of projecting lists of genes into predefined levels of the Gene Ontology, in such a way that a multinomial model can be used for estimation and testing. Of particular interest is the fact that it may be used for proving equivalence (in the sense of “enough similarity”) between two lists, instead of proving differences between them, which seems conceptually better suited to the end goal of establishing similarity among gene lists. An equivalence method has been derived that uses a distance–based approach and the confidence interval inclusion principle. Equivalence is declared if the upper limit of a one-sided confidence interval for the distance between two profiles is below a pre-established equivalence limit.

**Results:**

In this work, this method is extended to establish the equivalence of any number of gene lists. Additionally, an algorithm to obtain the smallest equivalence limit that would allow equivalence between two or more lists to be declared is presented. This algorithm is at the base of an iterative method of graphic visualization to represent the most to least equivalent gene lists. These methods deal adequately with the problem of adjusting for multiple testing. The applicability of these techniques is illustrated in two typical situations: (i) a collection of cancer-related gene lists, suggesting which of them are more reasonable to combine –as claimed by the authors– and (ii) a collection of pathogenesis–based transcript sets, showing which of these are more closely related. The methods developed are available in the goProfiles Bioconductor package.

**Conclusions:**

The method provides a simple yet powerful and statistically well-grounded way to classify a set of genes or other feature lists by establishing their equivalence at a given equivalence threshold. The classification results can be viewed using standard visualization methods. This may be applied to a variety of problems, from deciding whether a series of datasets generating the lists can be combined to the simplification of groups of lists.

**Electronic supplementary material:**

The online version of this article (10.1186/s12859-019-3008-x) contains supplementary material, which is available to authorized users.

## Background

### Gene lists and gene list analysis

Omics technologies are characterized by the fact that the analysis of the data generated often yields what is known as “lists of genes” or, more generally, “lists of features”. Features can be genes, proteins, microRNAs, etc., that may have been selected for taking different values between two or more conditions (that is, for being “differentially expressed”) or for having good capability to discriminate between two or more classes, or to predict the class to which a new individual belongs.

In a simplified way, one can usually consider “feature lists” to represent a kind of summary of what is being analyzed. It is important, however not to forget that this is not exempt from criticism.

- First of all, these lists are often formed by multiple elements associated with each main “feature”, i.e., several transcripts of a gene, several peptides of a protein or multiple methylation sites of a gene. The way in which multiple features collapse into a single one in order to be summarized (the average, the most variable, etc.) is not exempt from arbitrariness.
- Secondly, and even more importantly, the lists are usually obtained by applying a cut–off value with a certain statistical basis (for example, an adjusted p–value ≤0.05), which means that this list *may* include (or exclude) genes that a reasonable change in the selection criteria might exclude (or include).

Although much has been discussed about these issues, and alternative approaches have been sought, the use of a list as a summary of an experiment is still a very common approach. This is not without foundation from a statistical point of view, where it is generally assumed that a summary may contain less information than all data.

#### Analysis of individual feature lists

The analysis of gene lists has a long history, probably as long as the analysis of genomic data. Draghici [1] introduced enrichment analysis or over–representation analysis, which selects annotations that appear with a surprisingly (unexpected) high frequency if we take into account how they are distributed among all genes. Mootha [2, 3] introduced the gene set enrichment analysis method as an alternative to the analysis of cutoff–based lists. This method analyzes all data (instead of the list) looking for annotations that tend to appear in extreme cases (between genes up- or downregulated) without requiring the list to be cut by a point. Shojaie [4] went one step further and performed the analysis of the lists based on the regulatory network implicitly associated with them. These three methods are nothing more than the first of literally dozens of variants of the same ones that have been developed in the last decade. Khatri [5] is an excellent review of this process. Essentially all these methods share one characteristic i.e. they are focused on the analysis of single feature lists. Despite their relevance, which is why we are mentioning them, their purpose is different from what is discussed in this work: They do not seek to compare lists but rather extract the biological information and therefore will not be discussed here.

#### Comparison between two or more feature lists

Comparison between lists has a shorter history because, curiously, it is a topic that has attracted less attention than the analysis of individual lists. This is probably for the same reasons that there has been a tendency to perform individual studies rather than to compare or group them: the cost of studies, especially in their initial stage and often their low reproducibility. In addition, many methods or tools for comparing gene lists are based on a well–defined statistical model, which also suggests that this has been a relatively marginal issue. In general, we can differentiate between: (i) methods that compare the composition of the lists, either simply by the identifiers that form them or by the ranges of those in the list; and (ii) methods that project the elements in some other space such as the Gene Ontology or provide other representations of the list such as co–expression networks.

Among the first approaches, we find programs such as GeneVenn [6], BioVenn [7] and VennPainters [8] that perform a visual comparison based on more or less flexible forms of Venn diagrams and are therefore limited in terms of the number of lists that may be compared. There are also applications such as CORal [9], Rank–Rank Hypergeometric Overlap [10] and OrderedLists [11] that are based on determining the degree of overlap of two or more ordered lists. One characteristic of all these methods is that if they perform the comparison visually or by using a statistical model, they do not refer to the biological meaning of the list elements.

In this work, we are interested in methods that, in some way, rely on the biological information in the lists. This is usually done by basing the comparison on the annotations of the genes in biological knowledge databases, such as the Gene Ontology [12, 13]. There are several distinct approaches to doing this. Some programs start by conducting an overrepresentation (or gene enrichment) analysis of each list and then the set of enriched GO terms obtained from each list is compared. This is the case, for instance, with the PANTHER web tool [14]. The clusterProfiler Bioconductor package [15] can also be used in a very flexible manner, enabling comparison of two or more gene lists based on the corresponding GO or KEGG enriched terms. A different approach is to rely on semantic similarity measures [16], which are used to compare GO terms or entities annotated with GO terms (in this case gene lists), by leveraging on the ontology structure and properties. The GOSemSim Bioconductor package [17] enables computation of a variety of such measures, which may be used to compare gene lists. Last, the method extended in this paper was introduced in [18, 19] with the aim of providing an inferential basis for comparing two gene lists. It is based on the annotations of the lists at a fixed level of the Gene Ontology. The method is implemented in the goProfiles package [20] also available from Bioconductor.

#### Comparison between multiple lists

As omics studies have become more complex, we are faced with the need and the opportunity to work with several, or even many, gene lists. We may have distinct scenarios for this. For example:

- Sometimes researchers want to obtain “as much as possible” from their expensive omics experiments and compare *everything vs everything* even if some comparisons are not relevant. This may result in dozens of gene lists, which may contain redundant information.
- Sometimes researchers collect gene lists from different studies because they consider these to be about the same biological problem. This is the case with the examples that will be discussed later in the paper:
  - Cancer-related gene lists (http://www.bushmanlab.org/links/genelists, [21])
  - Pathogenesis-based transcript sets (https://www.ualberta.ca/medicine/institutes-centresgroups/atagc/research/gene-lists, [22])
- The Molecular Signature Database (MsigDB, [23]) contains thousands of gene lists that might benefit from some type of dimension reduction.

One may want to work on these lists for different purposes which we may classify simplistically as *dimension reduction* or *dimension augmentation*. (i) Dimension reduction here would mean trying to reduce the number of lists: for instance, referring to the previous example, having a smaller number of relevant signatures, or simplifying the exploration of lists that result from some omics experiments. (ii) Dimension augmentation, on the other hand, may mean the possibility of combining datasets associated with gene lists derived from them. In other words, if one can establish that a few lists are equivalent, one may assume that the data that have generated them can also be considered equivalent, or, which is the same thing, can be combined into a single bigger dataset.

In recent years there have appeared several programs intended to allow the comparison of more than two lists. listcompare is a web tool that checks overlap of multiple gene lists (http://www.molbiotools.com/listcompare.html) without making any use of biological information contained in the lists. Other tools, such as clusterProfiler [15] or ToppCluster [24], perform a comparison based on doing an enrichment analysis of each gene list and then relying on the enriched categories to compare the lists. This comparison is made either descriptively (clusterProfiler) or interactively building a network with the enriched terms (ToppCluster). The former tools, especially clusterProfiler, have the merit that their comparison provides hints on the biological difference between the lists because they are based on enriched GO categories. A drawback, however, is the fact that these comparisons are visual only, with no inferential basis behind them. The method presented in this paper does not directly highlight the categories explaining the differences between the lists but does provide an inferential basis for the comparison, somehow complementing the others.

### Difference vs equivalence hypotheses tests

A common misconception among practitioners of statistics is to take up the fact of not rejecting a null hypothesis as a proof of its veracity. The phrase “to accept the null hypothesis”, though very common, is a statistical nonsense. If anything (to some extent) is proven in an hypotheses test, it is the alternative hypothesis when the null hypothesis is rejected, but no inferences can be drawn from not rejecting it. Posterior power arguments, say that to try to infer the veracity of a nonrejected null hypothesis from arguments of its (high) power computed from the parameter estimates may lead to paradoxes [25]. Thus, if the objective of an study is to prove similarity, e.g. to decide if the biological information provided by two gene lists is similar (but not necessarily exactly equal), from an hypotheses testing perspective the right approach would be to contrast a null hypothesis of relevant dissimilarity against an alternative of irrelevant dissimilarity (not necessarily null dissimilarity, i.e., exact equality, which may lead to an undemonstrable statement). In practice, this may be implemented by choosing a measure of dissimilarity and establishing a threshold *Δ* of “acceptable” dissimilarity. Then, the null hypothesis should specify that the true measure of dissimilarity is not less than *Δ* while, conversely, the alternative should specify that the dissimilarity is less than *Δ*.

### Objectives

In this paper we present a statistical approach that allows for: (i) given a predetermined equivalence threshold *Δ*, to check the biological equivalence of the set of lists; and (ii) for a set of given lists, to determine the minimum equivalence threshold that allows the equivalence of the set to be declared. With this approach, the efficiency of the statistical tests is evaluated in a simulation study and a graphic representation is provided to facilitate the interpretation of the results. The application is illustrated using two publicly available sets of gene lists (Kidney Gene Lists and Cancer Gene Lists). The goProfiles Bioconductor package has been extended to include the new capabilities of the method.

## Results

### Previous work: statistical inference for functional profiles

The methods proposed in this work rely on biological knowledge to compare two or more gene lists. In practice, this means that biological annotations are used to characterize each list in such a way that a method for comparing these characterizations can be used. The method, known as *goProfiles*, has been introduced elsewhere ([18, 19]) and is reviewed briefly below.

Given a list of *n* features annotated in the Gene Ontology (GO), a reasonable way to characterize this list is to count how many features are annotated in each category (see Figure 2 in [18]). This yields a frequency table that we call *functional profile* describing how these *n* features are distributed between *C*_1_,*C*_2_,…,*C*_*s*_ GO categories. However, given that a feature can be annotated in more than one category, the frequencies obtained may add up to more than *n* when counts are considered, or to more than 1 if we rely on proportions. This complicates the analysis because, for example, a chi–squared approach cannot be used to model or compare these frequency tabulations. This was solved in [18] by introducing the ideas of “expanded” and “contracted” profiles. It is very common for a given feature to be annotated in several categories. An expanded profile allows multiple annotations to be transformed into simple ones so that each feature is annotated in one and only one category. This is possible by defining this profile on the Cartesian product partition $C, C\times C, \dots, \underbrace {C\times \dots \times C}_{s}$ excluding symmetric products. With this formulation, the *expanded profile* is the vector of probabilities

1 $$ \mathcal {P}=(p_{1},\dots, p_{s},p_{11},...,p_{(s-1)s},\dots, p_{123...s})  $$

where each $p_{i_{1}, i_{2},...,i_{k}},\, k\leq s$, describes the probability of simultaneous annotation in a possible combination of categories so that a feature will always be annotated in one and only one category of such expanded profiles. Alternatively, the *contracted profile*, or simply *profile* for short, is the vector of probabilities defined on the original categories.

2 $$ P=\left(p_{1.},\dots, p_{s.}\right)  $$

where *p*_*i*._ describes the probability of annotation in category *C*_*i*_, *i*=1,...,*s*. Expanded and contracted profiles are related by a simple linear transformation (a “contraction”) that turns an expanded into a contracted profile.

Given two lists of features of size *n* and *m* respectively, the dissimilarity between their associated functional profiles *P*, *Q* can be evaluated by the squared Euclidean distance (which in fact is not a true metric distance, just a dissimilarity, as it does not verify the triangular inequality) between them:

$$d\left({P,\,\, Q} \right) = \sum\limits_{i = 1}^{s} {\left({ p_{i\cdot} - q_{i\cdot}} \right)^{2} }. $$

All quantities can be naturally estimated by their relative frequencies. Salicrú et al. [19] obtained the asymptotic distribution of the estimated distance between two profiles and relied on this result to derive hypothesis tests for comparing two feature lists.

3 $$ {\begin{aligned} \left({\frac{{n{\mathrm{ }}m}}{{n + m}}} \right)^{1/2} d(\hat P \,-\, P,\hat Q \,-\, Q) \xrightarrow{d} Y \!\sim\! N\left(0,{\mathrm{ }}\sigma_{{\mathcal P}{\mathcal Q}} \right) \approx N\left(0,{\mathrm{ }}\sigma_{\hat {\mathcal P}\hat {\mathcal Q}} \right),  \end{aligned}}  $$

where the “hat” notation stands for the sample profiles (the unknown probabilities substituted by the corresponding relative frequencies) and the general expression of $\sigma _{{\mathcal P}{\mathcal Q}}$ is given in [19].

Besides classical comparison tests (to establish possible “difference” against a null hypothesis of complete equality), equivalence tests (e.g. [26]) appear to be the natural approach to testing when the goal is establishing (near) equality. In accordance with the global distance-based approach of the present paper, the problem can be stated as follows:

Given two population profiles *P* and *Q*, instead of testing

4 $$  H_{0}: d\left(P,Q\right) = 0\quad vs. \quad H_{1}: d\left(P,Q\right) > 0,  $$

one aims to test:

5 $$ H_{0}: d\left(P,Q\right) \ge \Delta\quad vs. \quad H_{1}: d\left(P,Q\right) < \Delta,  $$

where “near equality” is stated in terms of a “practical equivalence value”, *Δ*>0.

Choosing the threshold *Δ* is a difficult question in equivalence testing –and a subject commonly but wrongly ignored in “difference testing” (4), as a “statistic ally significant” difference (from zero) does not mean “biologically (clinically, etc.) interesting” difference. In general, the chosen *Δ* value should be an expert choice. But as can be seen below, the method finally proposed in the present paper does not depend on a given *Δ* choice.

The so-called “Interval Inclusion Rule” (e.g. as stated in [27] under a distance-based approach) provides a general way of solving equivalence testing problems. It can be stated as follows (again adapted to the distance-based approach):

- Obtain a 1−*α* one-sided confidence interval [0,*d*_*U*_] for *d*(*P, Q*),
- Reject *H*_0_ in (5) if this confidence interval is fully included in the parametric region of *H*_1_, say if *d*_*U*_≤*Δ*.

The above criterion defines a test with significance level *α*.

A consequence of (3) is that

6 $$ d_{U} = d\left(\hat P,\hat Q\right) + z_{1-\alpha} \hat {se}_{\hat d}   $$

is an asymptotically valid upper confidence level, where $\hat {se}_{\hat d} = \sigma _{\hat {\mathcal P}\hat {\mathcal Q}} \sqrt {\frac {1}{n}+\frac {1}{m}}$ stands for the estimated standard error of $d\left (\hat P,\hat Q\right)$ and *z*_1−*α*_ for the 1−*α* quantile of a standard Gaussian distribution, *N*(0,1).

For convenience and in line with further developments in this paper, the criterion to declare equivalence may be restated in terms of p–values *p*(*Δ*) as:

Declare equivalence if:

7 $$ p(\Delta) = \Phi (T_{\hat P\hat Q}(\Delta))= P [Z\leq T_{\hat P \hat Q}(\Delta)] \le \alpha,  $$

where *Φ* stands for the standard normal cumulative distribution function and

8 $$ T_{\hat P \hat Q}(\Delta) = \frac {d(\hat P, \hat Q) - \Delta}{\hat se_{\hat P \hat Q}}.  $$

The above notation tries to highlight that both the p–value and test statistic $T_{\hat P\hat Q}(\Delta)$ depend on the chosen equivalence limit, a crucial matter in the following sections.

### Equivalence test based on functional profiles for *h*≥2 comparisons

Let *L*_1_,...,*L*_*s*_ be *s* distinct lists, e.g. coming from *s* studies on a similar subject. We wish to do a certain number, *h*≤*s*×(*s*−1)/2, of previously specified comparisons (equivalence tests) between these lists. For a given equivalence threshold *Δ*, this can be done by:

- first performing every selected comparison,
- and then doing a multiple testing adjustment in order to deal with testing multiplicity.

There are many approaches to accounting for multiplicity. If the number of comparisons *h* is not big (e.g. at most some tens, note that we are dealing with feature lists comparisons, not with comparing the individual features coming from a given study), a reasonable approach is to control the “family wise error rate” (FWER), for instance, using the Holm–Bonferroni criterion:

- Given an equivalence limit *Δ*, compute the p–values *p*_1_(*Δ*),*p*_2_(*Δ*),…,*p*_*h*_(*Δ*) associated with the test statistics *T*_*l*_(*Δ*)=*T*_*ij*_(*Δ*), *l*=1,2,…,*h*; *i, j*∈{1,…,*s*}.
- Sort the *p*–values in ascending order: *p*_(1)_≤*p*_(2)_≤⋯≤*p*_(*h*)_,
- The null hypothesis of non–equivalence (i.e. existence of a “relevant” functional profile dissimilarity between lists) is rejected for all those comparisons *l*=1,...,*k*−1 such that *p*_*l*_(*Δ*)<*p*_*k*_(*Δ*) where *k* is the smallest value satisfying that *p*_(*k*)_(*Δ*)>*α*/(*h*+1−*k*).

In the case of a great number *h* of comparisons, possibly other criteria like the false discovery rate (FDR) for multiple testing corrections would be the option to choose, but the general idea is still the same.

### Algorithm

As has been stated before, choosing the equivalence limit may be a problematic task. But here we take what would be a complementary approach: instead of previously fixing *Δ*, we will let it vary in order to give a numerical value aiming to measure what would be a statistically significant equivalence between lists, prone to graphical representation and possible interpretation.

The first step is to build a dissimilarity matrix between lists based on the threshold that makes them equivalent:

1. Set *h*=*s*×(*s*−1)/2
2. Let *Δ*_*h*_ be the smallest value allowing the rejection of all *h* null hypotheses, i.e. making *k*=*h*. Then one has:
  $$ \Delta_{h}\,=\,\min_{\Delta \in (0, \infty)}\{\Delta: p_{(l)}(\Delta) \!\leq\! \alpha/(h+1-l), \, l\,=\,1,2,\dots,h\}. $$
3. Obtain *Δ*_*h*_ and take it as the threshold of equivalence distance between lists *i, j* corresponding to the last position in the vector of ordered p–values, i.e. *Δ*_*ij*_=*Δ*_*h*_.
4. Set *h*=*h*−1, exclude comparison between *i* and *j* above and iterate step 2 until *h*=0.

The resulting dissimilarity matrix may be the input for an adequate representation method.

### Visualization

Any data representation method accepting dissimilarity matrices as a starting point can be applied using the *Δ*_*ij*_ matrix. For example, it may be used to construct a dendrogram showing the equivalence levels at which sets of lists may be considered *significantly* equivalent. Although many clustering methods may be used to build the dendrogram, as a first approach, the “maximum distance” or “complete linkage” method seems to be a reasonable and useful choice. The maximum distance method defines the distance between two groups as the distance between their two farthest members. For a given set of lists the construction of dendrograms using this method is in accordance with the declaration of equivalence of the most extreme lists, and consequently with the declaration of equivalence of all pairs of lists.

### Simulation study

An extensive simulation study was performed to investigate the properties of the equivalence test. Several simulation scenarios were created to cover a variety of situations found in practice. These scenarios were considered: (i) the number of common (*n*_0_) and distinct (*n*_1_=*n*−*n*_0_, *m*_1_=*m*−*n*_0_) features in each list (ii) the number of GO nodes on which the profiles are based and (iii) the distribution of annotations along the nodes in both profiles. The simulated scenarios were generated crossing all levels of the factors described above:

- (*n, m*)=(100,100), (200,200), (300,100), (300,300), (400,200), (1000,1000), (1500,500) and *n*_0_ corresponding to 10%, 20% and 50% of *min*(*n*,*m*).
- *s*=10,50,100, where *s* corresponds to the number of simulated GO nodes or items (in other words, the length of the simulated basic profiles).
- *θ*=0.55,0.65,0.75,0.85,0.95 and *θ*_0_=0.5.
- *Δ*=0.025,0.25,1.25.

Each simulation consisted in repeatedly iterating the following steps:

1. Independently generate three expanded profiles $\widehat { \mathcal P}_{1}$, $ \widehat { \mathcal Q}_{1}$ and $\widehat { \mathcal R}$, from a multinomial distribution of sizes *n*_1_, *m*_1_ and *n*_0_ and respective probability parameters:
  $$ {\begin{aligned} \mathcal{P}_{1} &=& \left({{p_{1}},{p_{2}}, \ldots,{p_{s}},{p_{11}}, \ldots,{p_{\left({s - 1} \right)s}}, \ldots,{p_{1 \ldots k}}, \ldots,{p_{\left({s - \left({k - 1} \right)} \right) \ldots \left({s - 1} \right)s}}} \right) \\ \mathcal{Q}_{1} &=& \left({{q_{1}},{q_{2}}, \ldots,{q_{s}},{q_{11}}, \ldots,{q_{\left({s - 1} \right)s}}, \ldots,{q_{1 \ldots k}}, \ldots,{q_{\left({s - \left({k - 1} \right)} \right) \ldots \left({s - 1} \right)s}}} \right) \\ \mathcal{R} &=& \left({{r_{1}},{r_{2}}, \ldots,{r_{s}},{r_{11}}, \ldots,{r_{\left({s - 1} \right)s}}, \ldots,{r_{1 \ldots k}}, \ldots,{r_{\left({s - \left({k - 1} \right)} \right) \ldots \left({s - 1} \right)s}}} \right). \end{aligned}}  $$
  Here, *n*_1_,*m*_1_ and *n*_0_ stand for the total number of annotated genes in each generated expanded profile and *k* stands for the allowed maximum number of simultaneous annotation, *k*≤*s*, of the *s* annotated GO items^1^. Remember that *n*_1_ stands for the genes exclusively in the first list being compared, *m*_1_ for the genes only in the second list and *n*_0_ for the genes common to both lists, if any.
2. Build the finally compared profiles: $\widehat { \mathcal P} = \widehat { \mathcal P}_{1} + \widehat { \mathcal R}$ and $\widehat { \mathcal Q} = \widehat { \mathcal Q}_{1} + \widehat { \mathcal R}$, with *n*=*n*_1_+*n*_0_ and *m*=*m*_1_+*n*_0_.
3. “Contract” $\widehat { \mathcal P}$ and $\widehat { \mathcal Q}$ to obtain the basic profiles $\hat P$ and $\hat Q$.
4. Perform the equivalence test between these profiles and collect all desired statistics (e.g., if equivalence was declared or not in this single simulation iteration).

For simplicity, in this section we will not continue using the above notation for probability vectors like $\mathcal {P}_{1}$ (which on the other hand is useful to highlight simultaneous annotation of GO items) and we will simply designate them as *p*_1_,…,*p*_*g*_, where *g* corresponds to the vector length. In the simulations presented here, each one of its components *p*_*i*_ was obtained from a geometric model dependent only on a single parameter 0<*θ*<1:

$${p_{i}} = \frac{{\theta {{\left({1 - \theta} \right)}^{i - 1}}}}{{1 - {{\left({1 - \theta} \right)}^{g}}}}, \hspace{0.5cm} i = 1, \dots, g.$$

Obviously, making the simulated profiles dependent on a single parameter in this way greatly restricts the possible scenarios to be simulated. On the other hand, it allows for an important simplification in order to trace the probability of declaring equivalence as a single function of the simulated true squared Euclidean distance: given a value of *θ*, $\mathcal {P}_{1}$ was obtained from *θ*, $\mathcal {Q}_{1}$ was obtained from 1−*θ* (say, *q*_*i*_=(1−*θ*)*θ*^*i*−1^/(1−*θ*^*g*^)) and $\mathcal {R}$ from a fixed *θ*_0_ value, independently of *θ*.

For fixed *n*,*m*,*n*_0_,*θ*_0_, *s* and *k* values, the squared Euclidean distance is a function of *θ*, *d*=*D*(*θ*). Given a set of desired *d* values (some less than *Δ*, i.e. in a scenario of false null hypothesis in the equivalence test; some greater than *Δ*, i.e. true null hypothesis; and one with *d*=*Δ*, just on the limit of equivalence), numerically solving the equation *D*(*θ*)=*d* we can obtain the required *θ* values and thus a set of profiles to simulate these “population” distances. Then simulations may be performed in order to obtain the probability of rejecting the null hypothesis of non–equivalence, i.e. to obtain the power curve of the test, as a function of the *d* parameter.

**Fig. 1:**
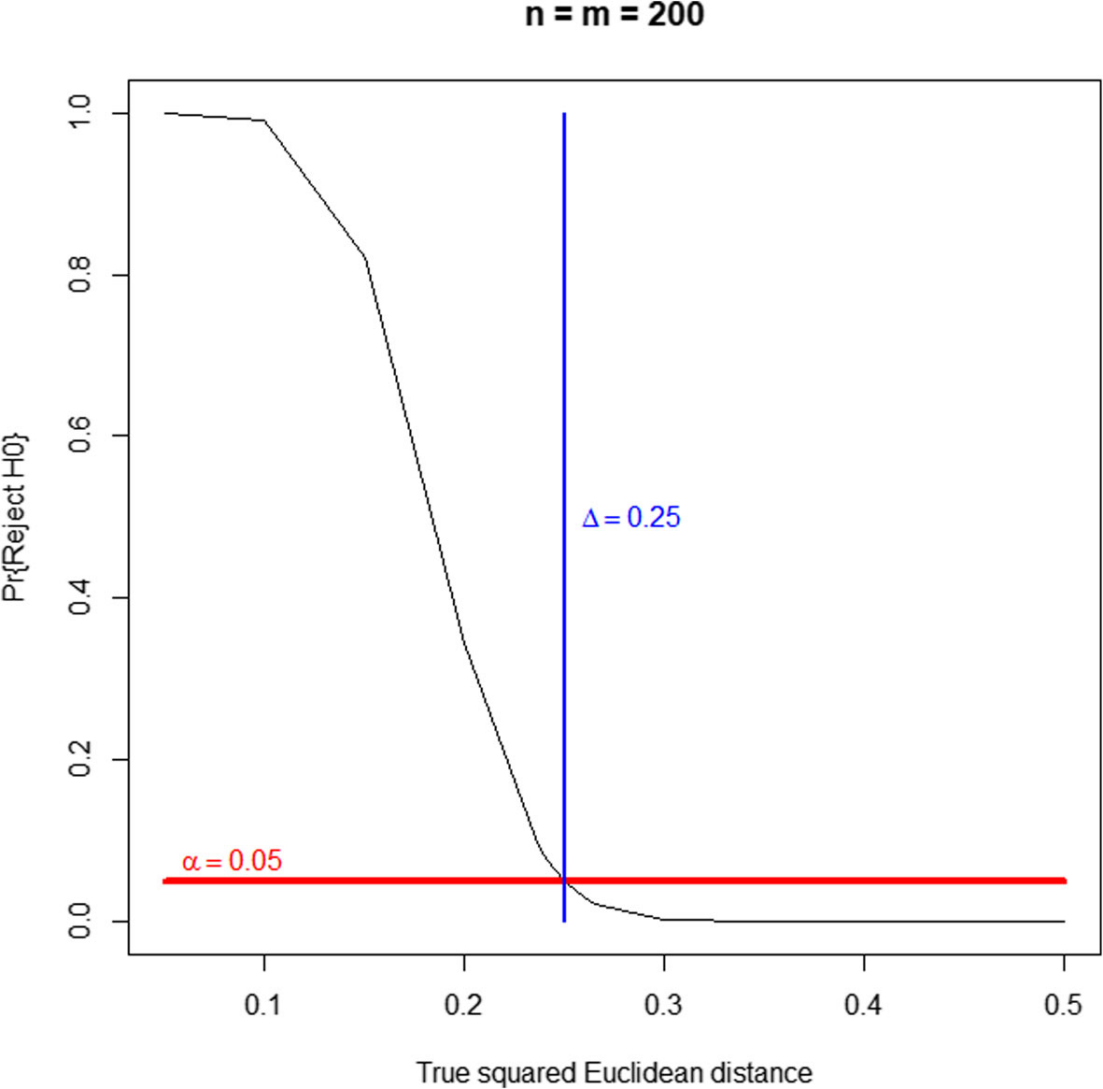
Power curve of the equivalence test, as a function of the true squared Euclidean distance. Balanced case of two gene lists of size 200 with 20 genes in common. Equivalence limit at *Δ*=0.25. The null hypothesis of the equivalence test states that the true squared Euclidean distance, *d*, is greater than or equal to *Δ*, that is to say, that both lists are sufficiently dissimilar according to the *Δ* limit criterion. Thus, rejecting this hypothesis corresponds to declaring equivalence. When the true simulated distance is *d*<*Δ*, not rejecting the null hypothesis not declaring equivalence) corresponds to a false negative. When *d*≥*Δ*, declaring equivalence is a false positive

**Fig. 2:**
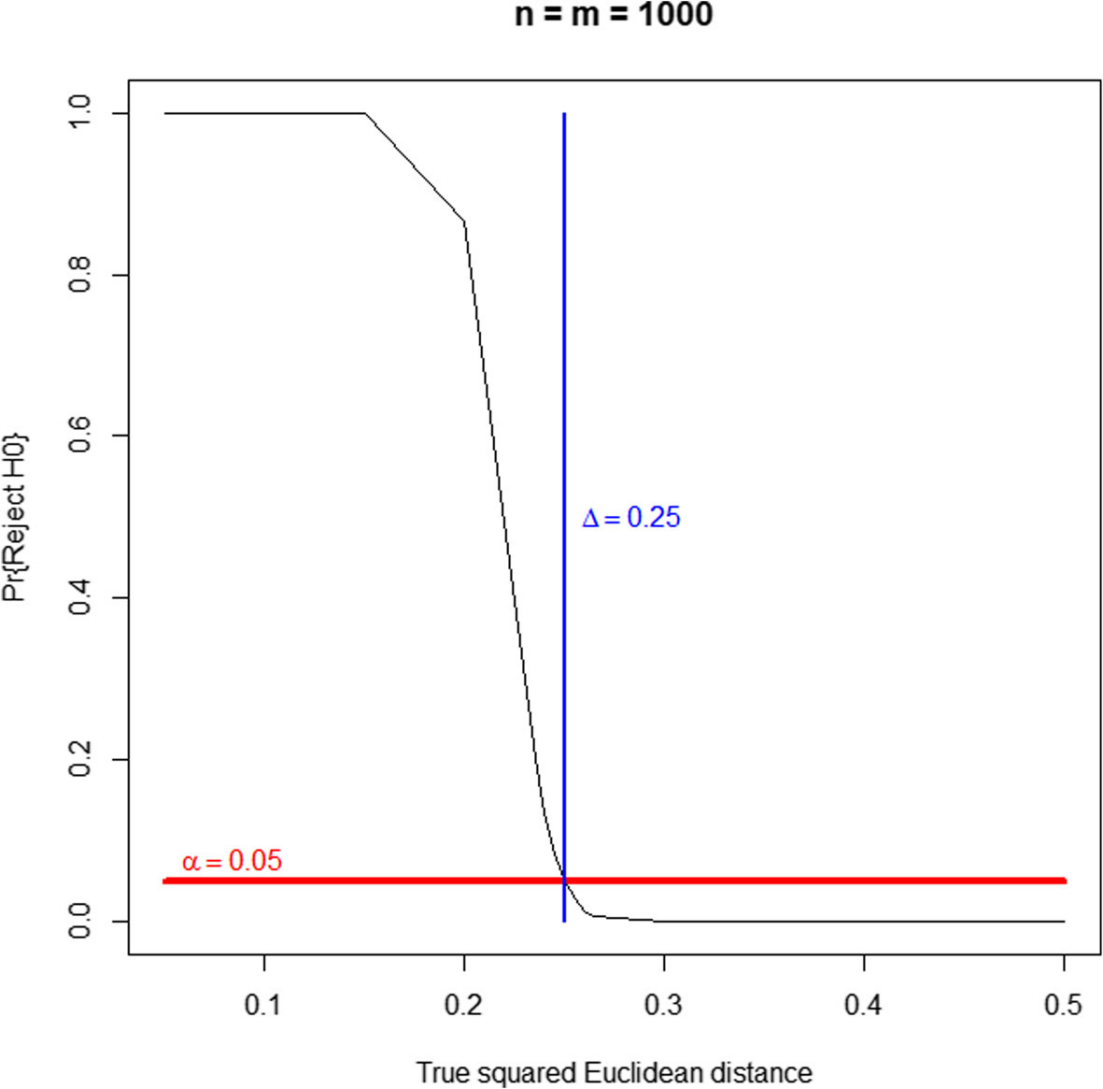
Power curve of the equivalence test, as a function of the true squared Euclidean distance. Balanced case of two gene lists of size 1,000 with 100 genes in common. Equivalence limit at *Δ*=0.25. The null hypothesis of the equivalence test states that the true squared Euclidean distance, *d*, is greater than or equal to *Δ*, that is to say, that both lists are sufficiently dissimilar according to the *Δ* limit criterion. Thus, rejecting this hypothesis corresponds to declaring equivalence. When the true simulated distance is *d*<*Δ*, not rejecting the null hypothesis (not declaring equivalence) corresponds to a false negative. When *d*≥*Δ*, declaring equivalence is a false positive

**Fig. 3:**
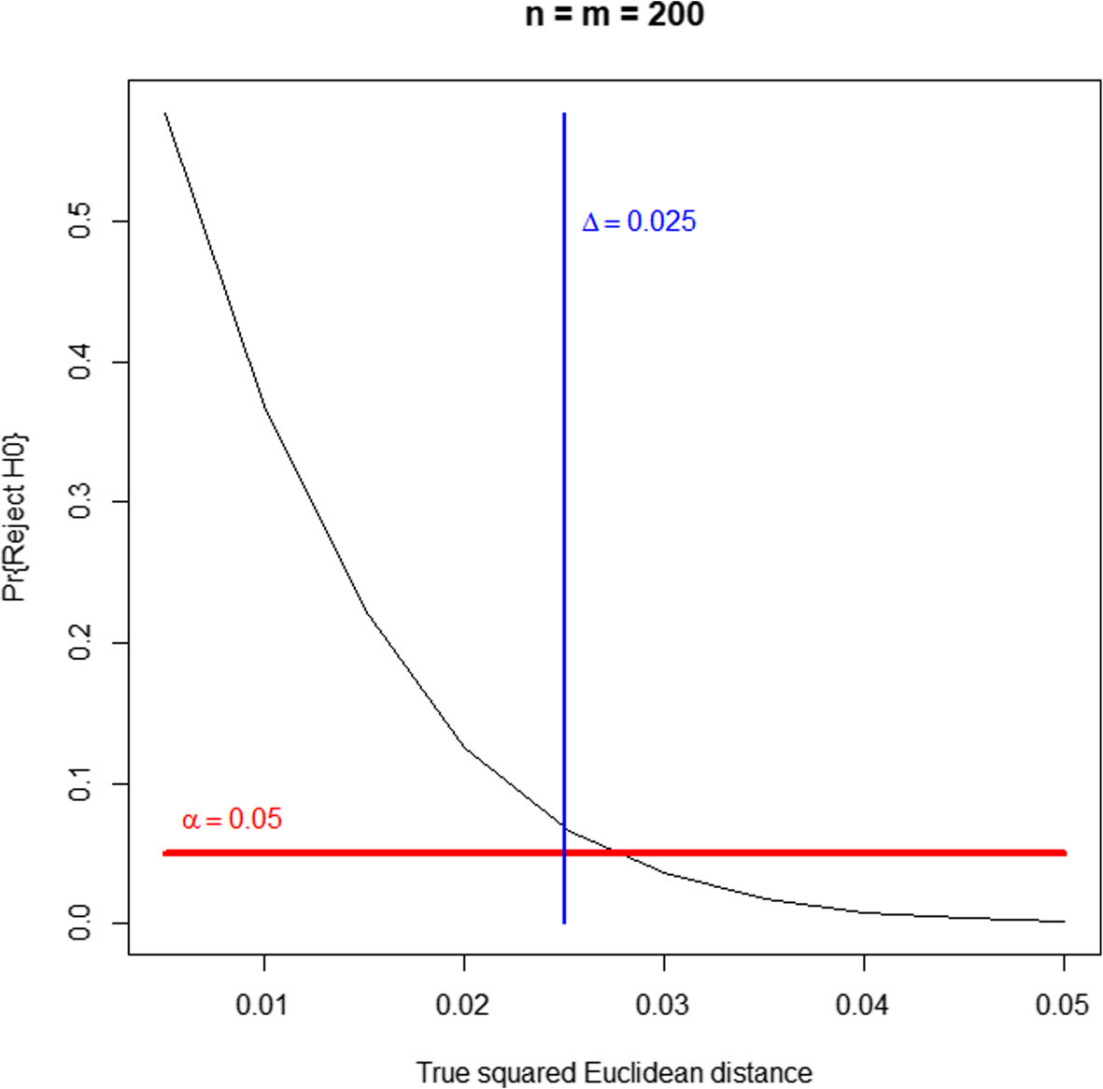
Power curve of the equivalence test, as a function of the true squared Euclidean distance. Balanced case of two gene lists of size 200 with 20 genes in common. Equivalence limit at *Δ*=0.025. The null hypothesis of the equivalence test states that the true squared Euclidean distance, *d*, is greater than or equal to *Δ*, that is to say, that both lists are sufficiently dissimilar according to the *Δ* limit criterion. Thus, rejecting this hypothesis corresponds to declaring equivalence. When the true simulated distance is *d*<*Δ*, not rejecting the null hypothesis (not declaring equivalence) corresponds to a false negative. When *d*≥*Δ*, declaring equivalence is a false positive

A well-behaved (unbiased) equivalence test should reject the null hypothesis of nonequivalence with a probability greater than *α* for parameter values *d*<*Δ*, with a probability smaller than *α* for *d*>*Δ* and, ideally, with a probability of *α* when *d*=*Δ*. Figures 1, 2 and 3 display the probability of rejecting the null hypothesis of nonequivalence (i.e., the probability of declaring equivalence) as a function of the true simulated squared Euclidean distance. They correspond to three sample sizes and the threshold of equivalence scenarios. They show that the profiles equivalence test is generally valid. As a consequence of the Bonferroni-Holm method validity (as a way to protect FWER), the equivalence test for more than two lists is also generally valid. For very small equivalence limits (near zero), there is some type I error inflation, with probabilities slightly over the nominal significance level, e.g. values around 0.06 for significance levels of 0.05. As supplementary material (see Additional file 5) we provide three bar plots representing the probabilities of false negatives and false positives corresponding to Figs. 1, 2 and 3, respectively. When the true simulated distance is *d*<*Δ*, not rejecting the null hypothesis (not declaring equivalence) corresponds to a false negative. When *d*≥*Δ*, declaring equivalence is a false positive.

### Software

The analysis of functional profiles, that is, the computation of profiles and paired tests of difference or equivalence has been implemented in the R package goProfiles available in Bioconductor [20].

The current version of the package (1.44 or higher) implements the capabilities described in this paper. That is, given a set of gene lists –provided as Entrez identifiers– one can: (i) compute a dissimilarity matrix between the corresponding profiles at a given level of any ontology; (ii) apply the algorithm described in the previous section to determine the equivalence level at which any pair of lists can be considered equivalent; and (iii) visualize the associated dendrogram with the chosen method.

The package is available in github (https://github.com/alexsanchezpla/goProfiles) and in Bioconductor (http://bioconductor.org/packages/goProfiles/).

## Examples

We have selected two prototypical situations where we believe that using the approach described in the paper can be useful for simplifying the data the researchers are working with, or even for shedding new light on their meaning.

### Equivalence analysis of kidney gene lists

Organ rejection diagnosis is mainly based on the study of tissue biopsies (e.g. renal, lung, heart or liver) but, unfortunately, the lesions observed using conventional histology are often not specific for the underlying mechanism since histological lesions (e.g., interstitial inflammation in renal biopsies) maybe driven by different processes. The molecular mechanisms operating in human organ transplant rejection are best inferred from the mRNAs expressed in biopsies because the corresponding proteins often have low expression and short half–lives, while small noncoding RNAs lack specificity. The study of associations should be characterized in a population that rigorously identifies the different mechanism participating in organ rejection, i.e. T cell–mediated and antibody–mediated rejection (TCMR and ABMR). Associations can be universal (both types of rejection), TCMR–selective, or ABMR–selective. It has been proposed that top universal transcripts are gamma–interferon–inducible and transcripts shared by effector T cells and NK cells. TCMR–selective transcripts are expressed in activated effector T cells or gamma–interferon–induced macrophages while ABMR–selective transcripts are expressed in NK cells and endothelial cells. Transcript associations are highly reproducible between biopsy sets when the same rejection definitions, algorithm, and technology are applied, but exact ranks will vary. Although rejection–associated transcripts are never completely rejection–specific because they are shared with the stereotyped response–to–injury and innate immunity, transcriptomic analysis using pathogenesis–based transcripts contributes to a better characterization of mechanisms leading to organ dysfunction.

This example uses a set of gene lists generically described as “PBTs” (pathogenesis–based transcript sets) studied by [22] and available at https://www.ualberta.ca/medicine/institutes-centres-groups/atagc/research/gene-lists and as supplementary material^2^ (See Additional file 1).

Each list consists of a series of probeset identifiers from hgu133plus2 Affymetrix expression microarrays that have been selected in distinct studies referenced in [22]. For this example the probesets have been preprocessed as follows:

- Affymetrix identifiers have been converted into Entrez identifiers using Biomart.
- When several probesets had the same identifier, this appeared only once in the list.

This preprocessing yields five gene lists described in Table 1 where, for each list, we provide its PBT abbreviation, the number of unique Entrez identifiers and a short description.

**Table 1:** Kidney gene lists

PBT	Size	PBT Name	Biological Description
ENDAT	114	Endothelium-associated transcripts	Microcirculation response to injury
IRITD3	313	Injury- and repair-induced transcripts day 3	Active injury–repair response: ’injury-up’ Increased in isografts peaking day 3
IRITD5	221	Injury- and repair-induced transcripts day 5	Active injury–repair response: ’injury-up’ Increased in isografts peaking day 5
KT1	574	Kidney transcripts—set 1	Active injury–repair response: ’injury-down’ Parenchymal transcripts
KT1.1	119	Kidney transcripts - Set 1.1	Humanized mouse kidney selective transcripts reduced >90% in day 21 mouse allografts

Pathogenesis-based transcript sets or “PBTs”. The lists have been selected from the datasets available in the file “PBTs_all_affy” downloaded from the url: https://www.ualberta.ca/medicine/institutes-centres-groups/atagc/research/gene-lists. Only lists with more than 100 transcripts have been retained. Transcript names have been converted from probeset identifiers into Entrez identifiers. Given that several probesets are associated to the same Entrez ID the final gene lists are usually shorter than transcript lists. Since the file has been downloaded the web page has changed and this file is not available anymore, although new version of the file can be found in the site

**Fig. 4:**
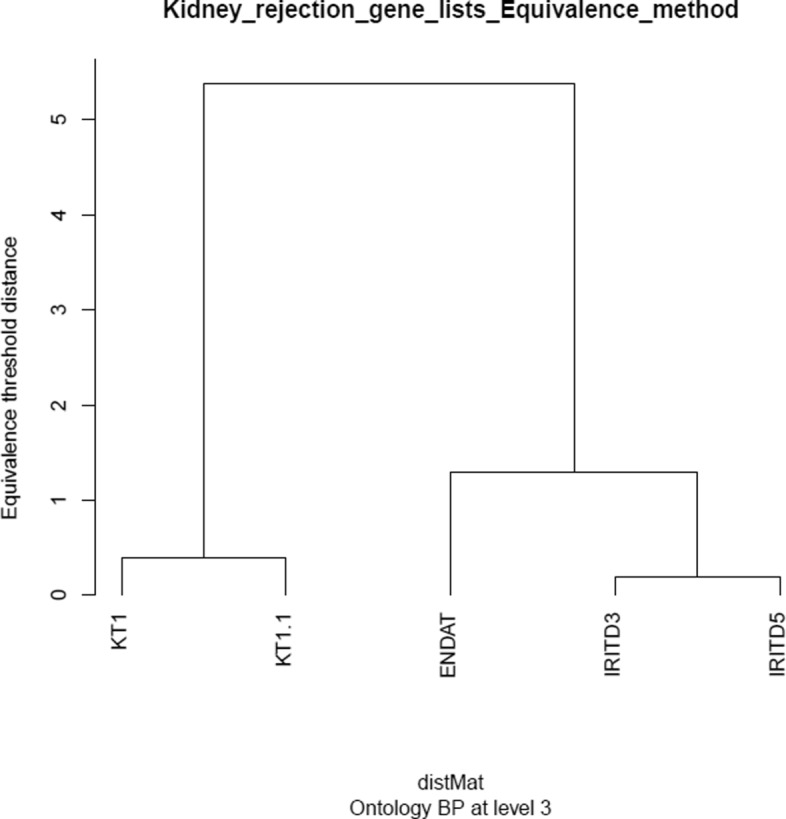
Dendrogram produced from the equivalence analysis of kidney gene lists made at level 3 of the BP ontology. The lists are grouped naturally depending on the type of process on which the genes of the lists are involved. See Additional file 3 for supplementary figures at levels 2 to 8 of all three (MF, CC and BP) ontologies

Equivalence analysis of these gene lists can be easily performed using functions in the goProfiles package (see the detailed analysis example in Additional file 3). A “standard” analysis has been performed that consists of computing the dissimilarity matrix of equivalence thresholds and building a dendrogram from this for the three ontologies at levels from 2 to 8. These dendrograms can be viewed in Fig. 4 (made at level 3 of the BP ontology) and in Additional file 3 (levels 2 to 8 of all three ontologies).

As can be seen in the plots (Fig. 4 and supplementary figures in Additional file 3) the grouping produced has the same structure for all lists: kidney transcripts on one side and endothelial and injury transcripts on the other, the latter being more similar to each other than to endothelial transcripts. These groupings are not surprising because each type of gene is involved in different biological processes but they suggest that groupings observed in other settings, where the relation between the lists is not obvious, can also be considered as reasonable (see next example).

### Equivalence analysis of cancer gene lists

As a second example, we consider a series of lists that have been obtained from Bushman lab (http://www.bushmanlab.org/links/genelists). The lists contain Entrez identifiers for each gene so the only preprocessing consisted of removing one list that contained less than 100 genes. Table 2 contains, for each list, the name, the number of genes, the species and a short description. Citing the researcher’s description of the lists they are *collections of cancer-related genes that were used to generate a comprehensive list (allOnco) that is comprised of the union of all lists*. In this case, we do not have any a priori expectations of which lists should be equivalent to which but, instead, we can rely on equivalence analysis to help answer the question “up to what point can these lists be considered equivalent so that they can be merged into a single list?”

**Table 2:** Cancer Gene Lists

Set	Size	Species	Description
Atlas	989	human	Genes: hybrid gene found in at least one cancer case, or gene amplification or homozygous deletion found in a significant subset of cases in a given cancer-type.
CANgenes	189	human	191 common genes that were mutated at significant frequency in all tumors of human breast and colorectal cancers.
CIS (RTCGD)	587	multiple	Retroviral insertional mutagenesis in mouse hematopoietic tumors.
Miscellaneous	187	multiple	From Cold Spring Harbor Retroviruses Chapter on Oncogenes, an early version of the CIS database, a list from Dr. Tony Hunter, and misc. additions from the literature.
Sanger	452	human	Compilation from literature: “genes that are mutated and causally implicated in cancer development”
Vogelstein	420	human	Cancer genes related to chromosomal breakpoints
Waldman	455	Human	Gene set is from the Waldman gene database and lists cancer genes sorted by chromosomal locus and includes links to OMIM.

Cancer related genes that were used to generate a comprehensive list (allOnco) that is comprised of the union of all lists The lists have been selected from the datasets available in the file “allOnco.tsv” downloaded from the url http://www.bushmanlab.org/links/genelists. Only lists with more than 50 genes have been retained. Since the file has been downloaded the web page has changed and this file is not available anymore, although new version of the file can be found in the site

**Fig. 5:**
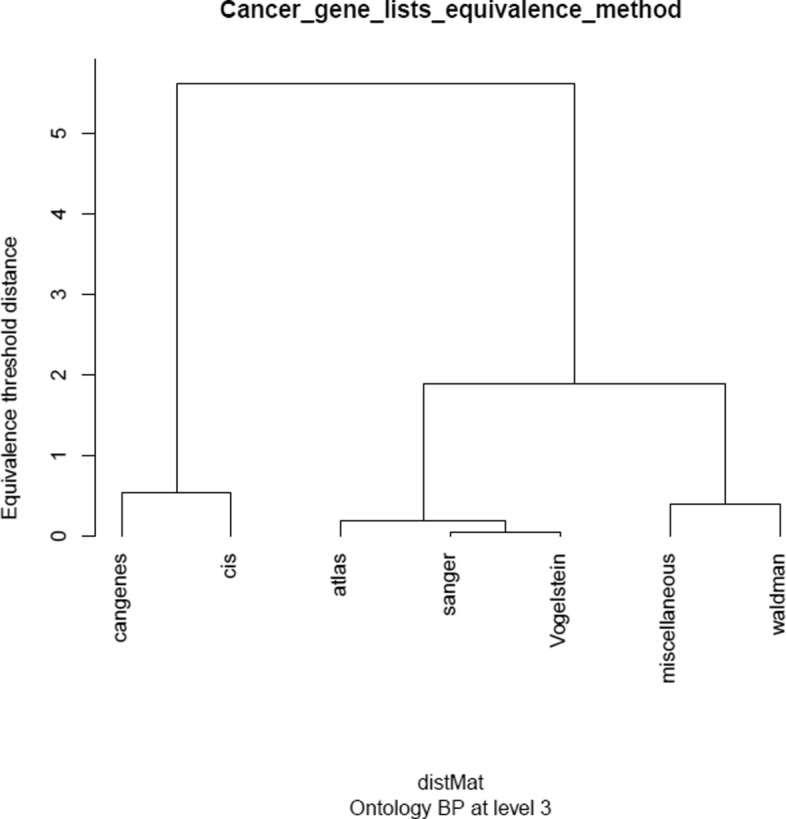
Dendrogram produced from the equivalence analysis of cancer gene lists made at level 3 of the BP ontology. In this case there are no natural groupings but instead, the dendrogram may be used to suggest which lists may be combined more reasonably than others. See Additional file 4 for supplementary figures at levels 2 to 8 of all three (MF, CC and BP) ontologies

Figure 5 and supplementary figures in Additional file 4 show the results of equivalence analysis. Interestingly the lists tend to group consistently within the ontologies –groupings at distinct levels of the ontologies are almost identical– but these groupings can change from one ontology to another, which is not strange because they refer to different concepts. Depending on what the goal of merging the gene lists is the different groupings of each ontology can be used as a guide to decide whether a given dataset should be included or not in a common list. For instance depending on whether what one wishes to obtain is a heterogeneous or a homogeneous list one could decide to include groups that are separated by a higher threshold or, instead, that are near each other in the dendrogram.

## Discussion and limitations

In this paper, a method for dealing with the problem of simultaneously comparing multiple feature lists has been introduced. The method is based on comparing feature lists by means of their projections at fixed Gene Ontology levels. These projections are called “functional profiles”. One innovative characteristic is that the comparison is done by means of equivalence tests, which are aimed at rejecting a null hypothesis of nonequivalence, which means that it can be stated (when this null hypothesis is rejected) that two lists are “equivalent at a certain threshold”. This is a better statement than simply saying that “there is no evidence of difference or dependency” (which does not mean that they are equal or independent), as would be the case if a “standard” difference or dependency test was performed. The fact that it relies upon equivalence makes it particularly interesting for data integration problems, such as for the cancer gene lists presented in the examples.

Following a reviewer suggestion we compared the equivalence test with a standard test of positive dependency. Although appealing, this test is not adequate to solve the proposed problem. Its limitations are shown in the supplementary material (see Additional file 6).

The examples have shown that the method behaves consistently with expected similarities. That is, it tends to consider equivalent at lower threshold feature lists that – even if they have few elements in common – are expected to be easily declared equivalent. One can interpret that this happens because they are associated with the same or similar biological processes, such as with injury-related PBTs in the kidney gene lists examples. This, of course, suggests that when two lists, whose relationship is not known, show up as equivalent they can be considered similar enough.

The method is not free from limitations. For instance an obvious concern may be the fact that comparisons are made separately at each level of each ontology, which means, for instance, that if one considers three levels (e.g. 2, 3 and 4) of the three ontologies (CC, BP and MF) one ends up with nine comparisons that one may want to combine a posteriori. In spite of its apparent inconvenience, this may be seen as useful – firstly because in general, a good consistency and reproducibility are observed between different levels of the same ontology; that is, they yield generally the same classification, and small differences observed may be mostly attributable to list size. In some cases, there may be differences between ontologies, but this is not a serious drawback either, because the distinct ontologies reflect distinct biological concepts, so differences can be considered reasonable. If all the comparisons were compacted into a single one this variability might be lost, which could hamper the interpretation of the results.

Another issue to be accounted for is computational efficiency. This depends on many factors, such as the computer where the programs are run, the number of lists to compare, the size (number of features) of these lists and the number of GO nodes in the profiles being compared. A small simulation study has been performed to provide information about execution times in a realistic scenario: using a basic bioinformatic station (i7-4790 processor with 8GB of RAM running R 3.4 on 64-bit Windows 7 Enterprise) the process of determining the equivalence of a certain number of random gene lists has been executed repeatedly. This has been done using the equivClust function of the current version (1.44.0) of the goProfiles package. Times were measured by means of the R package microbenchmark, which provides summary statistics for the running time. The number of lists compared in the simulations was 5, 10, 25 and 50. For simplicity pairs of lists being compared were set to have the same size. The sizes considered were 100, 200, 1000, 2000 and 5000. The comparison was made at levels 2 and 3 of the “Biological Process” (BP) ontology.

Table 1 and Fig. 1 in Additional file 7 show the summaries of the execution times in five replicates (if the number of genes was 5000 only one replicate was used). It can be seen that, at level 3, the required time to build an equivalence dendrogram for 50 gene lists and 5000 genes is more than 32 hours (115858.16/3600) which is clearly not assumable for ordinary calculations.

## Conclusions

The method introduced in this work provides a way for classifying sets of genes or other features based on equivalence testing on their corresponding functional profiles. It can be viewed as an extension of the goProfiles methodology [19, 20] introduced previously and it is statistically well grounded. Standard visualizations, such as dendrograms, can be used to depict the classification results. The method has a wide applicability and has been used in a variety of problems such as deciding whether a series of datasets generating the lists can be combined, or in regard to classifying the lists in a collection of signatures from most to least similar, in the sense of equivalence.

## Additional files

Additional file 1 Comma separated file containg original data for the Kidney lists example. Each list is a collection of affymetrix probesets from hgu133plus2 arrays. The file was downloaded from the url: https://www.ualberta.ca/medicine/institutes-centres-groups/atagc/research/gene-lists. (TSV 61 kb)

Additional file 2 Comma separated file containg original data for the Cancer lists example. Each list is a collection of Entrez identifiers from human or from another species. The file was downloaded from the url: http://www.bushmanlab.org/links/genelists. (CSV 284 kb)

Additional file 3 This file contains an extended version of the Kidney data analysis example. Plots of the dendrograms produced up to the 8th level of the GO are shown. Analysis of the data based on Semantic Similarity (SS) are provided. Informal comparisons among results obtained using distinct SS measures and with results obtained with goprofiles are presented. Each plot is in a separate page to facilitate visualization. (PDF 214 kb)

Additional file 4 This file contains an extended version of the Cancer data analysis example. Plots of the dendrograms produced up to the 8th level of the GO are shown. Analysis of the data based on Semantic Similarity (SS) are provided. Informal comparisons among results obtained using distinct SS measures and with results obtained with goprofiles are presented. Each plot is in a separate page to facilitate visualization. (PDF 187 kb)

Additional file 5 The simulation study performed shows the ROC curves but a reviewer suggested that depicting False Positive and False Negative rates could also be interesting. This file shows three plots with FN (in blue) and FP (in red), as a function of some values of the true squared Euclidean distance. The plots differ in the total number of genes and the number of genes in common between the three lists. (PDF 30 kb)

Additional file 6 Comparison between the equivalence test with a standard test of positive dependency suggested by a reviewer. (PDF 348 kb)

Additional file 7 Summary results from a small simulation study performed to provide information about execution times in a realistic scenario. (PDF 166 kb)

## Abbreviations

BP: Biological Process Ontology
CC: Cellular Component Ontology
FDR: False Discovery Rate
FWER: Family Wise Error Rate
GO: Gene Ontology
MF: Molecular Function Ontology
MsigDB: Molecular Signature Databases
PBTS: Pathogenic-Based Transcript Set

## Footnotes

1 For large values of *s*, e.g., *s*=50 or more, it is advisable to take *k*<*s* to avoid managing extremely large profile vectors

2 Web pages may change and links become unavailable. To avoid these problems the datasets used in the examples have been downloaded from their public locations and added as supplementary materials
