# Supplementary material for: An equivalence approach to the integrative analysis of feature lists
Source: BMC Bioinformatics. 2019 Aug 27;20:441. doi: 10.1186/s12859-019-3008-x (PMC6712676; doi:10.1186/s12859-019-3008-x)
Supplement: Supplementary file 3 — This file contains an extended version of the Kidney data analysis example. Plots of the dendrograms produced up to the 8th level of the GO are shown. Analysis of the data based on Semantic Similarity (SS) are provided. Informal comparisons among results obtained using distinct SS measures and with results obtained with goprofiles are presented. Each plot is in a separate page to facilitate visualization. (PDF 214 kb) [file 12859_2019_3008_MOESM3_ESM.pdf]

Equivalence Analysis of Pathogenesis-Based Transcript (PBT) Gene Lists

Supplementary materials to Sanchez-Pla, A., Salicru, M. and Ocaña, J. An equivalence approach to the integrative analysis of features lists. BMC Bioinformatics

Organ rejection diagnosis is mainly based on the study of tissue biopsies (e.g. renal, lung, heart or liver) but, unfortunately, the lesions observed by conventional histology are often not specific for the underlying mechanism since histological lesions (e.g., interstitial inflammation in renal biopsies) may be driven by different processes. The molecular mechanisms operating in human organ transplant rejection are best inferred from the mRNAs expressed in biopsies because the corresponding proteins often have low expression and short half-lives, while small non-coding RNAs lack specificity. The study of associations should be characterized in a population that rigorously identifies the different mechanism participating in organ rejection, that is, T cell-mediated and antibody-mediated rejection (TCMR and ABMR). Associations can be universal (both types of rejection), TCMR-selective, or ABMR-selective. It has been proposed that top universal transcripts are gamma-interferon inducible and transcripts shared by effector T cells and NK cells. TCMR-selective transcripts are expressed in activated effector T cells or gamma interferon-induced macrophages while ABMR-selective transcripts are expressed in NK cells and en- dothelial cells. Transcript associations are highly reproducible between biopsy sets when the same rejection definitions, algorithm, and technology are applied, but exact ranks will vary. Despite rejection-associated transcripts are never completely rejection-specific because they are shared with the stereotyped response-to-injury and innate immunity, transcriptomic analysis using pathogenesis-based transcripts contributes to a better characterization of mechanisms leading to organ dysfunction.

Many studies have been performed to identify sets of genes that can be associated with pathogenic processes that can lead to kidney failure. We work here with a list of gene lists generically described as "PBTs" (Pathogenic Based Transcript Sets) available at https://www.ualberta.ca/medicine/institutes-centers-groups/atagc/research/gene-list and as supplementary material[1].

Each list consists in a series of probeset identifiers from `hgu133plus2` Affymetrix expression microarrays that have been selected in distinct studies. For this example the probesets have been preprocessed as follows:

- Affymetrix identifiers have been converted into `Entrez` identifiers with Biomart.
- When several probesets had the same identifier this appeared only once in the list.

Equivalence analysis of the resulting gene lists can be easily performed using functions in the `goProfiles` package. A "standard" analysis has been performed which consists of computing the dissimilarity matrix of equivalence thresholds and building a dendrogram (here using the maximum distance, or complete, method) for the three ontologies at levels 2 to 8. Provided the asymptotic nature of the tests considered here, only the five lists with almost 100 annotated genes

[1] Web pages may change and links become unavailable. To avoid these problems the datasets used in the examples have been downloaded from their public locations and added as supplementary materials

at the less restrictive GO level (level 2) have been included in the analysis. They are described in supplementary table 1 where, for each list, we provide its PBT abbreviation, the number of unique Entrez Ids and a short description.

Table 1: Kidney rejection after transplantation related gene lists.

PBT	Size	PBT Name	Biological Description
ENDAT	114	Endothelium-associated transcripts	Microcirculation response to injury
IRITD3	313	Injury- and repair-induced transcripts day 3	Active injury-repair response: 'injury-up' Increased in isografts peaking day 3
IRITD5	221	Injury- and repair-induced transcripts day 5	Active injury-repair response: 'injury-up' Increased in isografts peaking day 5
KT1	574	Kidney transcripts-set 1	Active injury-repair response: 'injury-down' Parenchymal transcripts
KT1.1	119	Kidney transcripts - Set 1.1	Humanized mouse kidney selective transcripts reduced >90% in day 21 mouse allografts

```
> library(goProfiles)
> load("pbtsGeneLists2.rda")
> sapply(pbtGeneLists2, length)

 ENDAT IRITD3 IRITD5    KT1  KT1.1
   114    313    221    574    119

> # Genes in common to each pair of lists:
> lstNams <- names(pbtGeneLists2)
> for (i in 2:length(pbtGeneLists2)) {
+   for (j in 1:(i-1)) {
+     cat(lstNams[i], "&", lstNams[j],
+         length(intersect(pbtGeneLists2[[i]], pbtGeneLists2[[j]])),
+         "common genes of ", length(pbtGeneLists2[[i]]), length(pbtGeneLists2[[j]]),
+         "\n")
+   }
+ }

IRITD3 & ENDAT 7 common genes of  313 114
IRITD5 & ENDAT 4 common genes of  221 114
IRITD5 & IRITD3 4 common genes of  221 313
KT1 & ENDAT 9 common genes of  574 114
KT1 & IRITD3 0 common genes of  574 313
```

```
KT1 & IRITD5 1 common genes of  574 221
KT1.1 & ENDAT 0 common genes of  119 114
KT1.1 & IRITD3 0 common genes of  119 313
KT1.1 & IRITD5 0 common genes of  119 221
KT1.1 & KT1 109 common genes of  119 574
```

```
> # Number of annotated genes in each ontology and GO level:
> # (quite time consuming, to save time results are included below)
> # for (lev in 2:16) {
> #   cat("level ", lev, "\n")
> #   profsList <- lapply(pbtGeneLists2,
> #                         expandedProfile, level = lev, orgPackage = "org.Hs.eg.db")
> #   print(sapply(profsList, function(ontoProf){
> #     sapply(ontoProf, ngenes)
> #   }))
> # }
> # Equivalence analysis from GO levels 2 to 8 and for all ontologies:
> # (next sentence is considerably time consuming, to speed processing,
> # you may directly go to
> # load(file = "kidney_rejection_lists_ etc. uncomment, and run it)
>
> genListsClusters <- iterEquivClust(
+   pbtGeneLists2, ontoLevels = 2:8,
+   jobName = "kidney_rejection_gene_lists_equivalence_clustering_levels2to8",
+   ylab = "Equivalence threshold distance",
+   orgPackage="org.Hs.eg.db", method = "complete")
```

```
 kidney_rejection_gene_lists_equivalence_clustering_levels2to8 Ontology BP at level 2

Building marginal profiles:

Building profile for list  ENDAT
Building profile for list  IRITD3
Building profile for list  IRITD5
Building profile for list  KT1
Building profile for list  KT1.1

Building intersection profiles:

IRITD3,ENDAT |
IRITD5,ENDAT |IRITD5,IRITD3|
KT1   ,ENDAT |KT1   ,IRITD3|KT1   ,IRITD5|
KT1.1 ,ENDAT |KT1.1 ,IRITD3|KT1.1 ,IRITD5|KT1.1 ,KT1   |

Performing all equivalence tests:
```

```
IRITD3,ENDAT |
IRITD5,ENDAT |IRITD5,IRITD3|
KT1    ,ENDAT |KT1    ,IRITD3|KT1    ,IRITD5|
KT1.1  ,ENDAT |KT1.1  ,IRITD3|KT1.1  ,IRITD5|KT1.1  ,KT1    |

 kidney_rejection_gene_lists_equivalence_clustering_levels2to8 Ontology BP at level 3

Building marginal profiles:

Etc. Truncated script output...


> save(genListsClusters,
+      file = paste0(attr(genListsClusters, "jobName"), ".rda", sep =""))
> # load("kidney_rejection_gene_lists_equivalence_clustering_levels2to8.rda")
>
> # Generate a pdf file with all equivalence clusters:
> equivClust2pdf(genListsClusters,
+                jobName = "Kidney_rejection_gene_lists_Equivalence_method")
```

As can be seen in the plots, the end groupings share similar patterns for all ontologies and levels: Kidney transcripts by one side and endothelial and injury transcripts by the other, the later more similar to each other than to endothelial. These groupings are not surprising because each type of genes is involved in different biological processes but they suggest that groupings observed in other settings, where the relation between the lists is not obvious, can also be considered as reasonable.

Despite these general trends, there is some variability between the clusters obtained at different levels of the same ontology. In our opinion, a trade-off between the need for statistical validity vs the need for interesting biological information must be considered. Provided its asymptotic inferential character (that is to say, more sample size -i.e., more total annotation- would imply more reliability in the inferences), one may expect more stability in the results for large sample sizes. Total annotation may decline if we require more specificity to GO terms, if we go deep in the GO. On the other hand, more specificity provides more interesting biological information; at lower levels the GO terms under consideration may be too general. There is considerable stability with respect to the final groupings at intermediate GO levels, from 4 to 6. Not surprisingly, this stability is partially broken from level 7, specially for the MF ontology, where the total annotation number for lists ENDAT and KT1.1 greatly falls. These two lists have the greatest indeterminacy with respect their group membership, not only among GO levels in the equivalence method but also among other approaches like semantic similarity methods, which are farm from similar among them as is discussed below.

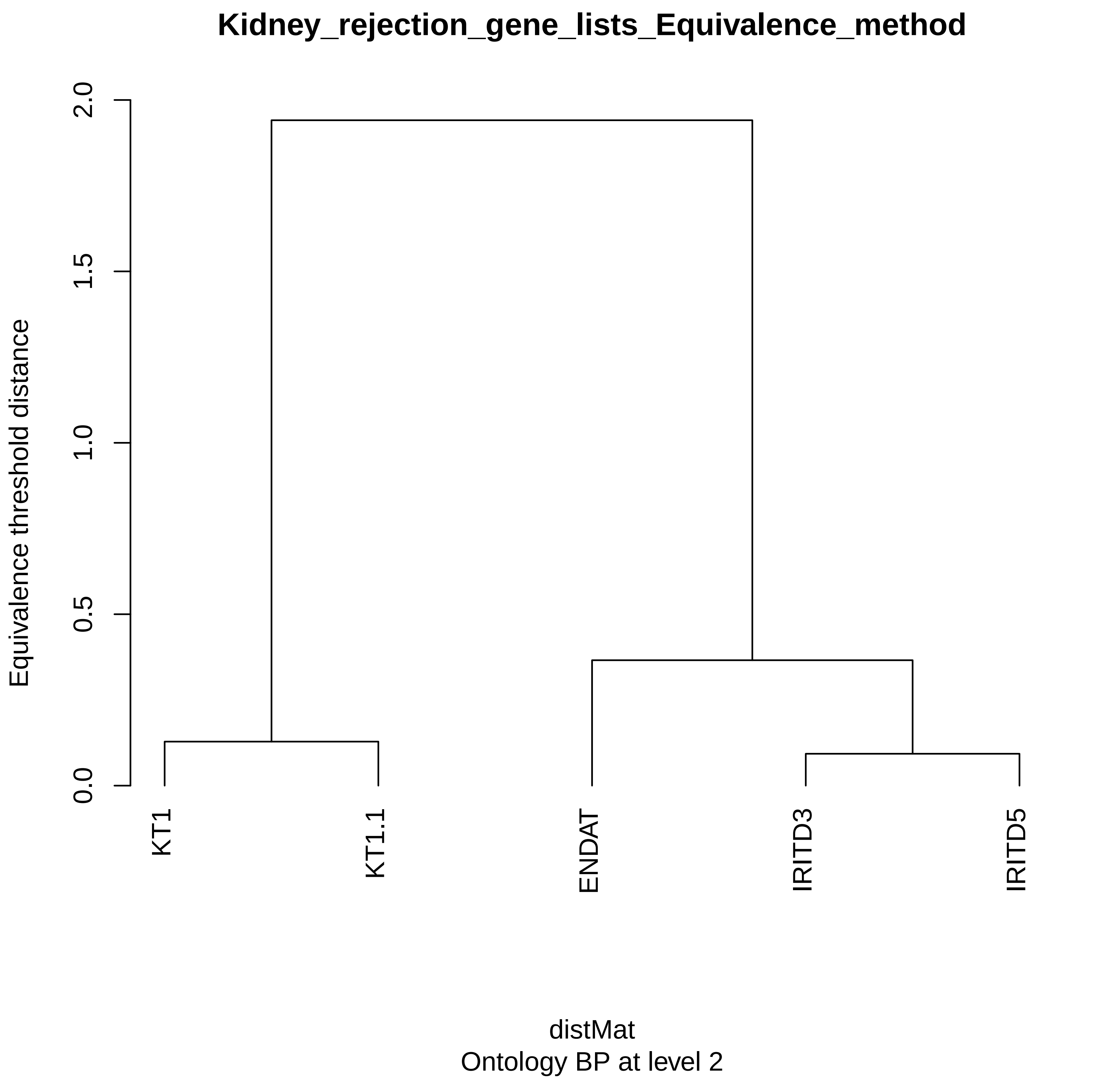
Kidney_rejection_gene_lists_Equivalence_method
Equivalence threshold distance
0.0
0.5
1.0
1.5
2.0
KT1
KT1.1
ENDAT
IRITD3
IRITD5
distMat
Ontology BP at level 2

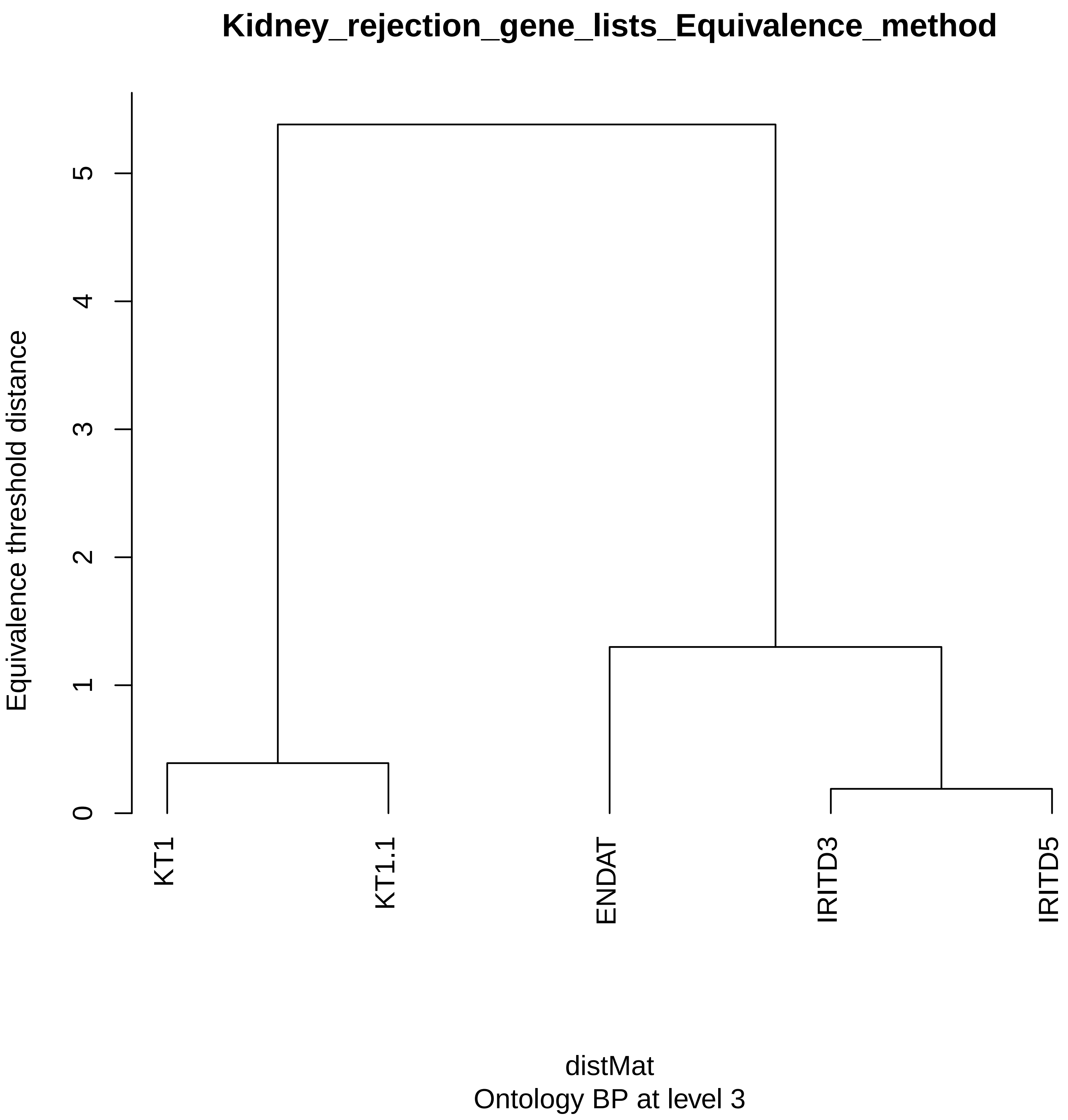

Kidney_rejection_gene_lists_Equivalence_method
Equivalence threshold distance
0
1
2
3
4
5
KT1
KT1.1
ENDAT
IRITD3
IRITD5
distMat
Ontology BP at level 3

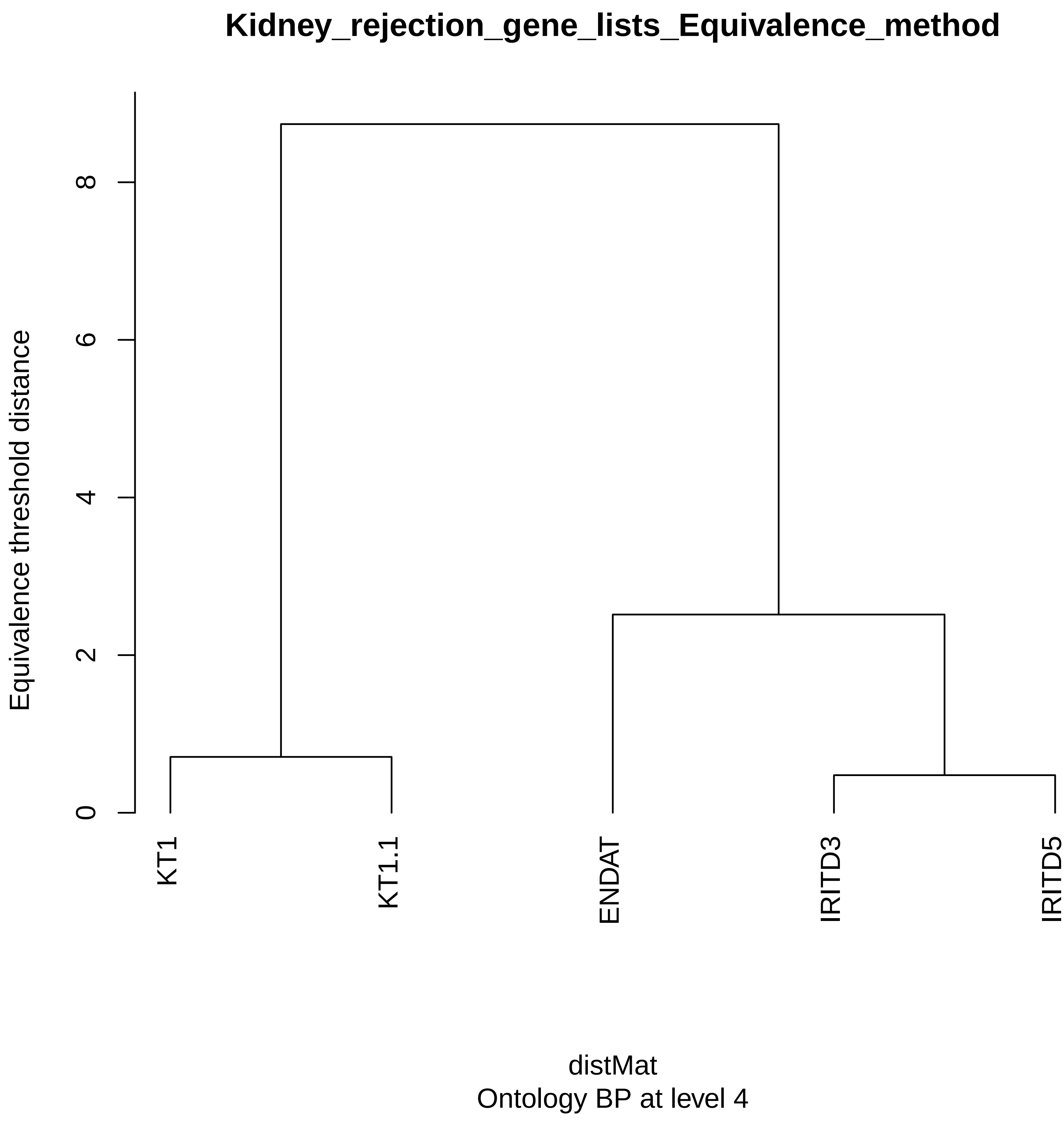
Kidney_rejection_gene_lists_Equivalence_method
Equivalence threshold distance
0
2
4
6
8
KT1
KT1.1
ENDAT
IRITD3
IRITD5
distMat
Ontology BP at level 4

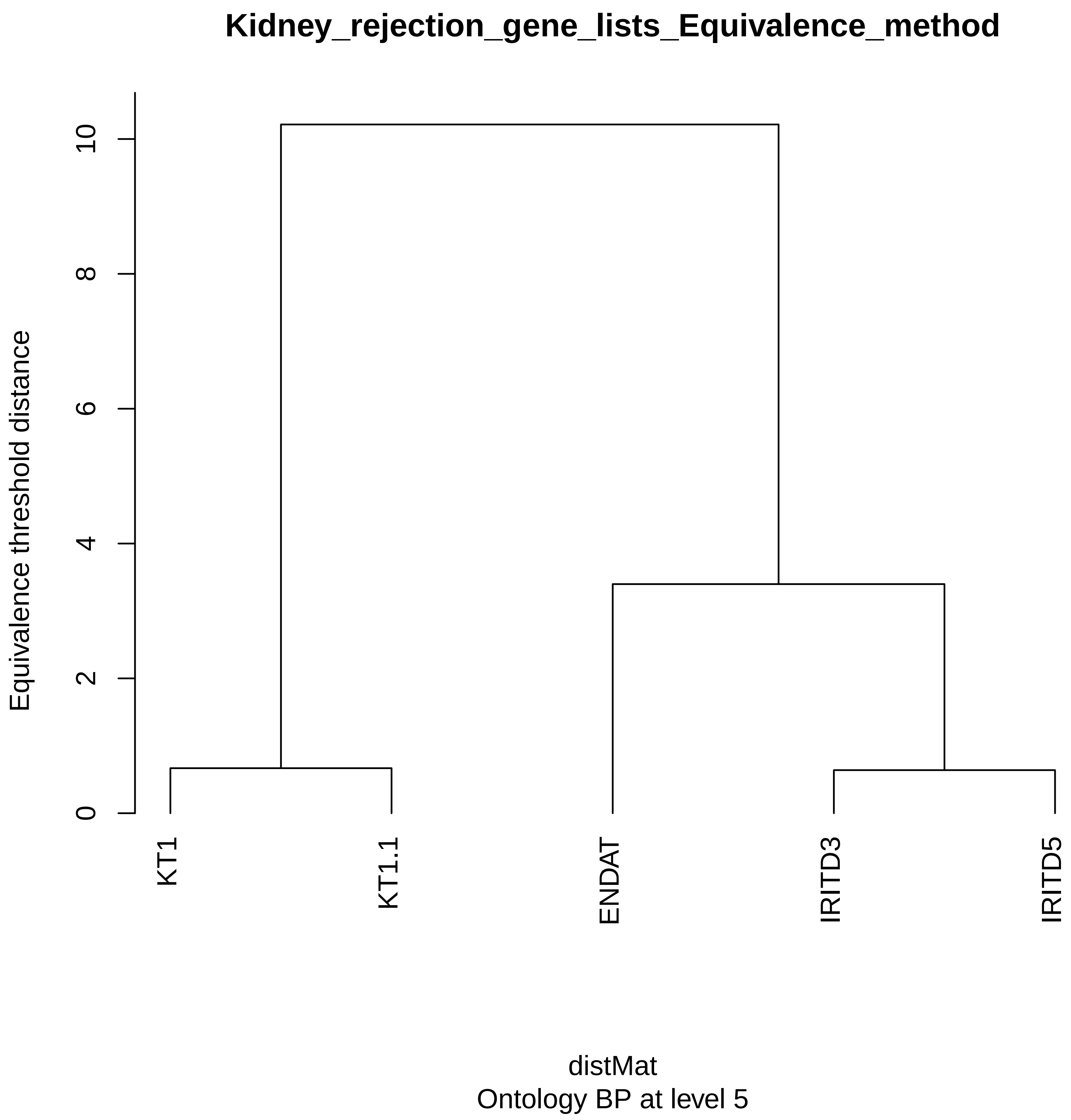

Kidney_rejection_gene_lists_Equivalence_method
Equivalence threshold distance
0
2
4
6
8
10
KT1
KT1.1
ENDAT
IRITD3
IRITD5
distMat
Ontology BP at level 5

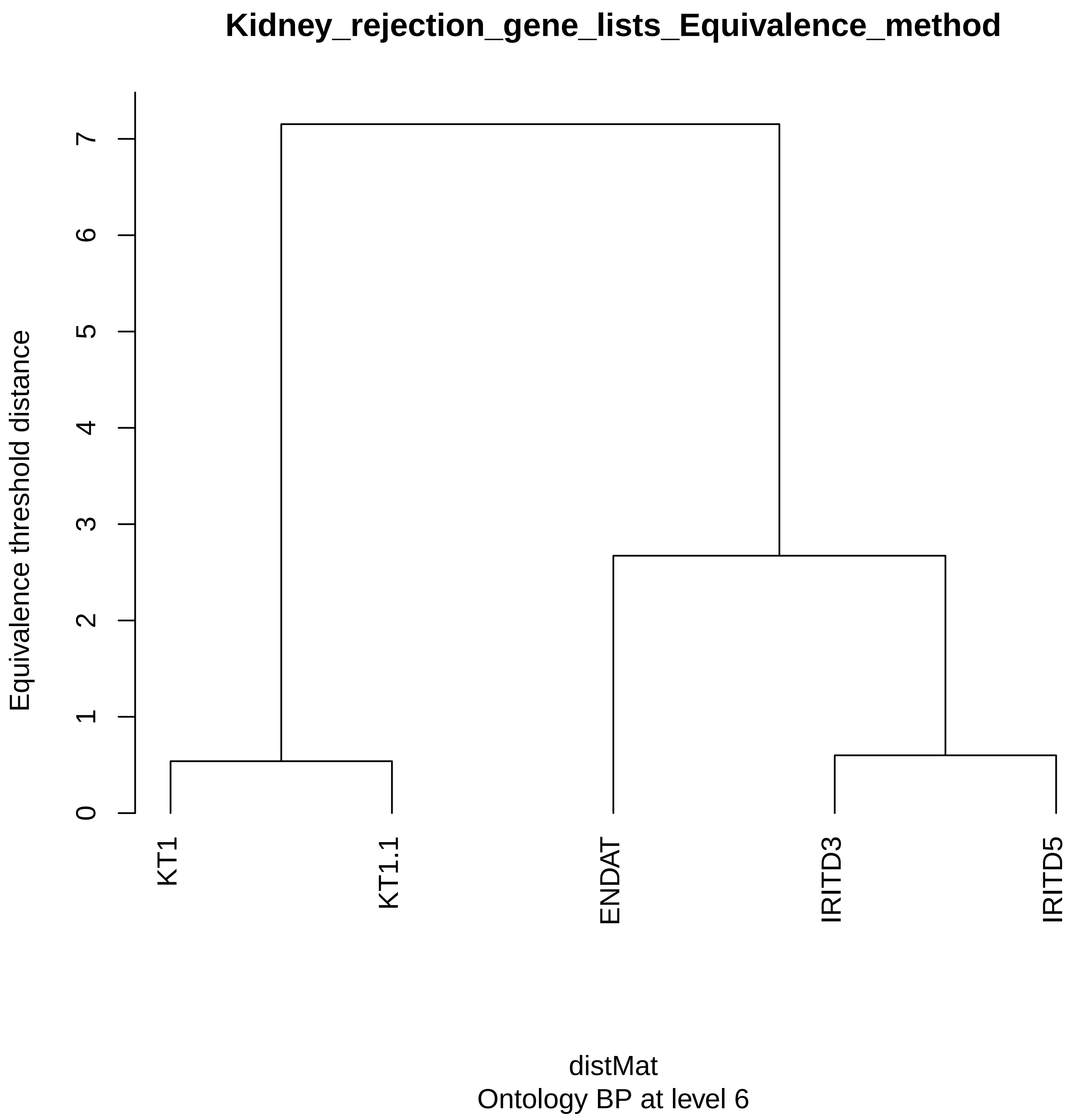
Kidney_rejection_gene_lists_Equivalence_method
Equivalence threshold distance
0
1
2
3
4
5
6
7
KT1
KT1.1
ENDAT
IRITD3
IRITD5
distMat
Ontology BP at level 6

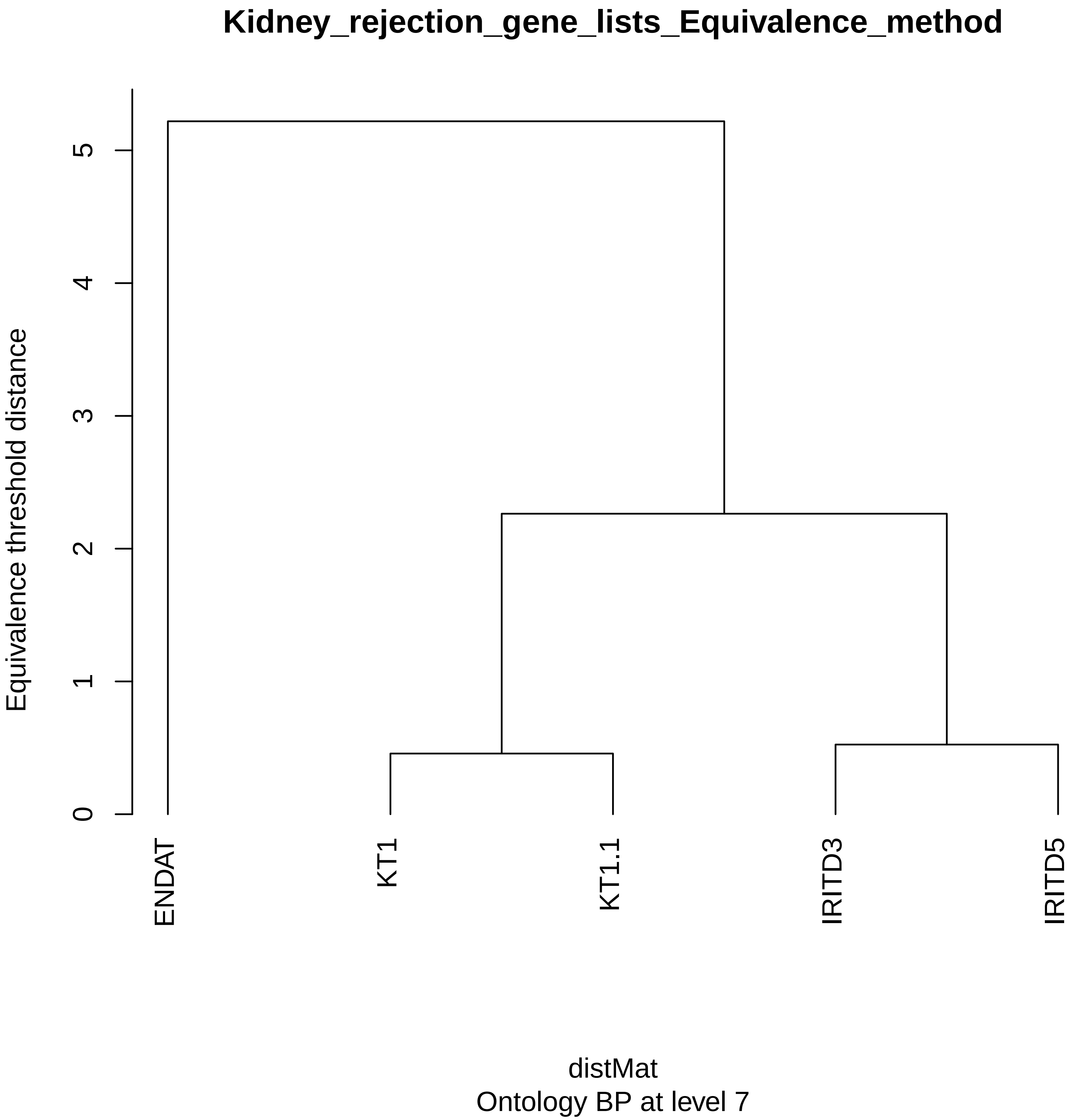
Kidney_rejection_gene_lists_Equivalence_method
Equivalence threshold distance
0
1
2
3
4
5
ENDAT
KT1
KT1.1
IRITD3
IRITD5
distMat
Ontology BP at level 7

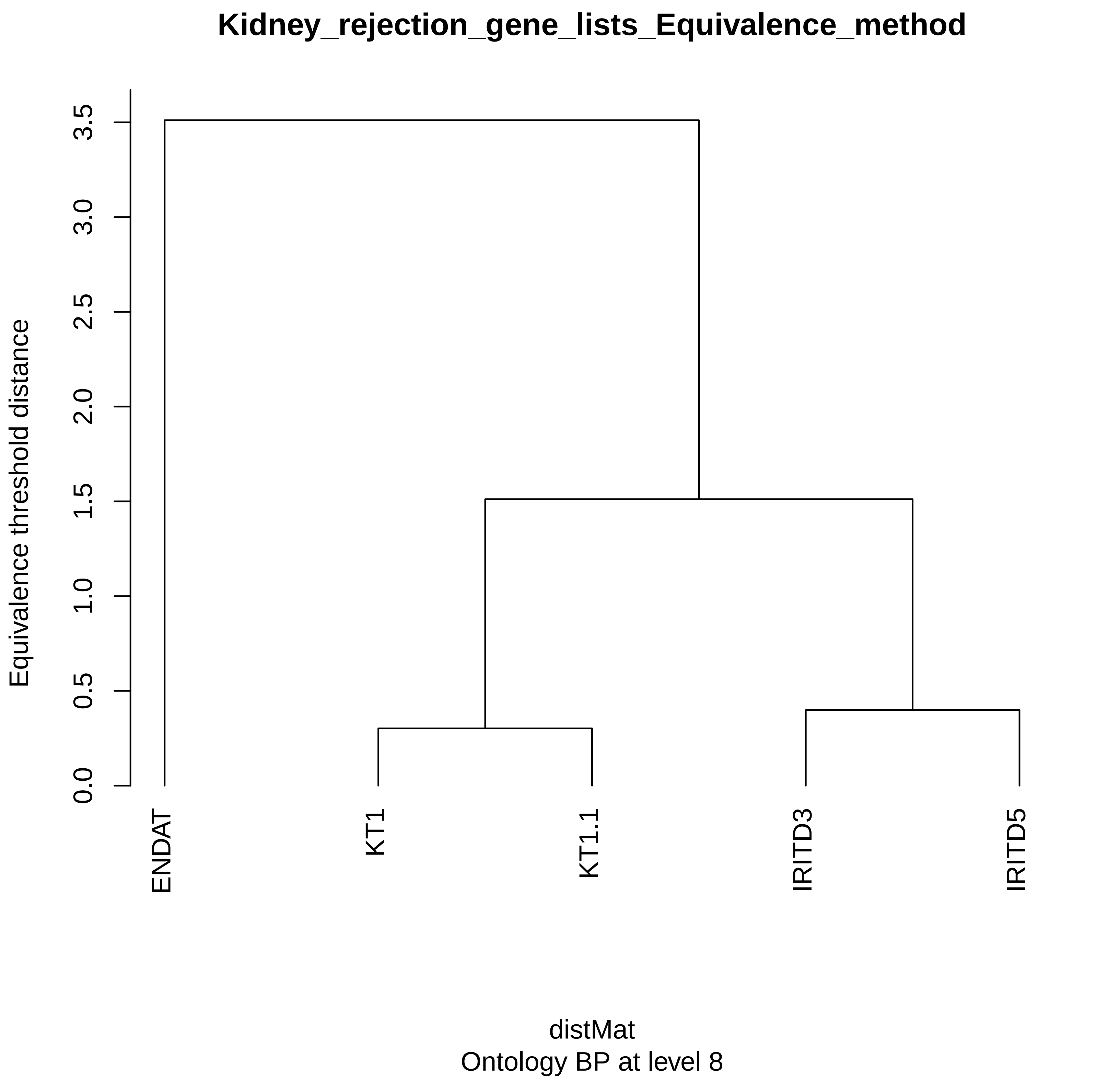
Kidney_rejection_gene_lists_Equivalence_method
Equivalence threshold distance
0.0
0.5
1.0
1.5
2.0
2.5
3.0
3.5
ENDAT
KT1
KT1.1
IRITD3
IRITD5
distMat
Ontology BP at level 8

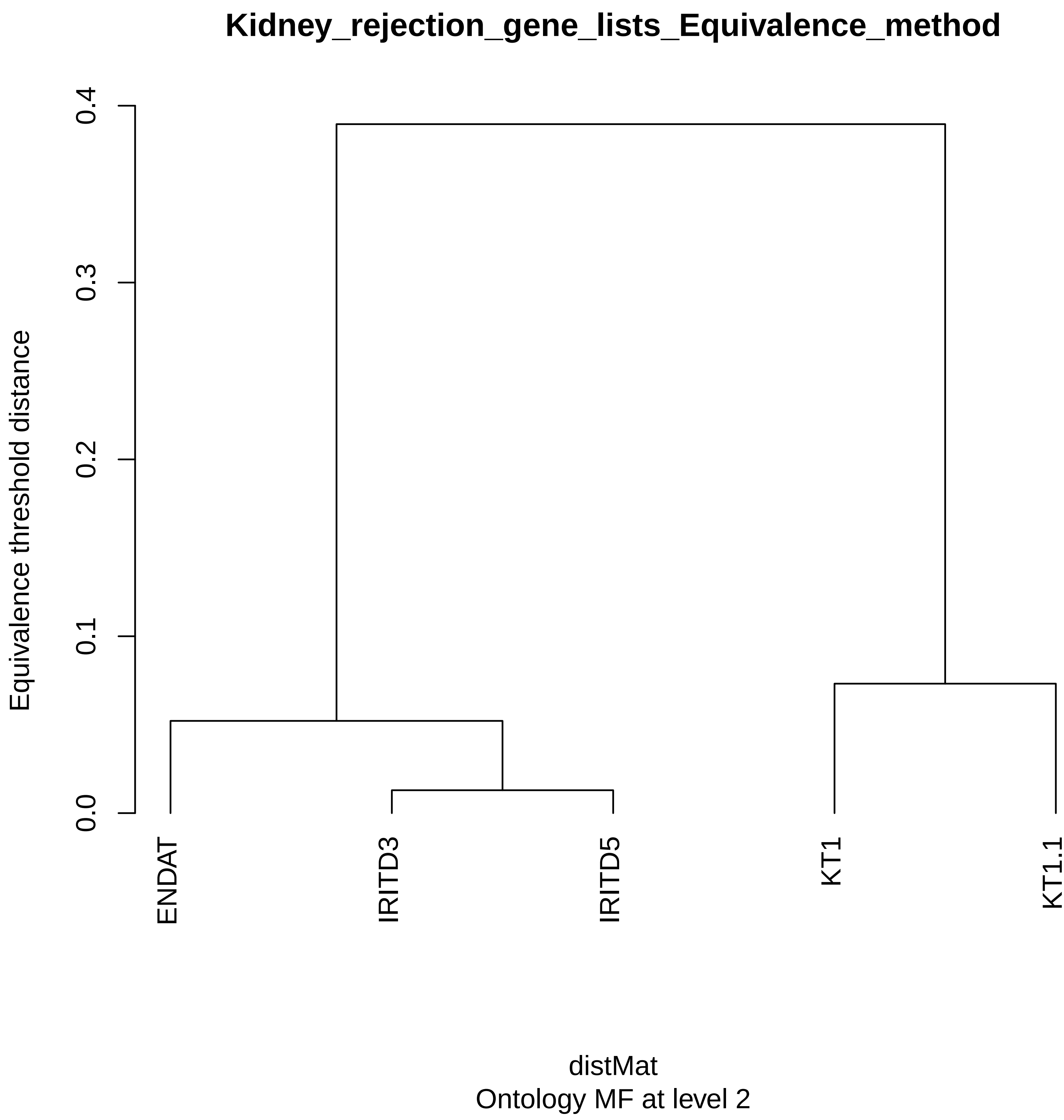

Kidney_rejection_gene_lists_Equivalence_method
Equivalence threshold distance
0.0
0.1
0.2
0.3
0.4
ENDAT
IRITD3
IRITD5
KT1
KT1.1
distMat
Ontology MF at level 2

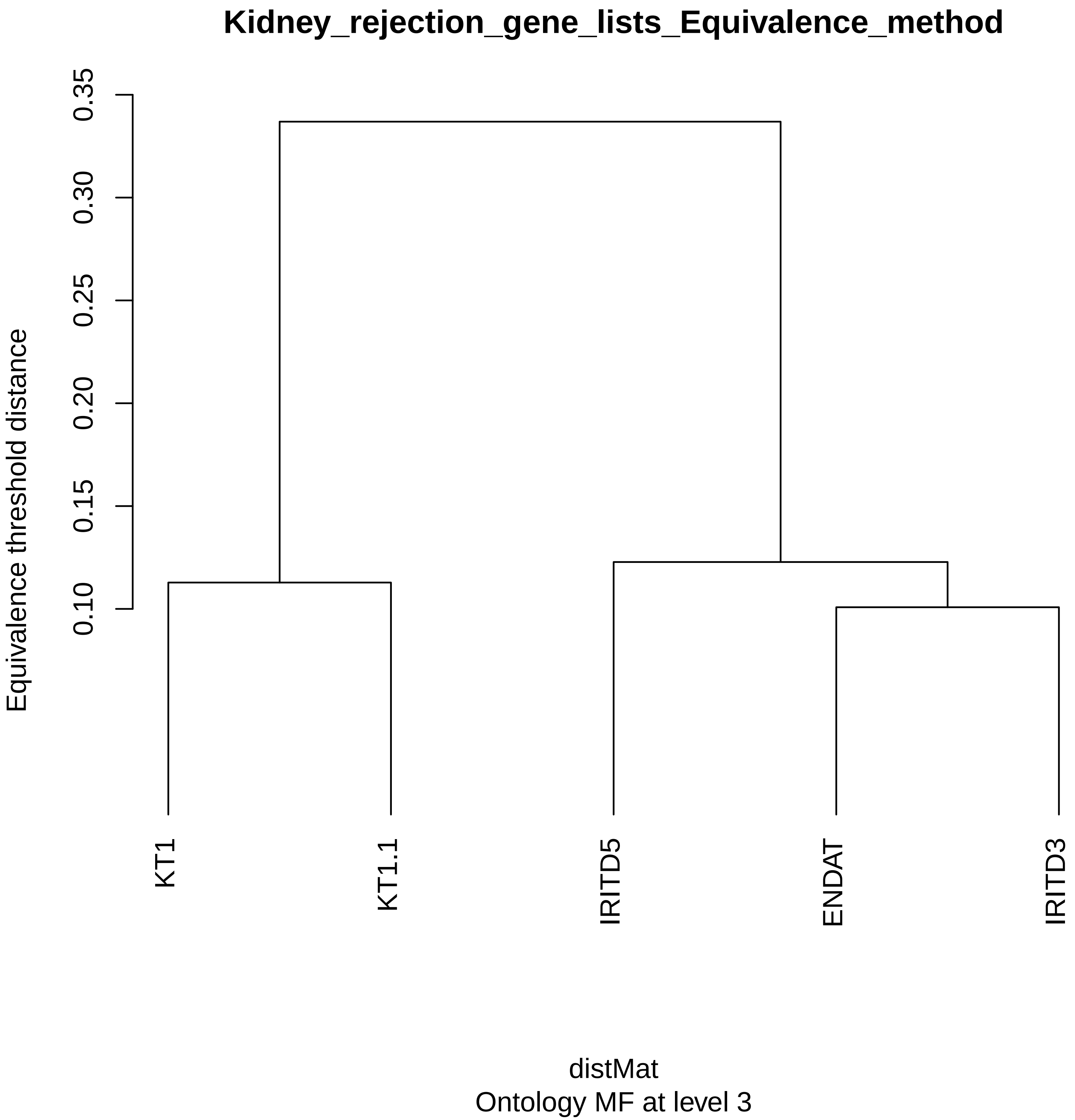
Kidney_rejection_gene_lists_Equivalence_method
Equivalence threshold distance
0.10
0.15
0.20
0.25
0.30
0.35
KT1
KT1.1
IRITD5
ENDAT
IRITD3
distMat
Ontology MF at level 3

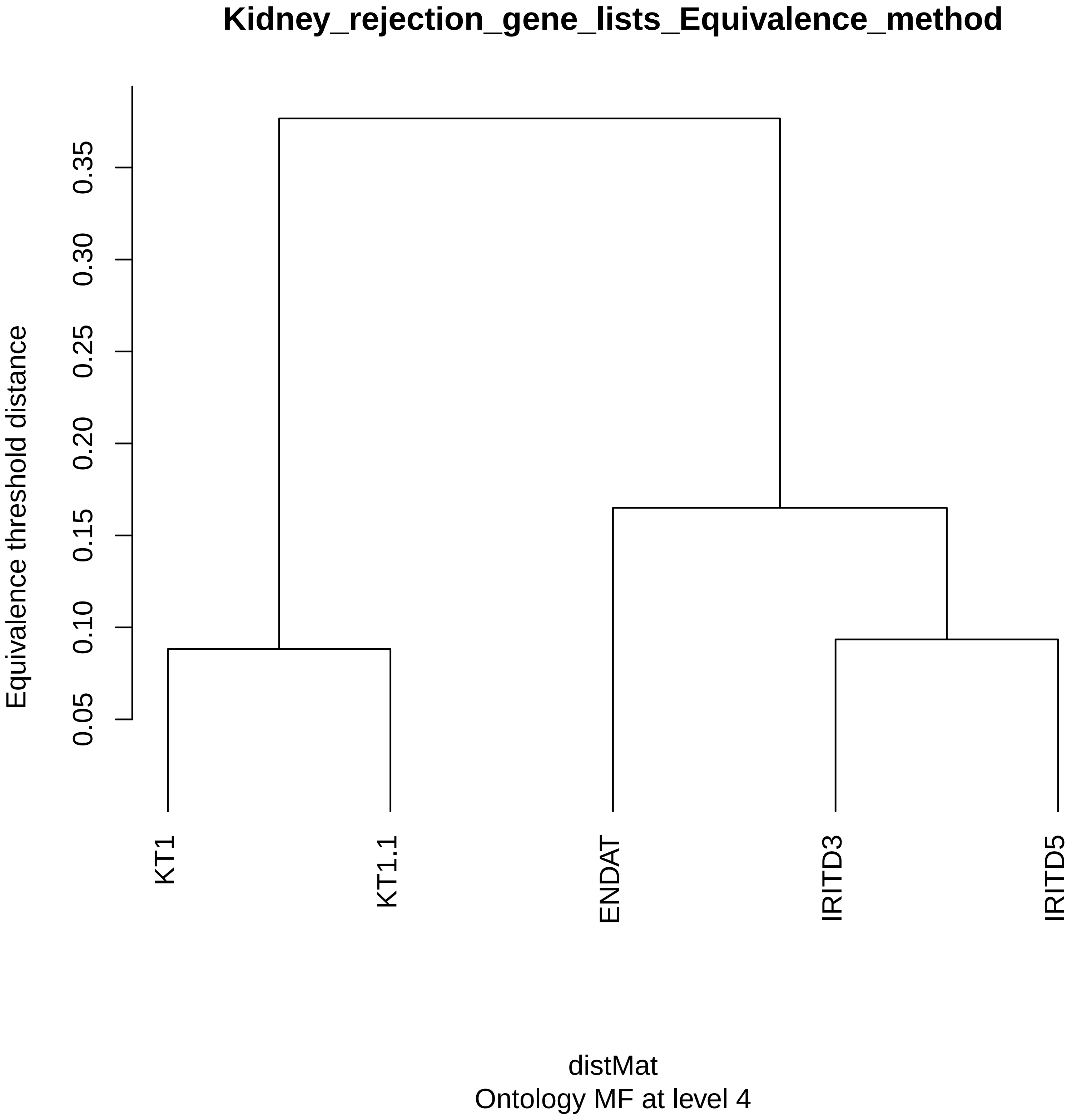
Kidney_rejection_gene_lists_Equivalence_method
Equivalence threshold distance
0.05
0.10
0.15
0.20
0.25
0.30
0.35
KT1
KT1.1
ENDAT
IRITD3
IRITD5
distMat
Ontology MF at level 4

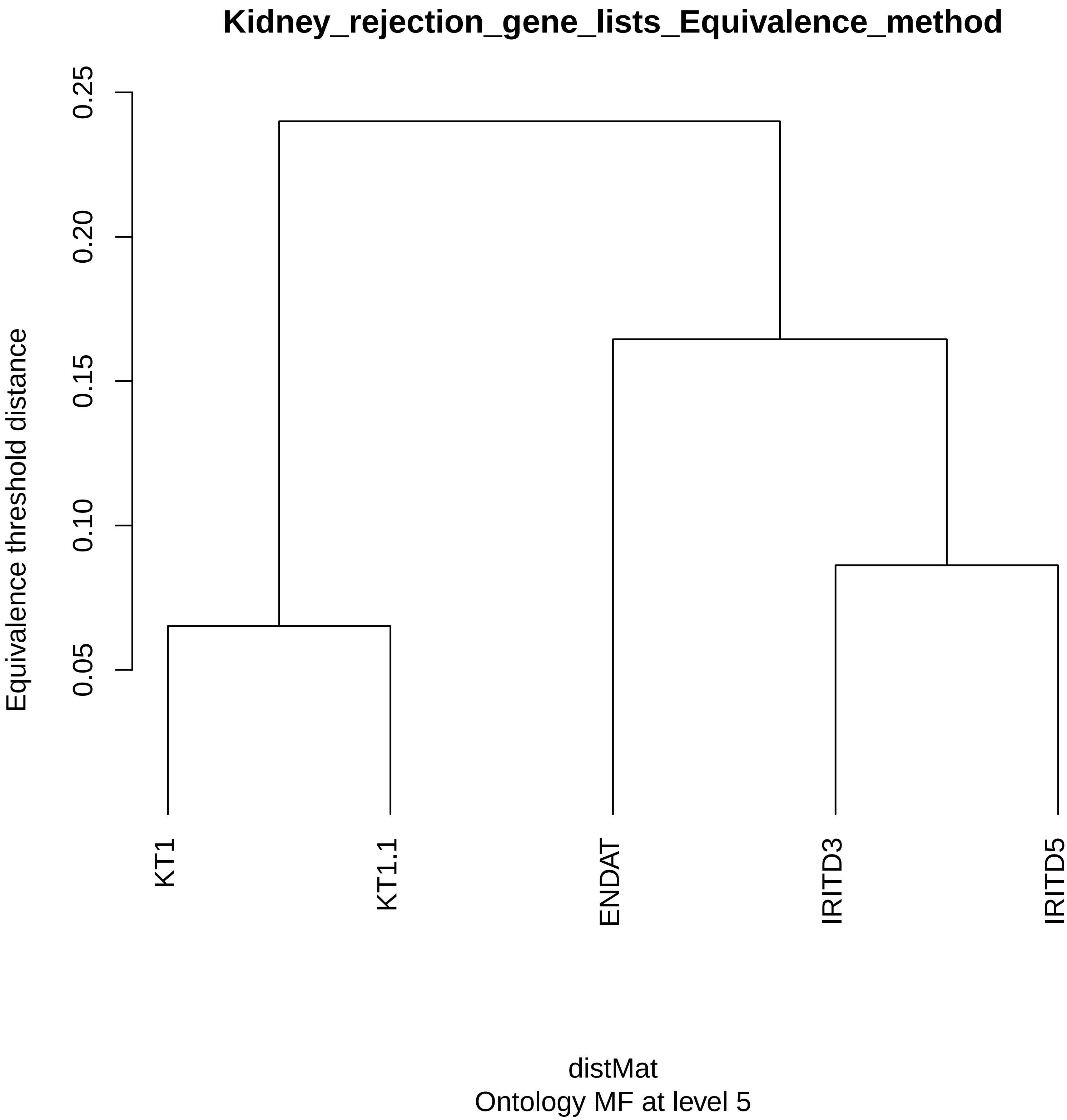

Kidney_rejection_gene_lists_Equivalence_method
Equivalence threshold distance
0.05
0.10
0.15
0.20
0.25
KT1
KT1.1
ENDAT
IRITD3
IRITD5
distMat
Ontology MF at level 5

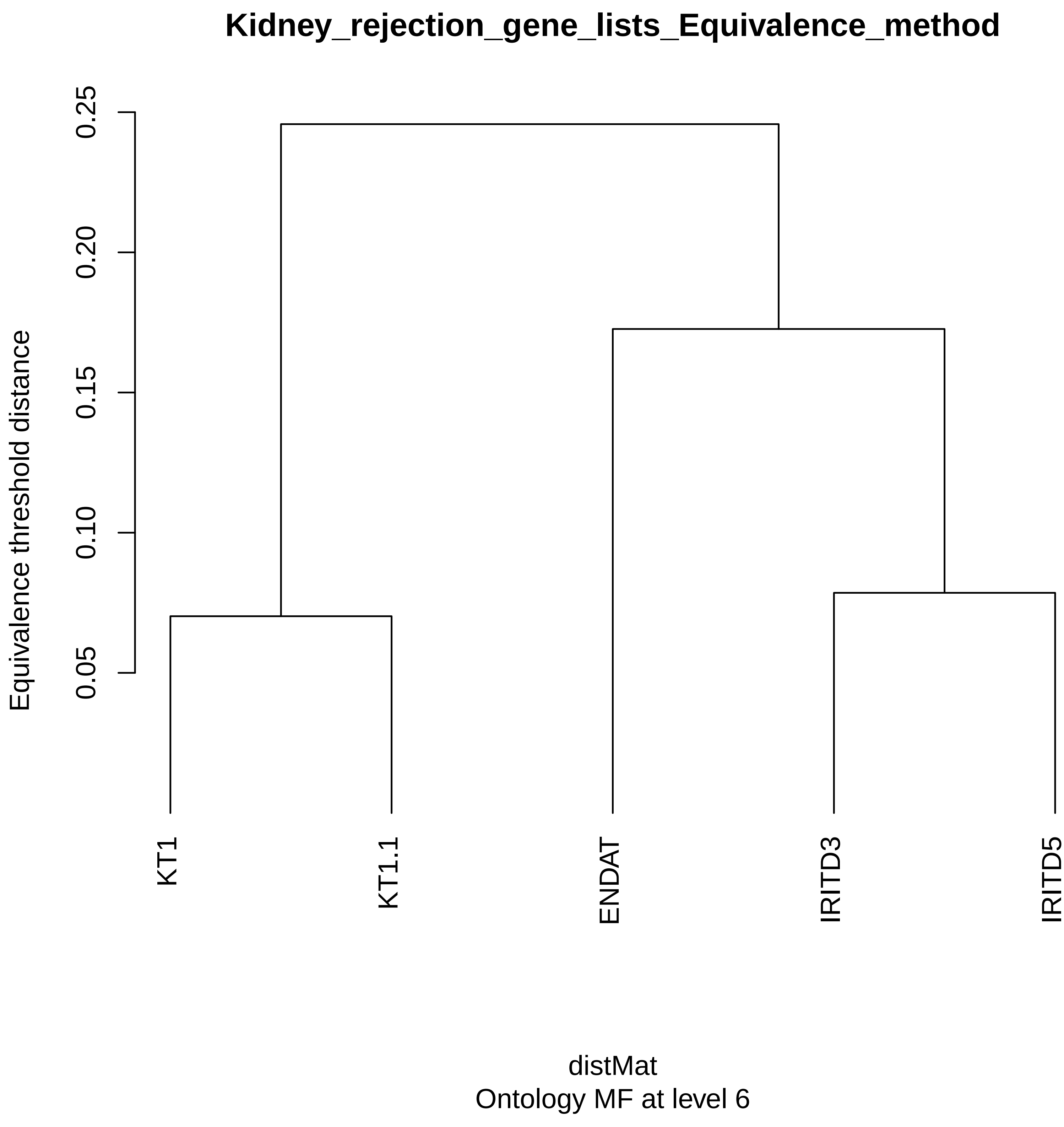

Kidney_rejection_gene_lists_Equivalence_method
Equivalence threshold distance
0.05
0.10
0.15
0.20
0.25
KT1
KT1.1
ENDAT
IRITD3
IRITD5
distMat
Ontology MF at level 6

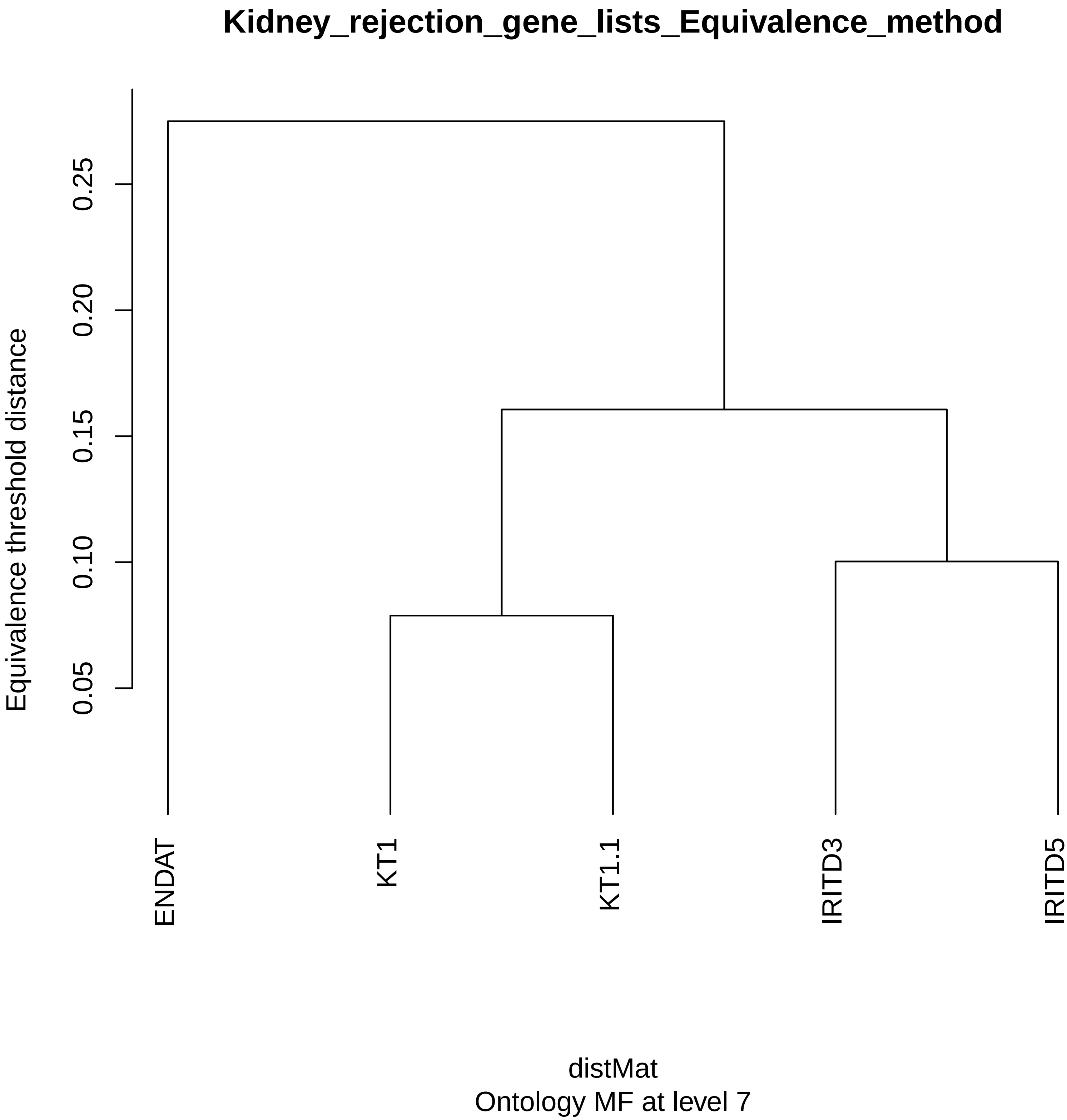
Kidney_rejection_gene_lists_Equivalence_method
Equivalence threshold distance
0.05
0.10
0.15
0.20
0.25
ENDAT
KT1
KT1.1
IRITD3
IRITD5
distMat
Ontology MF at level 7

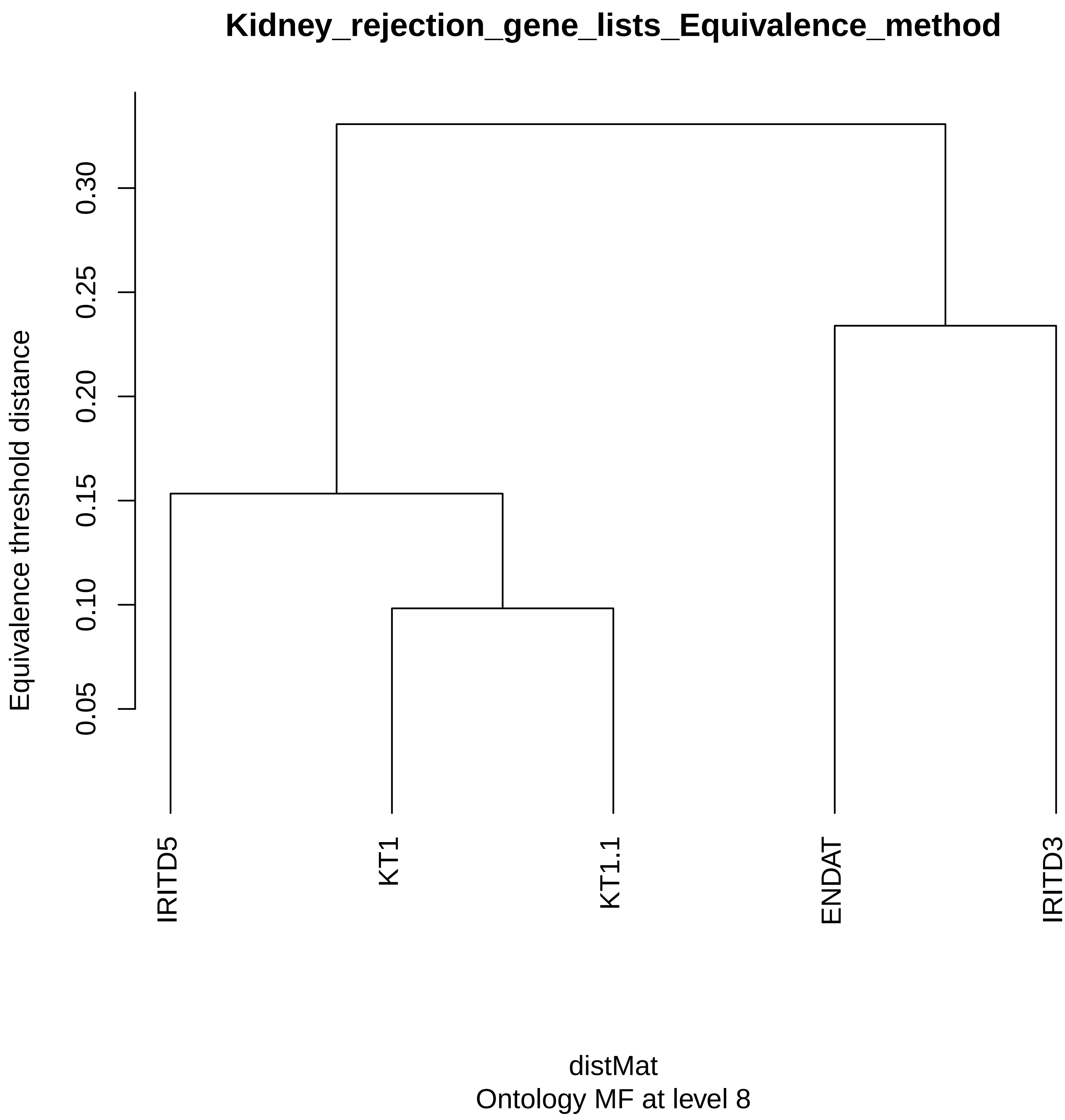
Kidney_rejection_gene_lists_Equivalence_method
Equivalence threshold distance
0.05
0.10
0.15
0.20
0.25
0.30
IRITD5
KT1
KT1.1
ENDAT
IRITD3
distMat
Ontology MF at level 8

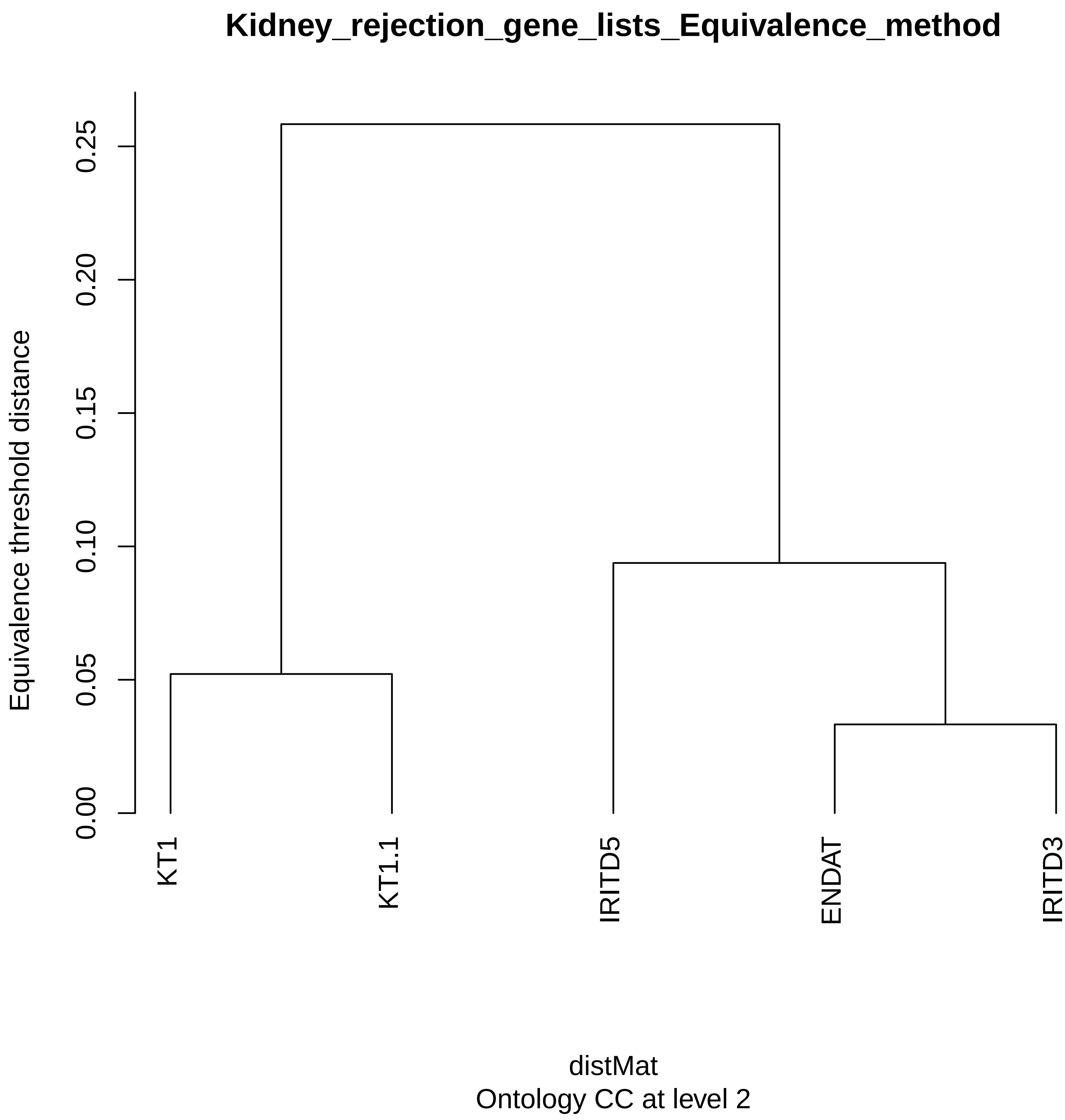
Kidney_rejection_gene_lists_Equivalence_method
Equivalence threshold distance
0.00
0.05
0.10
0.15
0.20
0.25
KT1
KT1.1
IRITD5
ENDAT
IRITD3
distMat
Ontology CC at level 2

Kidney_rejection_gene_lists_Equivalence_method

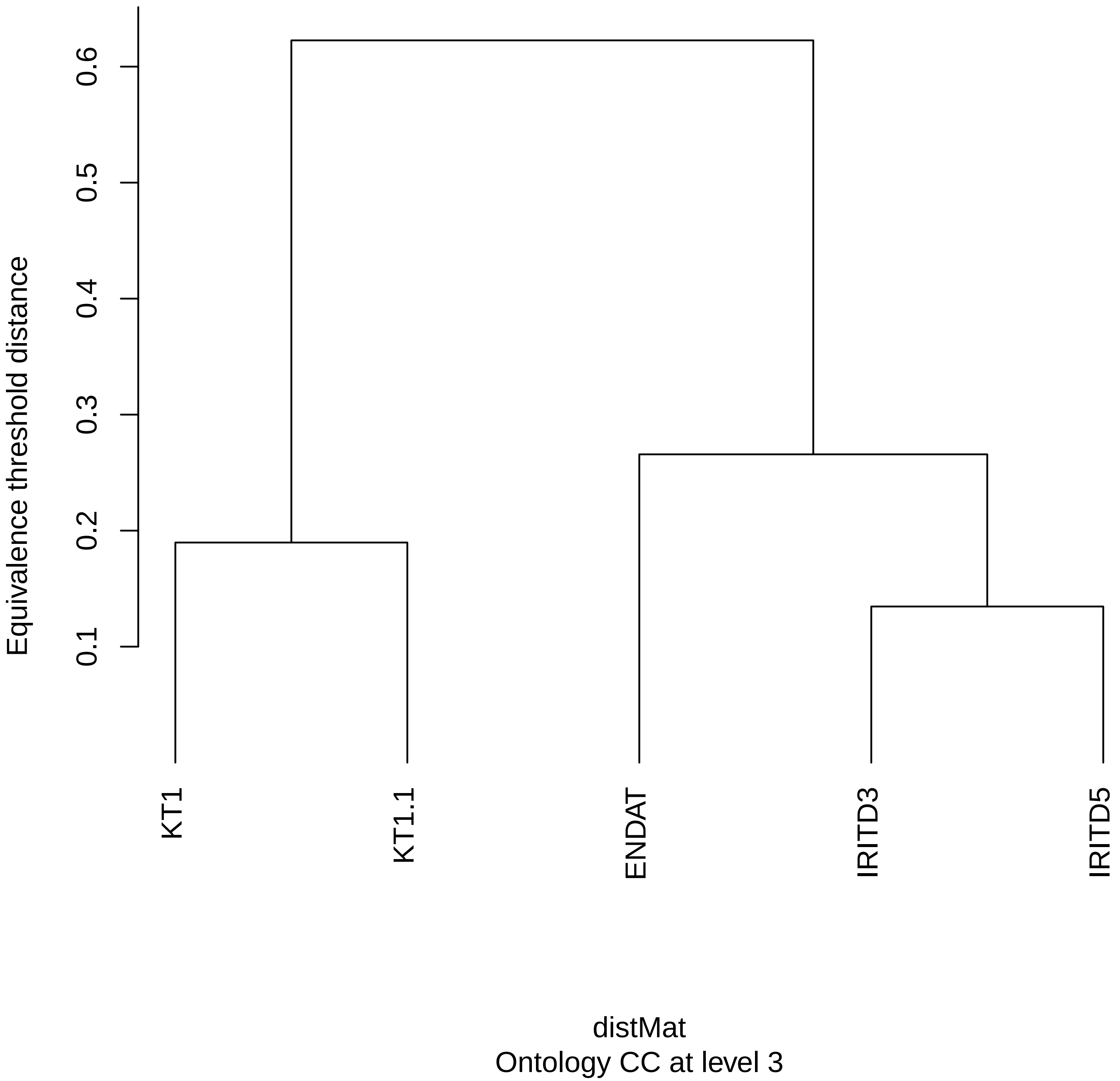

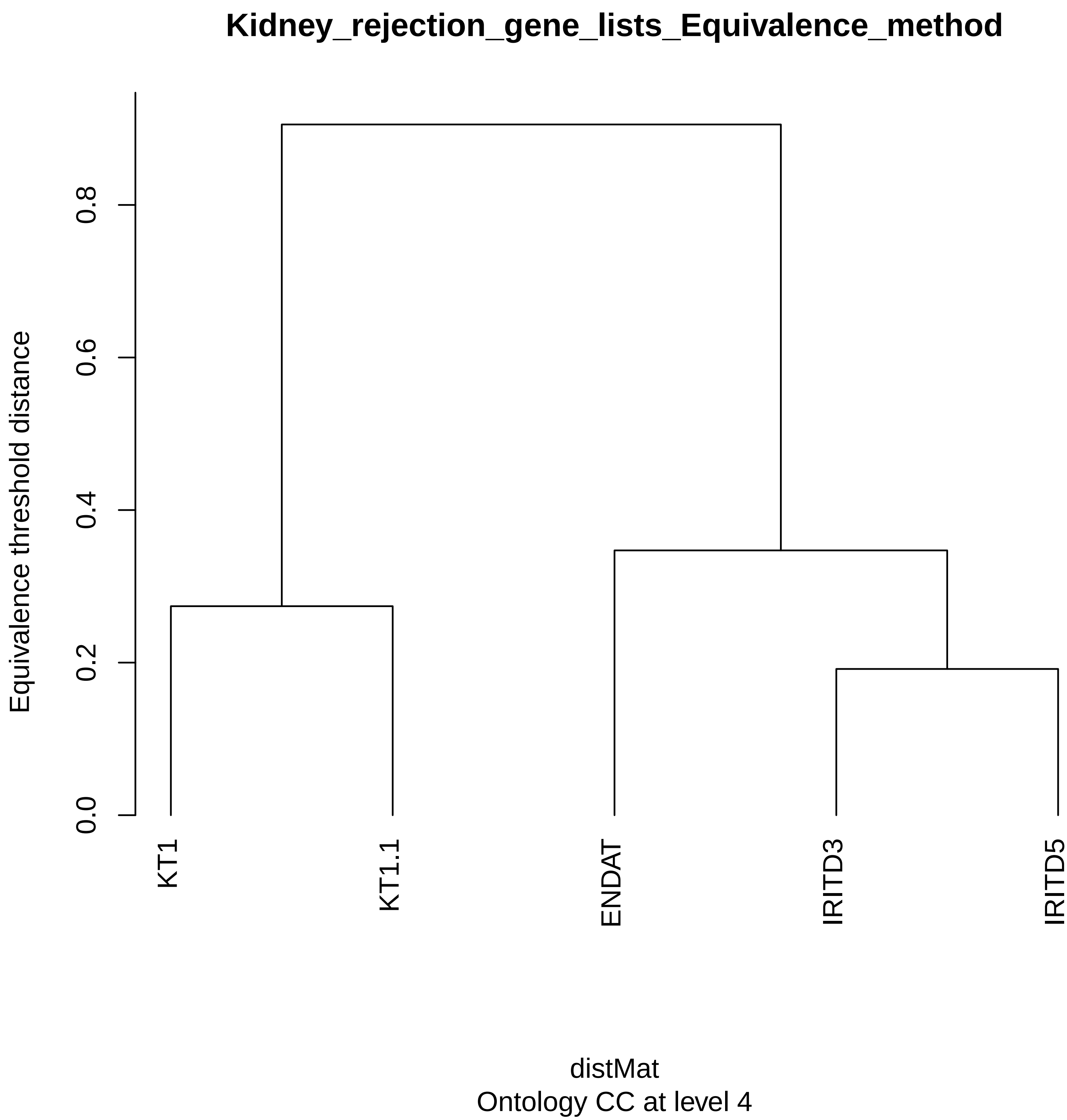
Kidney_rejection_gene_lists_Equivalence_method
Equivalence threshold distance
0.0
0.2
0.4
0.6
0.8
KT1
KT1.1
ENDAT
IRITD3
IRITD5
distMat
Ontology CC at level 4

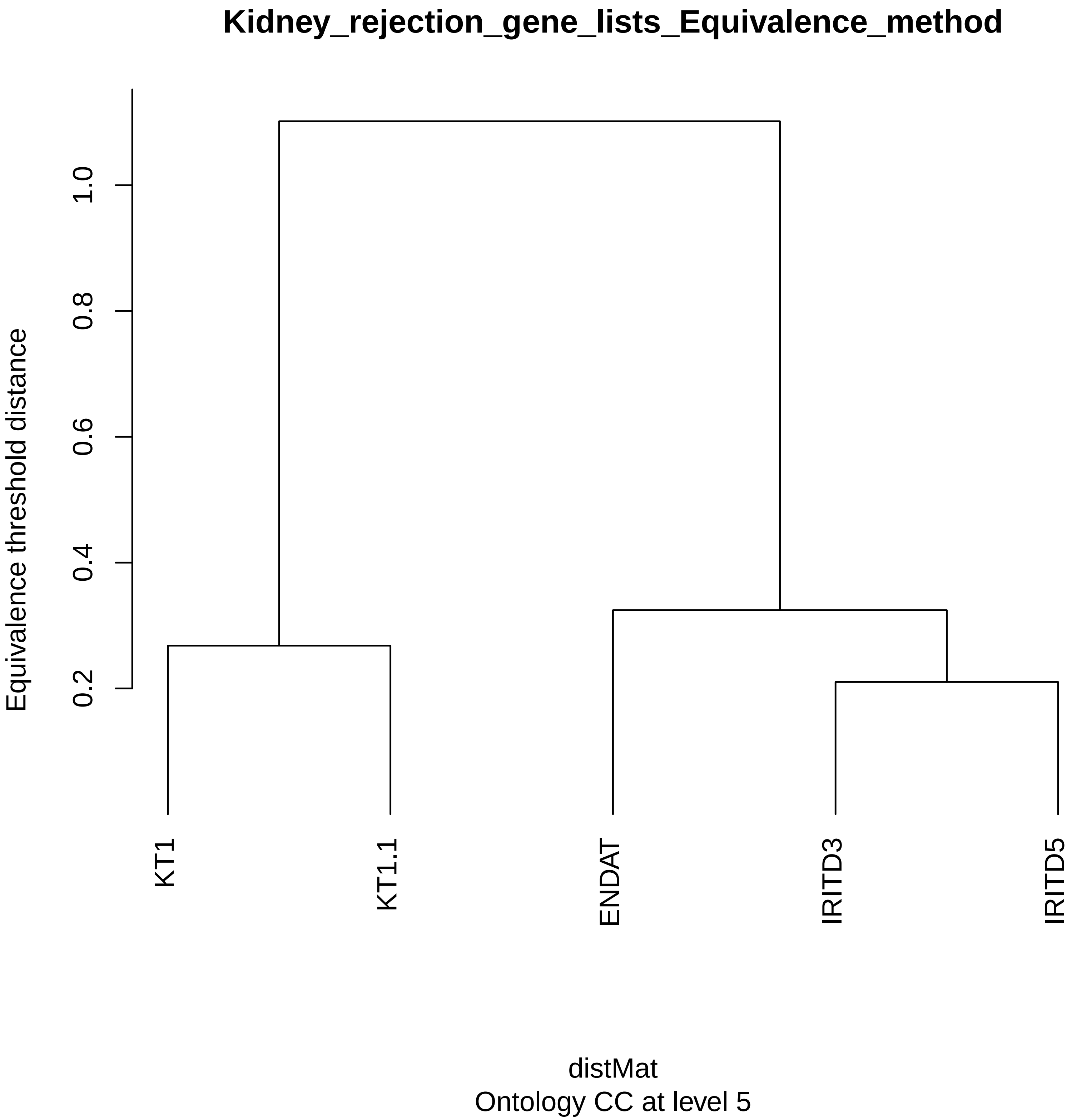

Kidney_rejection_gene_lists_Equivalence_method
Equivalence threshold distance
0.2
0.4
0.6
0.8
1.0
KT1
KT1.1
ENDAT
IRITD3
IRITD5
distMat
Ontology CC at level 5

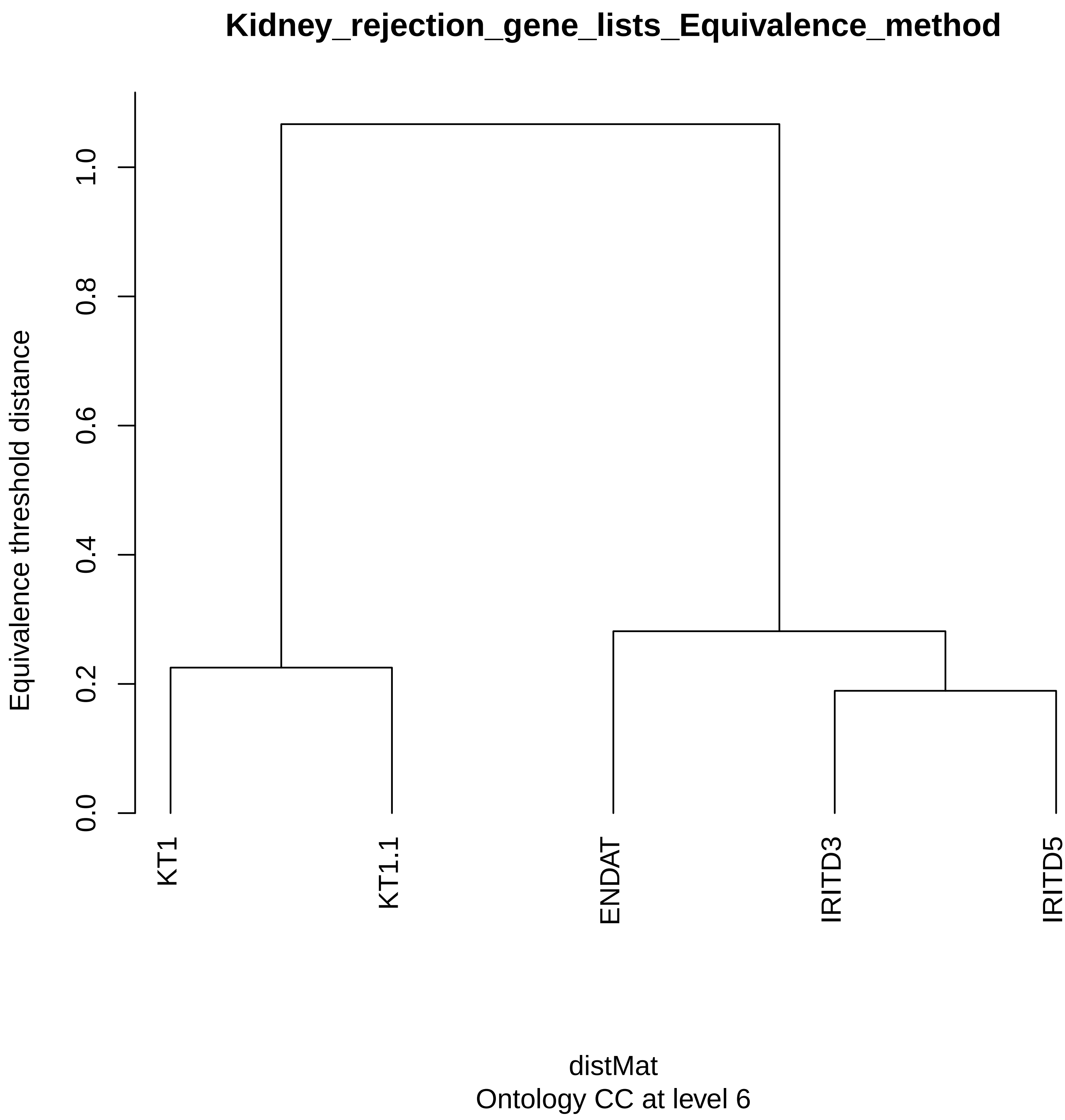
Kidney_rejection_gene_lists_Equivalence_method
Equivalence threshold distance
0.0
0.2
0.4
0.6
0.8
1.0
KT1
KT1.1
ENDAT
IRITD3
IRITD5
distMat
Ontology CC at level 6

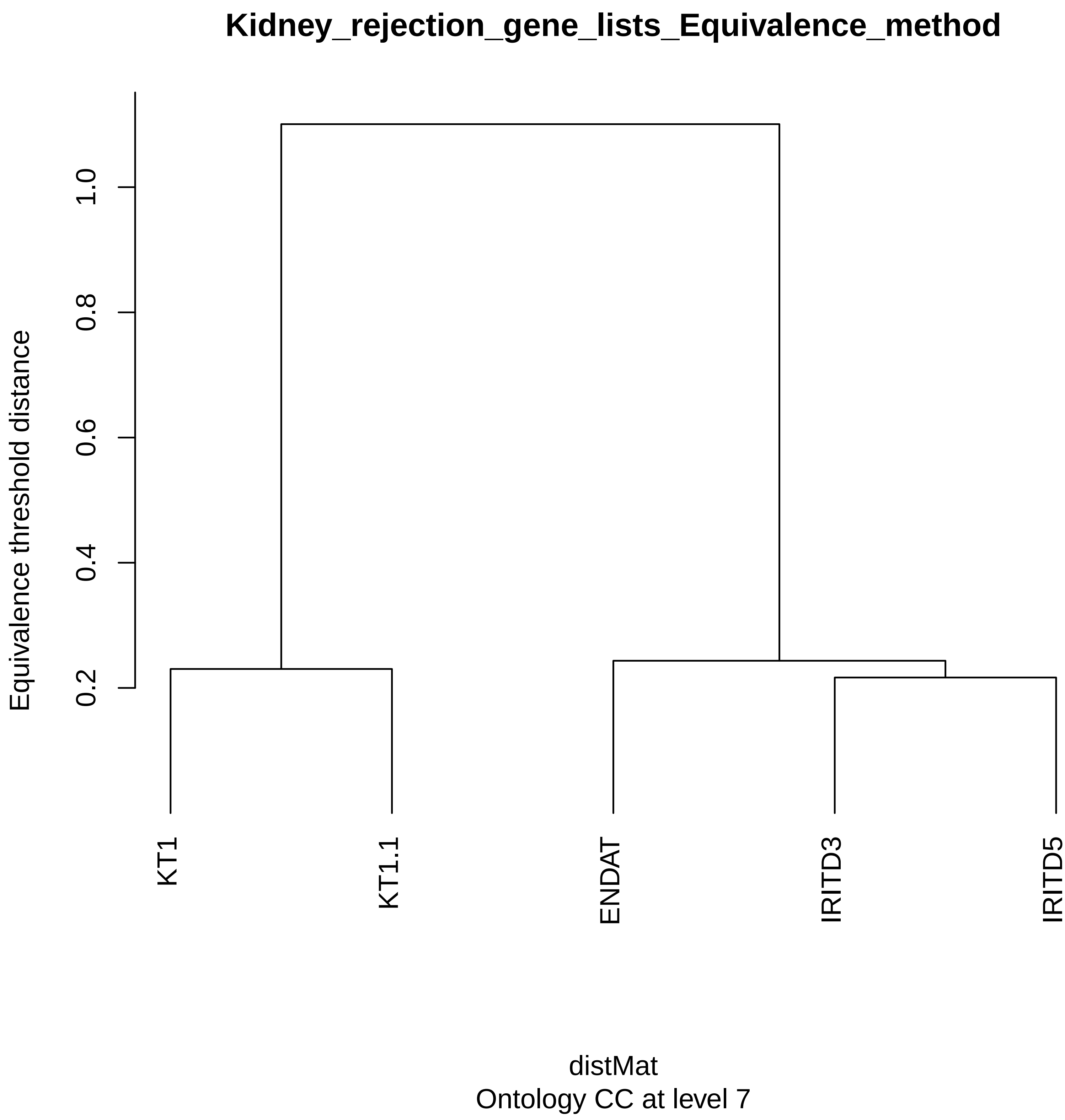

Kidney_rejection_gene_lists_Equivalence_method
Equivalence threshold distance
0.2
0.4
0.6
0.8
1.0
KT1
KT1.1
ENDAT
IRITD3
IRITD5
distMat
Ontology CC at level 7

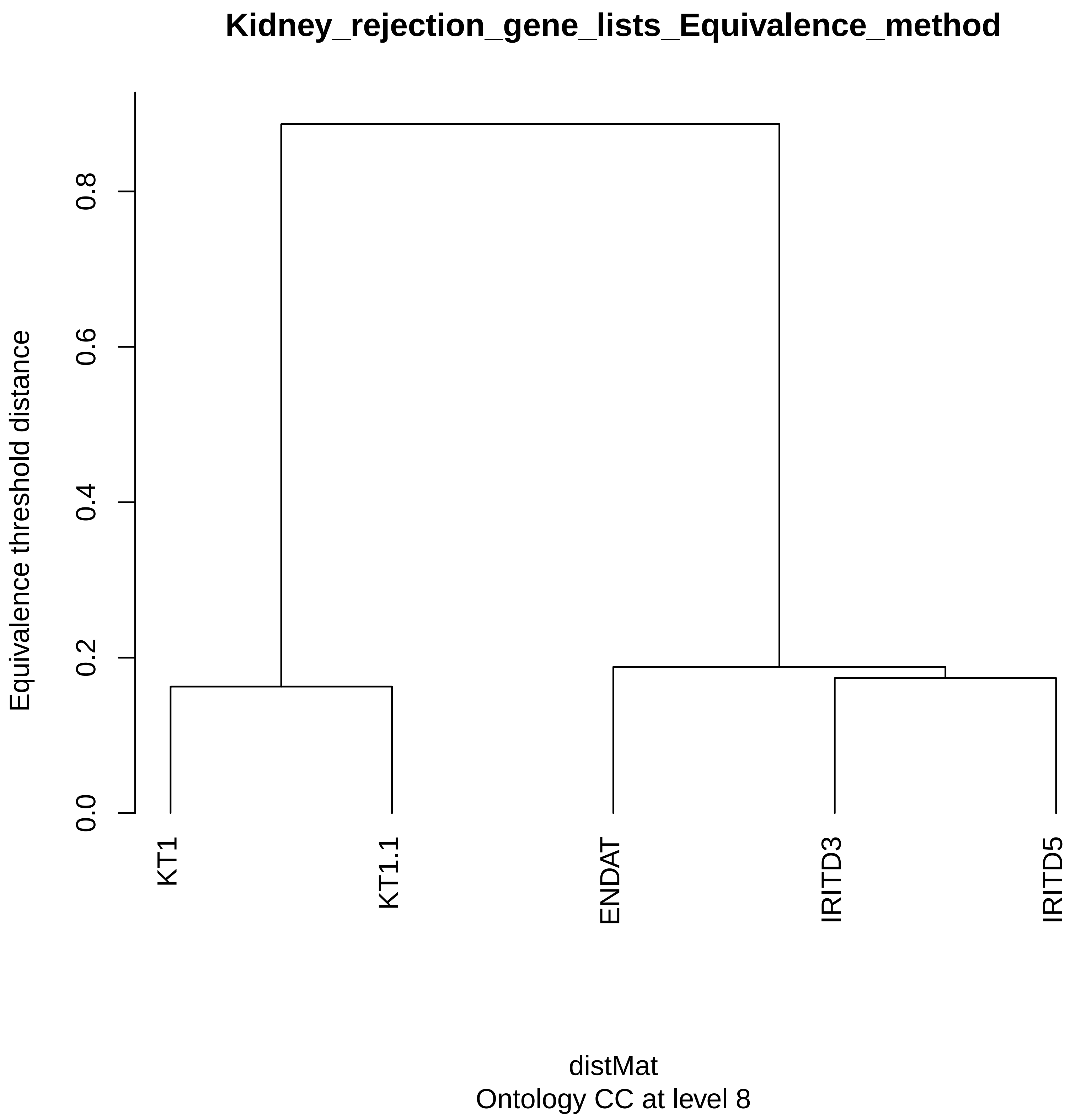
Kidney_rejection_gene_lists_Equivalence_method
Equivalence threshold distance
0.0
0.2
0.4
0.6
0.8
KT1
KT1.1
ENDAT
IRITD3
IRITD5
distMat
Ontology CC at level 8

KIDNEY TRANSPLANTATION REJECTION LISTS
Number of GO annotated genes for each list, ontology and GO level

GO level 2

	ENDAT	IRITD3	IRITD5	KT1	KT1.1
MF	108	290	195	515	112
BP	112	285	200	506	113
CC	114	302	204	536	117

GO level 3

	ENDAT	IRITD3	IRITD5	KT1	KT1.1
MF	106	280	187	504	110
BP	112	283	200	508	113
CC	113	299	204	533	116

GO level 4

	ENDAT	IRITD3	IRITD5	KT1	KT1.1
MF	101	265	185	482	108
BP	112	284	198	495	108
CC	113	299	204	533	116

GO level 5

	ENDAT	IRITD3	IRITD5	KT1	KT1.1
MF	100	248	179	463	106
BP	111	283	199	502	112
CC	113	298	204	527	116

GO level 6

	ENDAT	IRITD3	IRITD5	KT1	KT1.1
MF	87	207	144	424	99
BP	112	282	196	498	110
CC	107	287	192	498	111

GO level 7

	ENDAT	IRITD3	IRITD5	KT1	KT1.1
MF	57	149	93	296	71
BP	108	271	190	484	106
CC	99	274	172	459	103

GO level 8

	ENDAT	IRITD3	IRITD5	KT1	KT1.1
MF	45	96	58	190	37
BP	107	261	184	461	104
CC	98	270	171	457	102

GO level 9

	ENDAT	IRITD3	IRITD5	KT1	KT1.1
MF	36	74	45	142	23
BP	99	249	178	424	96
CC	79	199	142	308	67

GO level 10

	ENDAT	IRITD3	IRITD5	KT1	KT1.1
MF	15	25	18	59	9
BP	92	231	160	371	81
CC	74	186	138	281	66

GO level 11

	ENDAT	IRITD3	IRITD5	KT1	KT1.1
MF	2	10	3	28	3
BP	86	196	133	312	66
CC	61	158	110	204	46

GO level 12

	ENDAT	IRITD3	IRITD5	KT1	KT1.1
MF	0	3	0	15	2
BP	77	165	113	228	38
CC	37	89	71	98	18

GO level 13

	ENDAT	IRITD3	IRITD5	KT1	KT1.1
MF	0	1	0	5	0
BP	68	136	88	152	27
CC	14	37	33	29	4

GO level 14

	ENDAT	IRITD3	IRITD5	KT1	KT1.1
MF	0	1	0	4	0
BP	36	67	51	70	14
CC	7	10	11	10	0

GO level 15

	ENDAT	IRITD3	IRITD5	KT1	KT1.1
MF	0	0	0	4	0
BP	17	30	33	38	7
CC	1	2	2	2	0

GO level 16

	ENDAT	IRITD3	IRITD5	KT1	KT1.1
MF	0	0	0	0	0
BP	5	12	11	15	3
CC	1	1	0	0	0

Semantic similarity methods are also a very interesting approach to analyzing the possible similarity between lists of genes. Despite many coincidences in the pattern of grouping, there is also considerable variability among them. These differences are comparable to those among GO levels in the equivalence method. Here we display the resulting dendrograms (complete method as before) for all semantic similarity methods implemented in R package GOSemSim and for all three GO ontologies.

Kidney rejection gene lists. Wang method, BP ontology

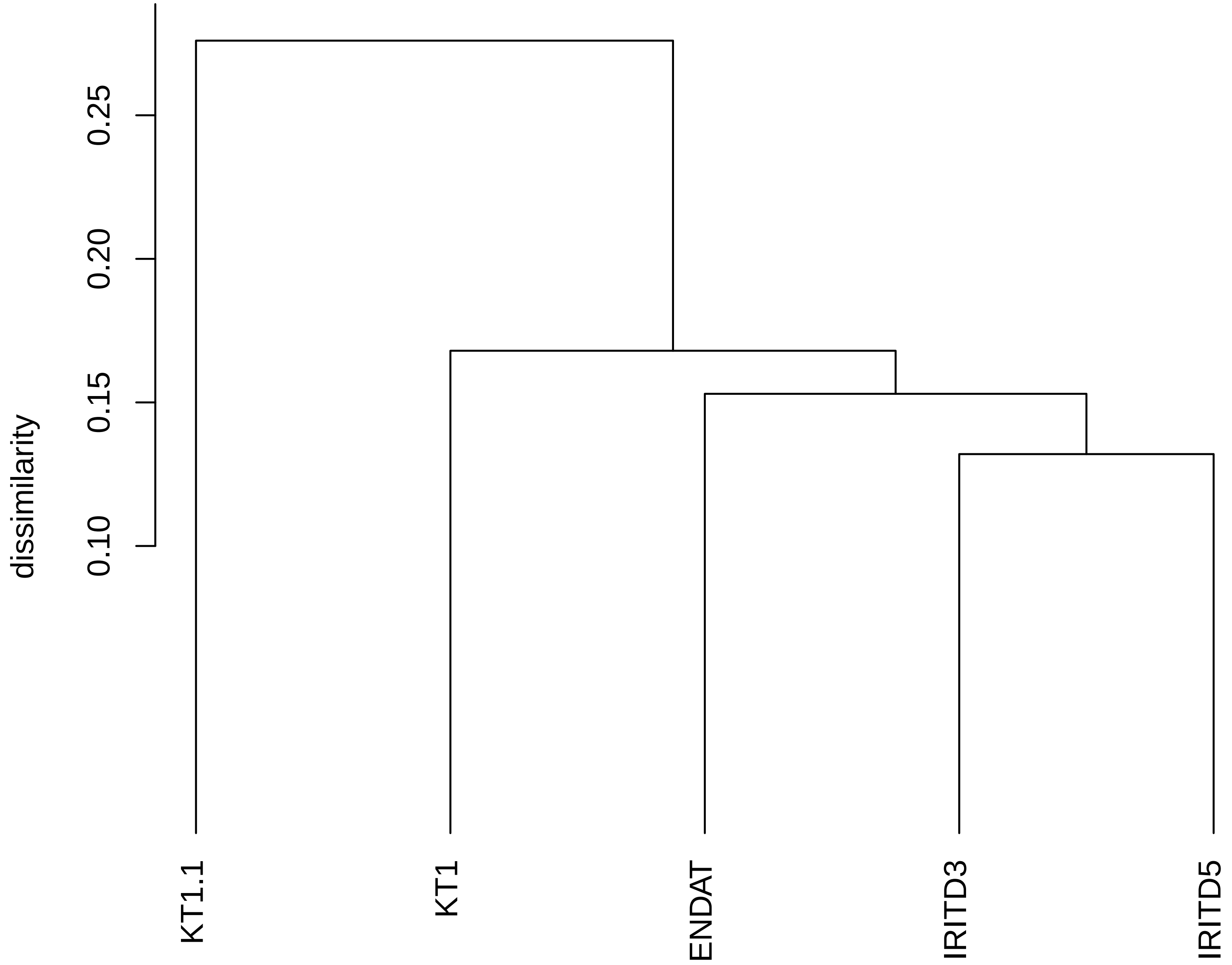

Dendrogram for Wang semantic similarity
(method = complete)

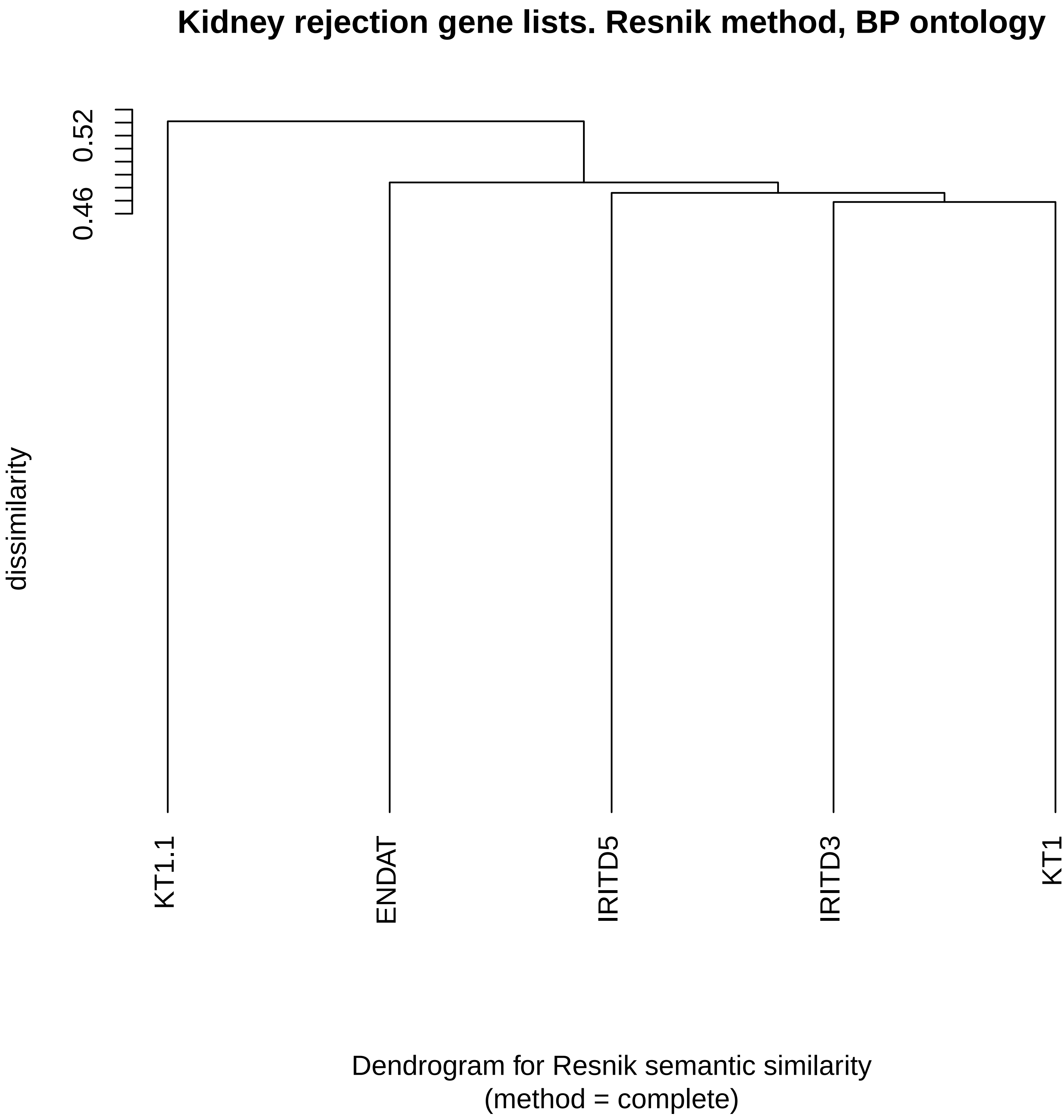
Kidney rejection gene lists. Resnik method, BP ontology
0.46
0.52
dissimilarity
KT1.1
ENDAT
IRITD5
IRITD3
KT1
Dendrogram for Resnik semantic similarity
(method = complete)

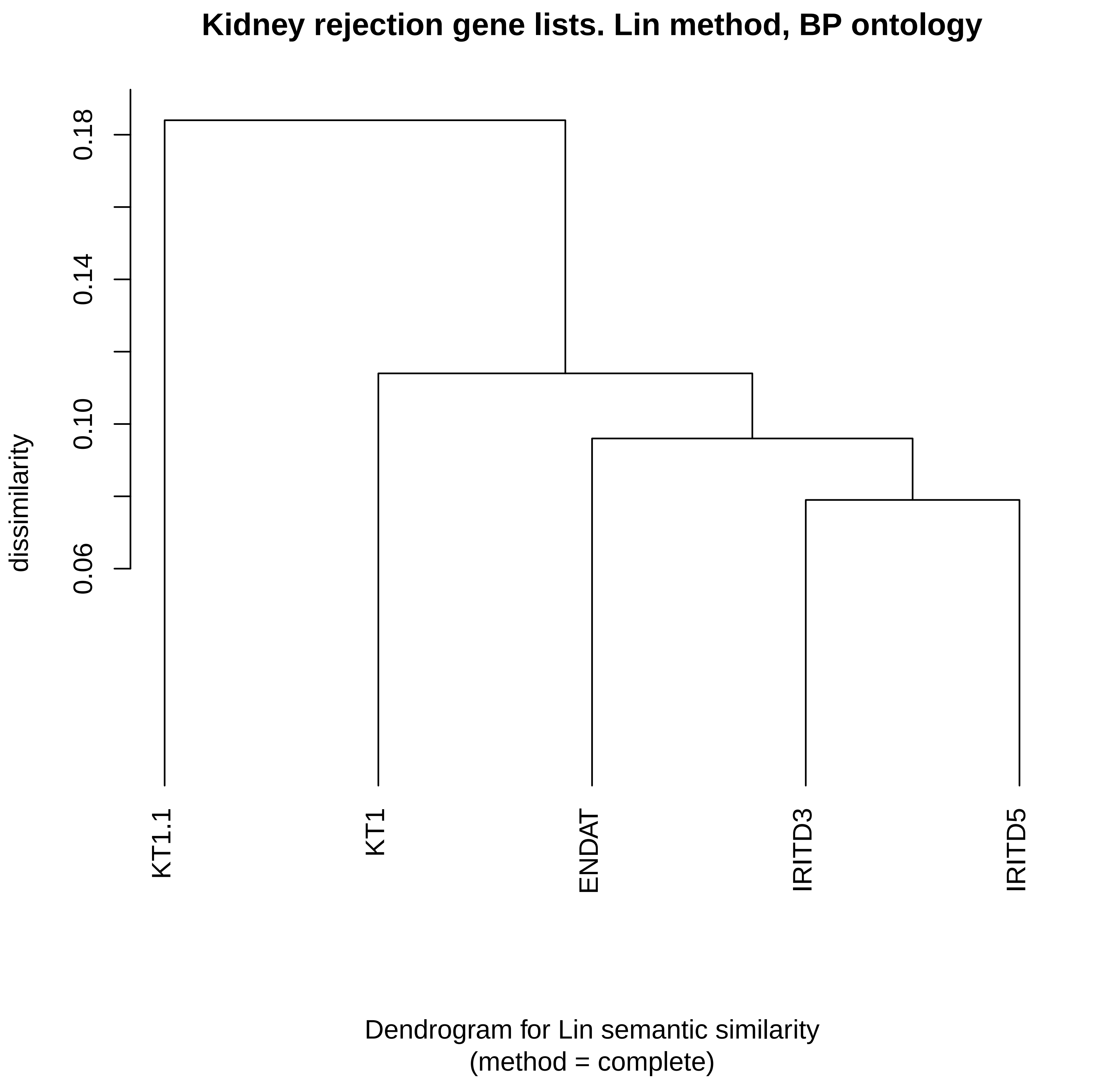

Kidney rejection gene lists. Lin method, BP ontology
dissimilarity
0.06
0.10
0.14
0.18
KT1.1
KT1
ENDAT
IRITD3
IRITD5
Dendrogram for Lin semantic similarity
(method = complete)

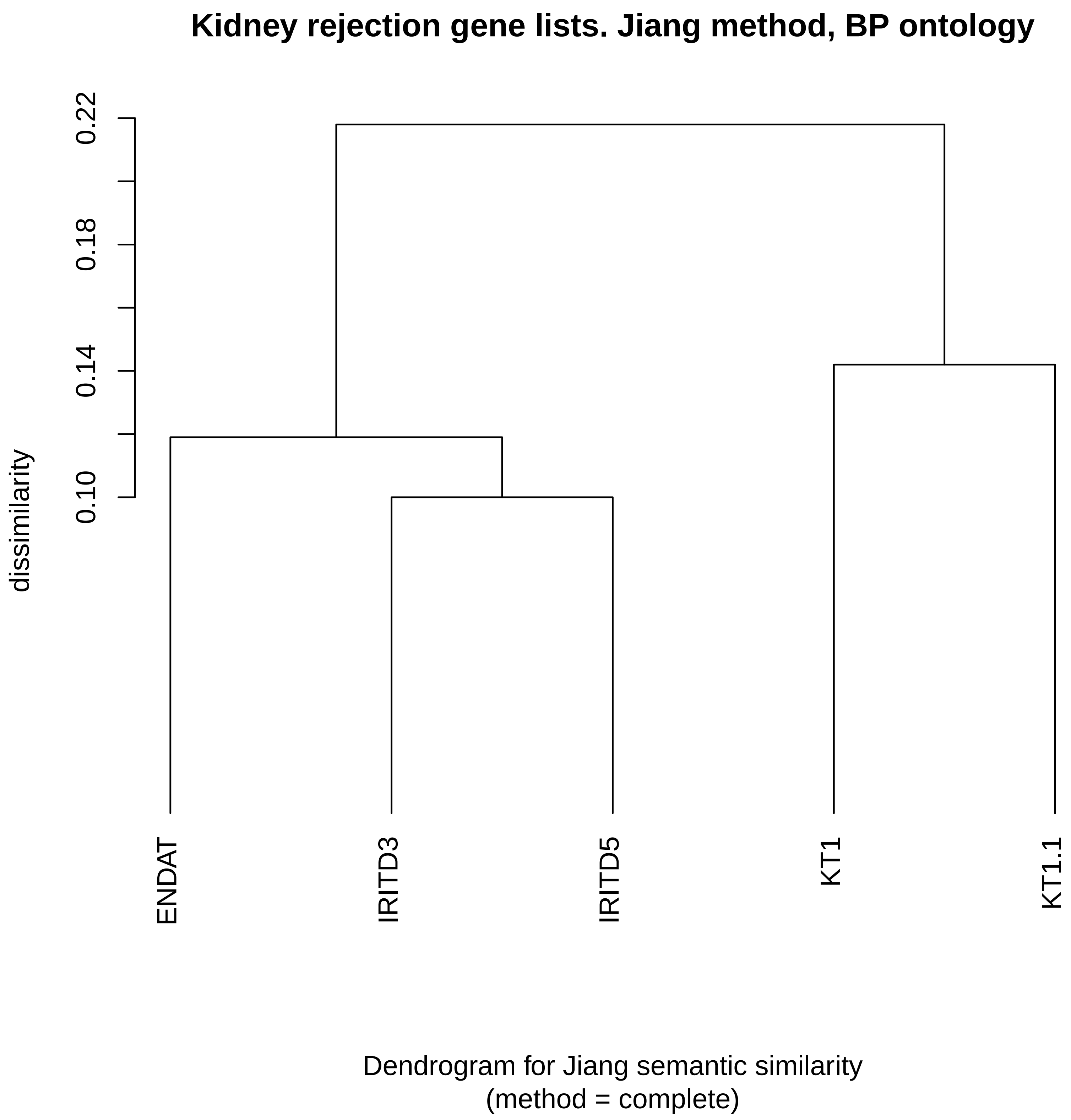
Kidney rejection gene lists. Jiang method, BP ontology
dissimilarity
0.10
0.14
0.18
0.22
ENDAT
IRITD3
IRITD5
KT1
KT1.1
Dendrogram for Jiang semantic similarity
(method = complete)

Kidney rejection gene lists. Rel method, BP ontology

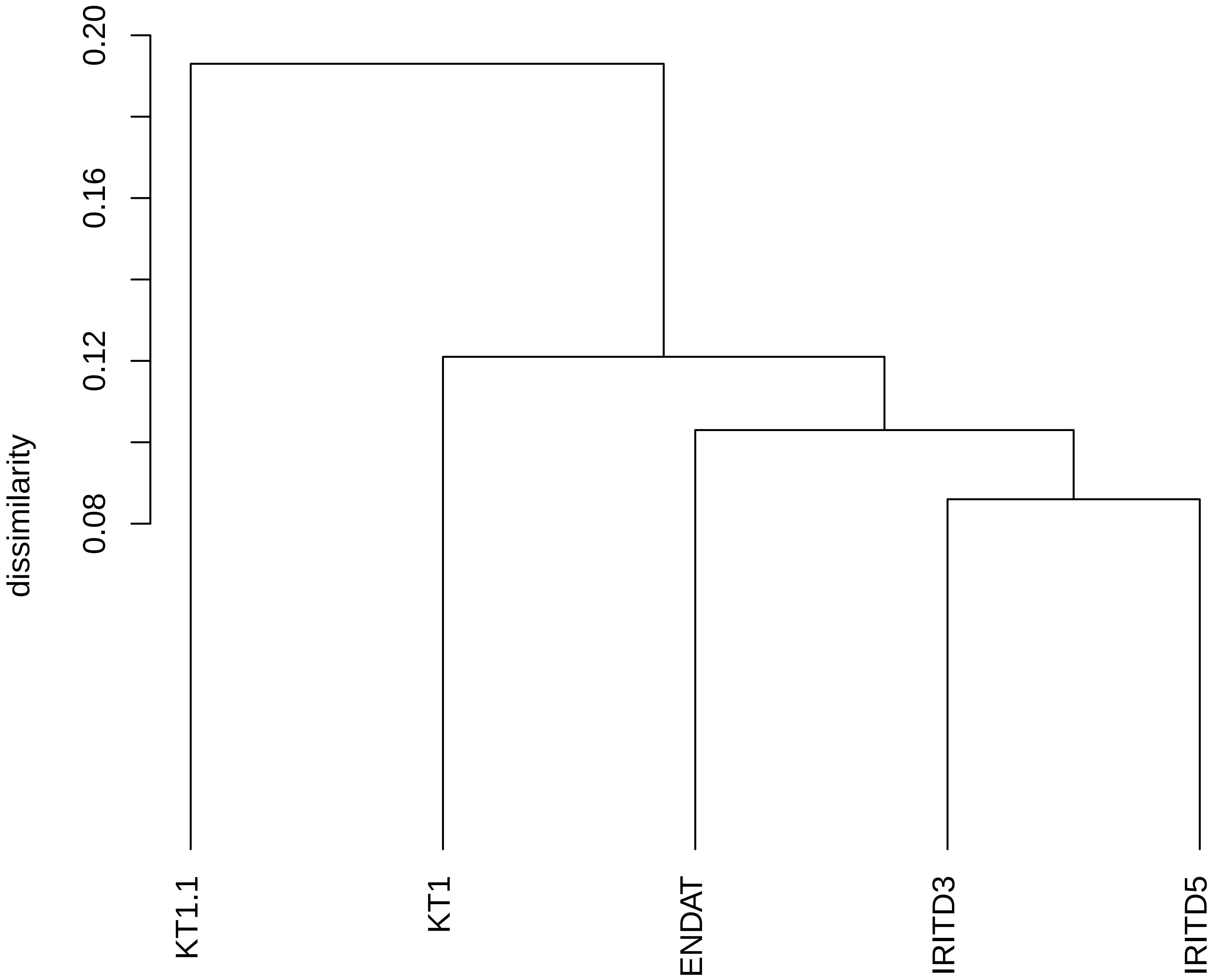

Dendrogram for Rel semantic similarity
(method = complete)

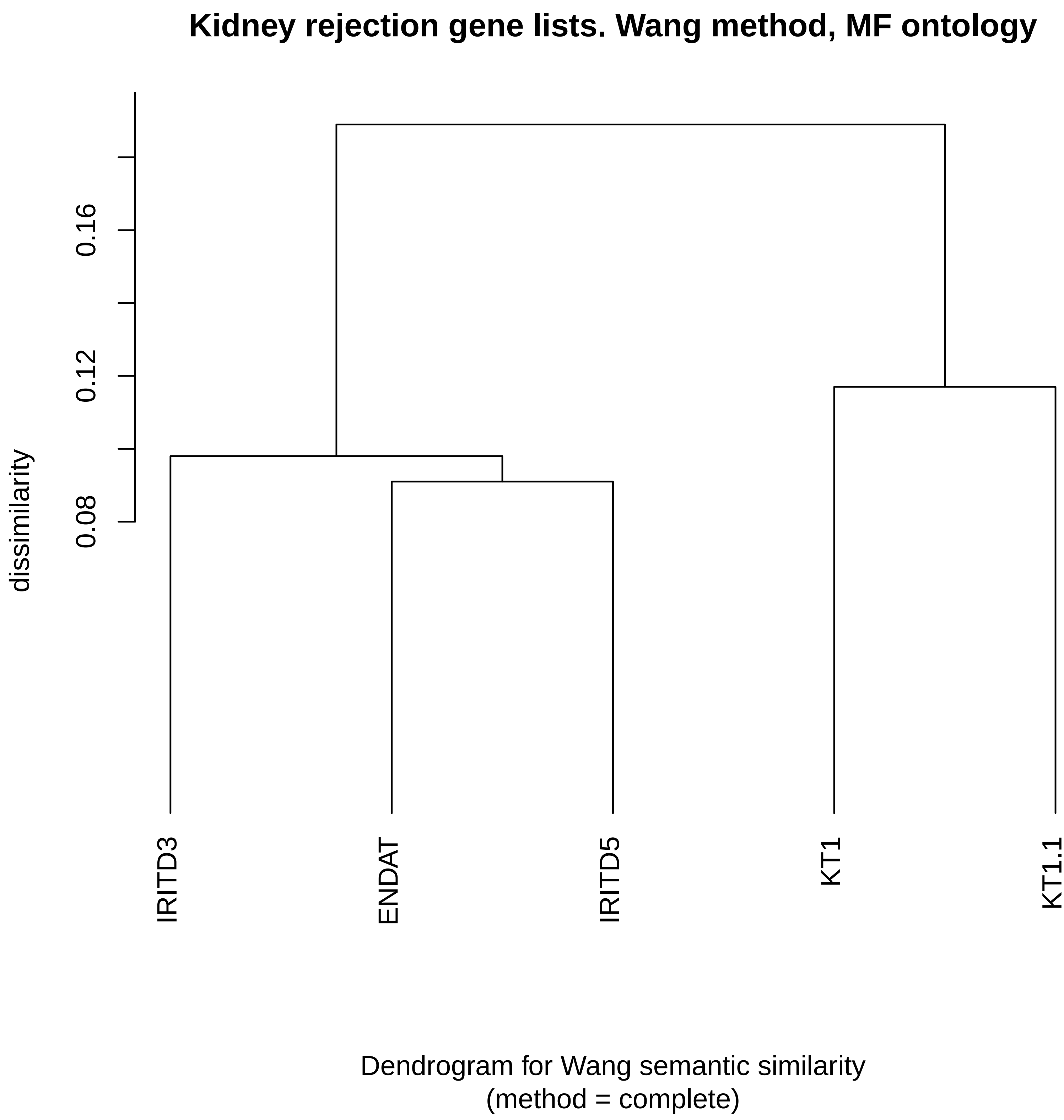

Kidney rejection gene lists. Wang method, MF ontology
dissimilarity
0.08
0.12
0.16
IRITD3
ENDAT
IRITD5
KT1
KT1.1
Dendrogram for Wang semantic similarity
(method = complete)

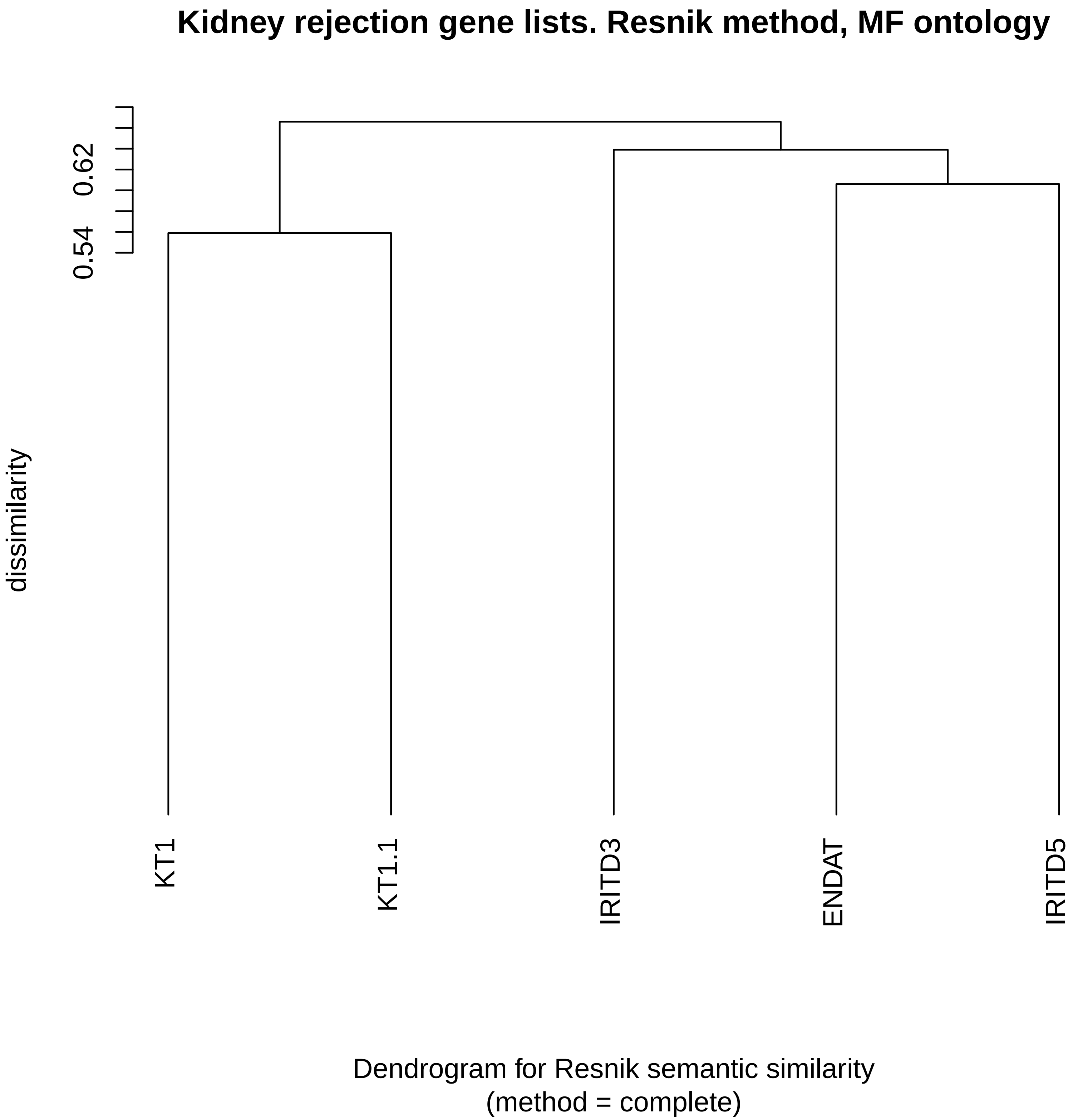

Kidney rejection gene lists. Resnik method, MF ontology
0.54
0.62
dissimilarity
KT1
KT1.1
IRITD3
ENDAT
IRITD5
Dendrogram for Resnik semantic similarity
(method = complete)

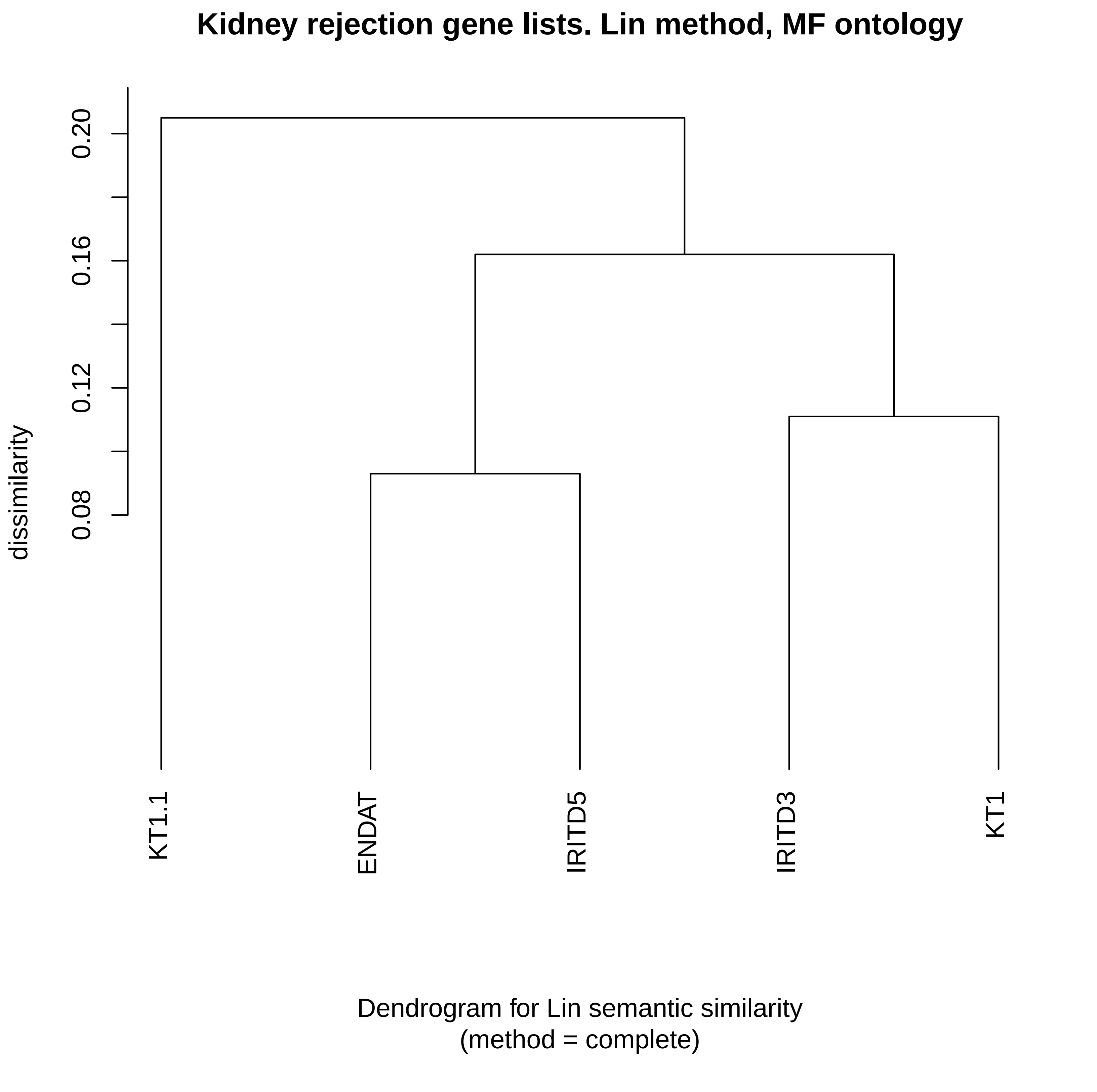
Kidney rejection gene lists. Lin method, MF ontology
dissimilarity
0.08
0.12
0.16
0.20
KT1.1
ENDAT
IRITD5
IRITD3
KT1
Dendrogram for Lin semantic similarity
(method = complete)

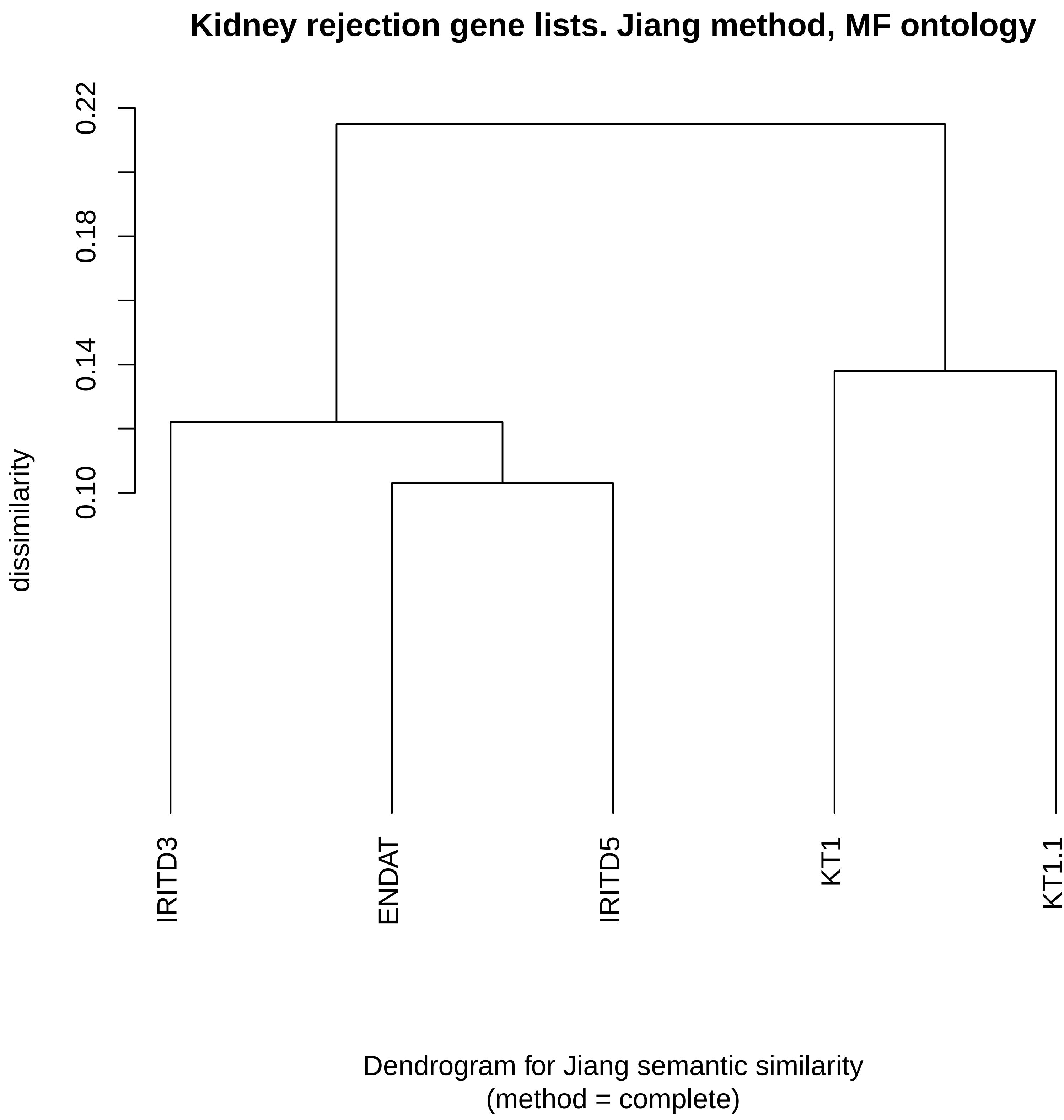
Kidney rejection gene lists. Jiang method, MF ontology
dissimilarity
0.10
0.14
0.18
0.22
IRITD3
ENDAT
IRITD5
KT1
KT1.1
Dendrogram for Jiang semantic similarity
(method = complete)

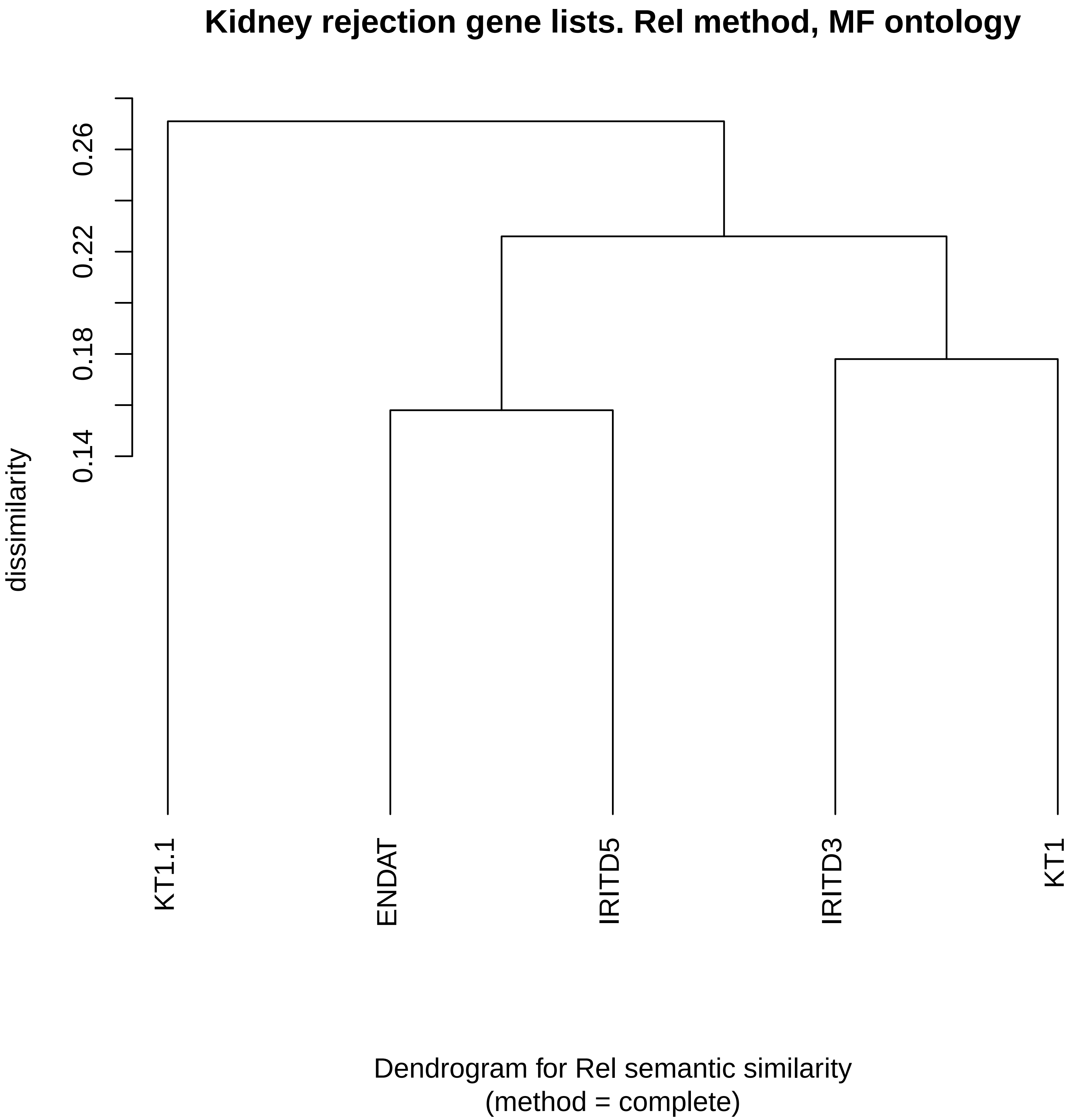

Kidney rejection gene lists. Rel method, MF ontology
dissimilarity
0.14
0.18
0.22
0.26
KT1.1
ENDAT
IRITD5
IRITD3
KT1
Dendrogram for Rel semantic similarity
(method = complete)

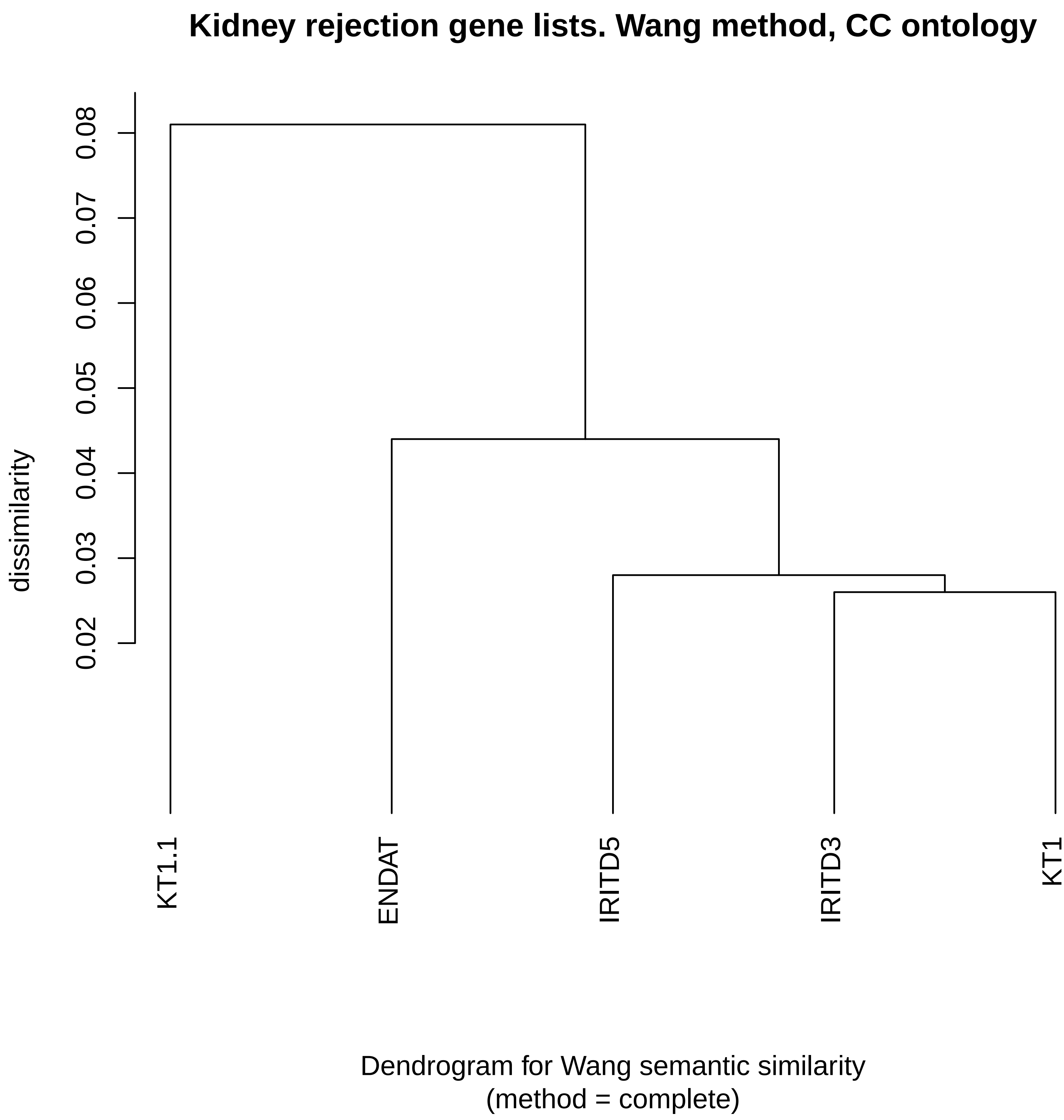
Kidney rejection gene lists. Wang method, CC ontology
dissimilarity
0.02
0.03
0.04
0.05
0.06
0.07
0.08
KT1.1
ENDAT
IRITD5
IRITD3
KT1
Dendrogram for Wang semantic similarity
(method = complete)

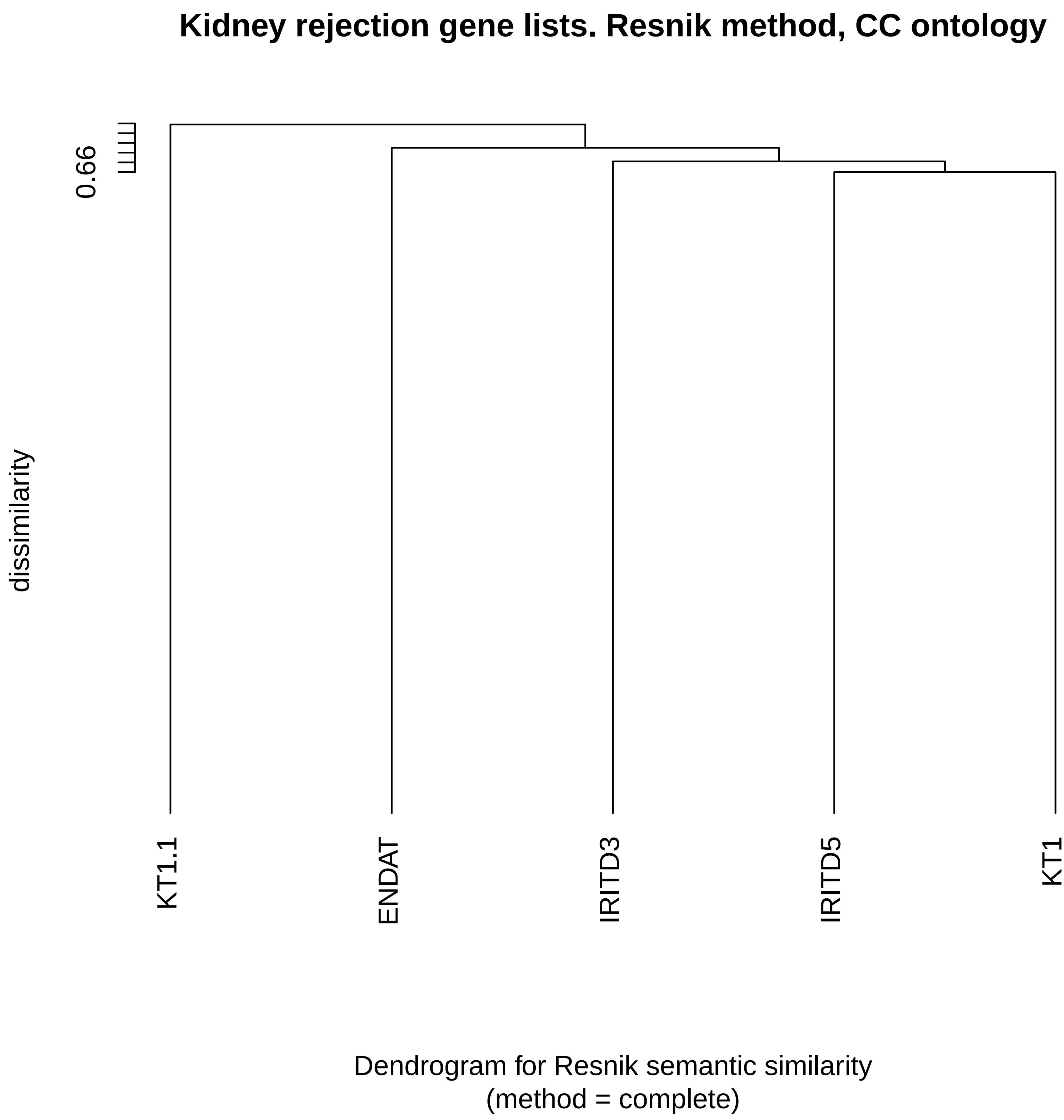

Kidney rejection gene lists. Resnik method, CC ontology
0.66
dissimilarity
KT1.1
ENDAT
IRITD3
IRITD5
KT1
Dendrogram for Resnik semantic similarity
(method = complete)

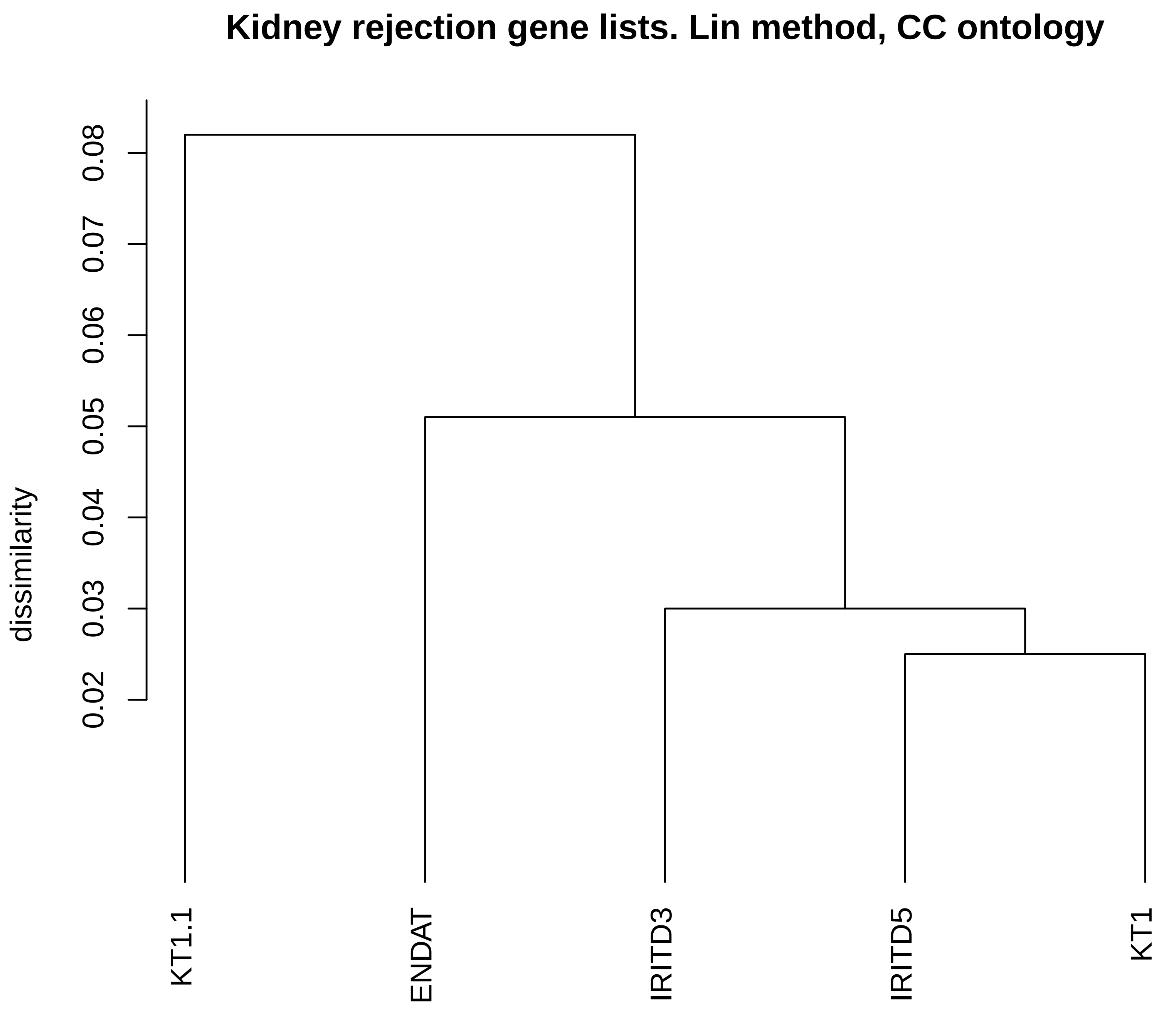

Dendrogram for Lin semantic similarity
(method = complete)

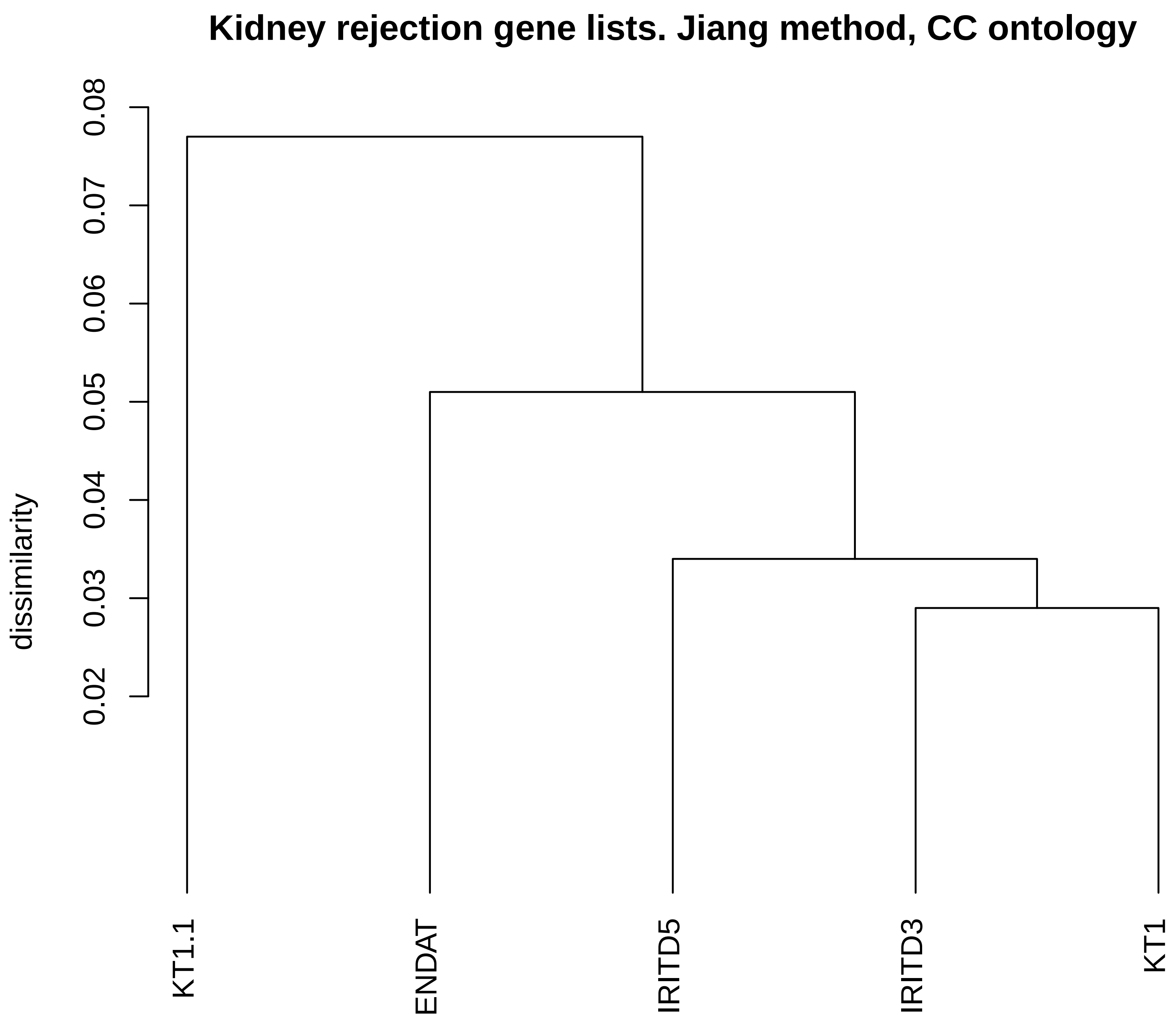

Dendrogram for Jiang semantic similarity
(method = complete)

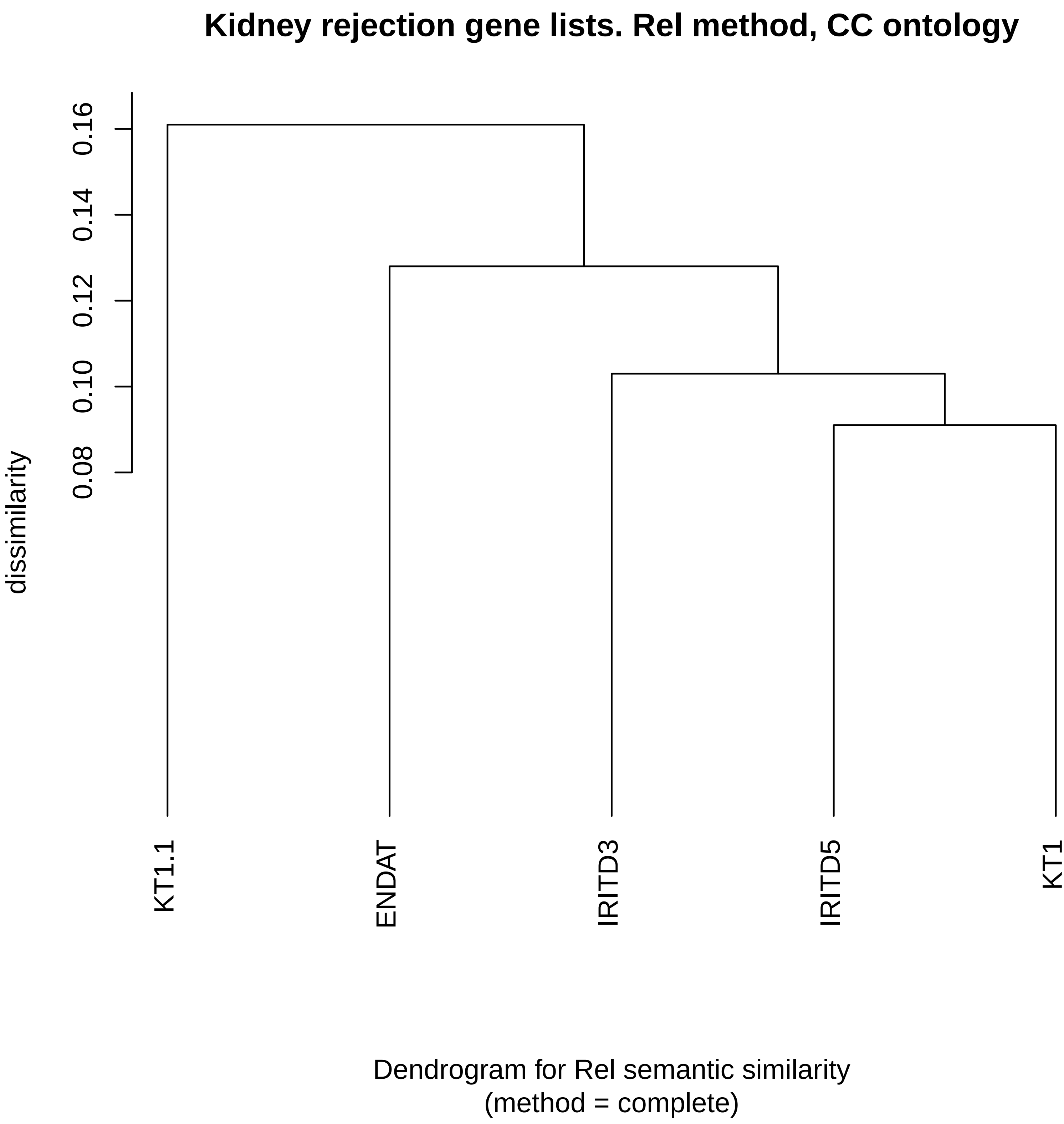
Kidney rejection gene lists. Rel method, CC ontology
0.16
0.14
0.12
0.10
0.08
dissimilarity
KT1.1
ENDAT
IRITD3
IRITD5
KT1
Dendrogram for Rel semantic similarity
(method = complete)
