# Supplementary material for: An equivalence approach to the integrative analysis of feature lists
Source: BMC Bioinformatics. 2019 Aug 27;20:441. doi: 10.1186/s12859-019-3008-x (PMC6712676; doi:10.1186/s12859-019-3008-x)
Supplement: Supplementary file 4 — This file contains an extended version of the Cancer data analysis example. Plots of the dendrograms produced up to the 8th level of the GO are shown. Analysis of the data based on Semantic Similarity (SS) are provided. Informal comparisons among results obtained using distinct SS measures and with results obtained with goprofiles are presented. Each plot is in a separate page to facilitate visualization. (PDF 187 kb) [file 12859_2019_3008_MOESM4_ESM.pdf]

Equivalence Analysis of the Comprehensive Collection of Cancer Related Gene Lists (allOnco)

Supplementary materials to Sanchez-Pla, A., Salicru, M. and Ocaña, J. An equivalence approach to the integrative analysis of features lists. BMC Bioinformatics

Here we consider a series of lists that have been obtained from Bushman lab (http://www.bushmanlab.org/links/genelists). The lists contain `Entrez` identifiers for each gene so the only preprocessing consisted of removing one list that contained less than 100 genes. Table 1 contains, for each list, the name, the number of genes, the species and a short description.

Table 1: Cancer Gene Lists. Cancer related genes that were used to generate a comprehensive list (allOnco) that is comprised of the union of all lists The lists have been selected from the datasets available in the file ”allOnco.tsv” downloaded from the url http://www.bushmanlab.org/links/genelists. Since the file has been downloaded the web page has changed and this file is not available anymore, although new version of the file can be found in the site.

Set	Size	Species	Description
Atlas	989	human	Genes: hybrid gene found in at least one cancer case, or gene amplification or homozygous deletion found in a significant subset of cases in a given cancer-type.
CANgenes	189	human	191 common genes that were mutated at significant frequency in all tumors of human breast and colorectal cancers.
CIS (RTCGD)	587	multiple	Retroviral insertional mutagenesis in mouse hematopoietic tumors.
Miscellaneous	187	multiple	From Cold Spring Harbor Retroviruses Chapter on Oncogenes, an early version of the CIS database, a list from Dr. Tony Hunter, and misc. additions from the literature.
Sanger	452	human	Compilation from literature: ”genes that are mutated and causally implicated in cancer development”
Vogelstein	420	human	Cancer genes related to chromosomal breakpoints
Waldman	455	Human	Gene set is from the Waldman gene database and lists cancer genes sorted by chromosomal locus and includes links to OMIM.

Citing the researcher’s description of the lists they are *collections of cancer-related genes that were used to generate a comprehensive list (allOnco) that is comprised of the union of all lists*. In this case, we do not have any “a priori” expectations of which lists should be equivalent to which but, instead, we can rely on equivalence analysis to help answer the question “up to what point can these lists be considered equivalent” so that they can be merged in a single list?

Equivalence analysis of the resulting gene lists can be easily performed using

functions in the goProfiles package. A standard analysis has been performed which consists of computing the dissimilarity matrix of equivalence thresholds and building a dendrogram (here using the maximum distance, or complete, method) for the three GO ontologies, BP, MF and CC, at levels 2 to 8.

```
> library(goProfiles)
> load("allOncoGeneLists.Rda")
> # Number of genes in each list:
> sapply(geneLists, length)

        atlas      cangenes           cis    humanlymph miscellaneous
          991           189           613            38           187
       sanger    Vogelstein       waldman
          450           419           426

> # Analysis restricted to lists with almost 100 genes:
> ngen.min <- 100
> geneLists <- geneLists[sapply(geneLists, length) >= ngen.min]
> sapply(geneLists, length)

        atlas      cangenes           cis miscellaneous        sanger
          991           189           613           187           450
   Vogelstein       waldman
          419           426

> # Number of common genes to each pair of lists:
> lstNams <- names(geneLists)
> for (i in 2:length(geneLists)) {
+   for (j in 1:(i-1)) {
+     cat(lstNams[i], "&", lstNams[j],
+     length(intersect(geneLists[[i]], geneLists[[j]])),
+     "common genes of",
+     length(geneLists[[i]]), length(geneLists[[j]]), "\n")
+   }
+ }

cangenes & atlas 27 common genes of 189 991
cis & atlas 107 common genes of 613 991
cis & cangenes 8 common genes of 613 189
miscellaneous & atlas 83 common genes of 187 991
miscellaneous & cangenes 8 common genes of 187 189
miscellaneous & cis 19 common genes of 187 613
sanger & atlas 292 common genes of 450 991
sanger & cangenes 17 common genes of 450 189
sanger & cis 77 common genes of 450 613
sanger & miscellaneous 60 common genes of 450 187
Vogelstein & atlas 261 common genes of 419 991
```

```
Vogelstein & cangenes 18 common genes of 419 189
Vogelstein & cis 68 common genes of 419 613
Vogelstein & miscellaneous 57 common genes of 419 187
Vogelstein & sanger 364 common genes of 419 450
waldman & atlas 164 common genes of 426 991
waldman & cangenes 14 common genes of 426 189
waldman & cis 56 common genes of 426 613
waldman & miscellaneous 123 common genes of 426 187
waldman & sanger 104 common genes of 426 450
waldman & Vogelstein 100 common genes of 426 419
```

```
> # Number of annotated genes in each ontology and GO level:
> # (quite time consuming, results are included below)
> # for (lev in 2:16) {
> #   cat("level ", lev, "\n")
> #   profsList <- lapply(geneLists, expandedProfile,
> #                       level = lev, orgPackage = "org.Hs.eg.db")
> #   print(sapply(profsList, function(ontoProf){
> #     sapply(ontoProf, ngenes)
> #   }))
> # }
>
> # Equivalence analysis from GO levels 2 to 8 and for all ontologies:
> # (next sentence is considerably time consuming,
> # you may go directly to load(file = "cancer_gene_lists_ etc. uncomment, and run it)
> genListsClusters <- iterEquivClust(geneLists, ontoLevels = 2:8,
+                                    jobName =
+                                    "cancer_lists_equivalence_clustering_levels2to8",
+                                    ylab = "Equivalence threshold distance",
+                                    orgPackage="org.Hs.eg.db")
```

```
 cancer_lists_equivalence_clustering_levels2to8 Ontology BP at level 2

Building marginal profiles:

Building profile for list  atlas
Building profile for list  cangenes
Building profile for list  cis
Building profile for list  miscellaneous
Building profile for list  sanger
Building profile for list  Vogelstein
Building profile for list  waldman

Building intersection profiles:

cangenes     ,atlas        |
```

```
cis          ,atlas        |cis          ,cangenes     |
miscellaneous,atlas        |miscellaneous,cangenes     |miscellaneous,cis          |
sanger       ,atlas        |sanger       ,cangenes     |sanger       ,cis          |
sanger       ,miscellaneous|
Vogelstein   ,atlas        |Vogelstein   ,cangenes     |Vogelstein   ,cis          |
Vogelstein   ,miscellaneous|Vogelstein   ,sanger       |
waldman      ,atlas        |waldman      ,cangenes     |waldman      ,cis          |
waldman      ,miscellaneous|waldman      ,sanger       |waldman      ,Vogelstein   |

Performing all equivalence tests:

cangenes     ,atlas        |
cis          ,atlas        |cis          ,cangenes     |
miscellaneous,atlas        |miscellaneous,cangenes     |miscellaneous,cis          |
sanger       ,atlas        |sanger       ,cangenes     |sanger       ,cis          |
sanger       ,miscellaneous|
Vogelstein   ,atlas        |Vogelstein   ,cangenes     |Vogelstein   ,cis          |
Vogelstein   ,miscellaneous|Vogelstein   ,sanger       |
waldman      ,atlas        |waldman      ,cangenes     |waldman      ,cis          |
waldman      ,miscellaneous|waldman      ,sanger       |waldman      ,Vogelstein   |

 cancer_lists_equivalence_clustering_levels2to8 Ontology BP at level 3

Building marginal profiles:

Etc. Truncated script output...


> save(genListsClusters,
+      file = paste0(attr(genListsClusters, "jobName"), ".rda", sep =""))
> # load(file = "cancer_gene_lists_equivalence_clustering_levels2to8.rda")
>
> # Generate a pdf file with all equivalence clusters:
> equivClust2pdf(genListsClusters,
+                jobName = "Cancer_gene_lists_equivalence_method")
```

The lists tend to group consistently within the ontologies groupings at distinct levels of the ontologies are similar but these groupings can change from one ontology to another, which is not strange because they refer to different concepts. Depending on what the goal of merging the gene lists is the different groupings of each ontology can be used as a guide to decide whether a given dataset should be included or not in a common list. For instance depending on whether what one wishes to obtain is a heterogeneous or a homogeneous list one could decide to include groups that are separated by a higher threshold or, instead, that are near each other in the dendrogram.

Despite these general trends, there is some variability between the clusters obtained at different levels. In our opinion a trade-off between the need for

statistical validity vs the need for interesting biological information must be considered always. Provided its asymptotic inferential character (that is to say, more sample size i.e., more total annotation would imply more reliability in the inferences), one may expect more stability in the results for large sample sizes. Total annotation may decline if we require more specificity to GO terms, if we go deep in the GO. On the other hand, more specificity provides more interesting biological information; at lower levels the GO terms under consideration may be too general. The total annotation numbers for each ontology and progressively deep levels are displayed after the dendrogram pages. For the BP ontology, all sample sizes stay high (clearly over 100) even at the deepest levels analyzed here. So, possibly the most interesting picture of the equivalence relations between these lists is provided at level 8. A similar comment applies to ontology CC. On the other hand, for ontology MF, the number of annotated genes for list "cangenes" (and to a lesser extent "miscellaneous") clearly declines with depth in the GO. This is associated to a great indeterminacy on its group membership from level 6 hereunder.

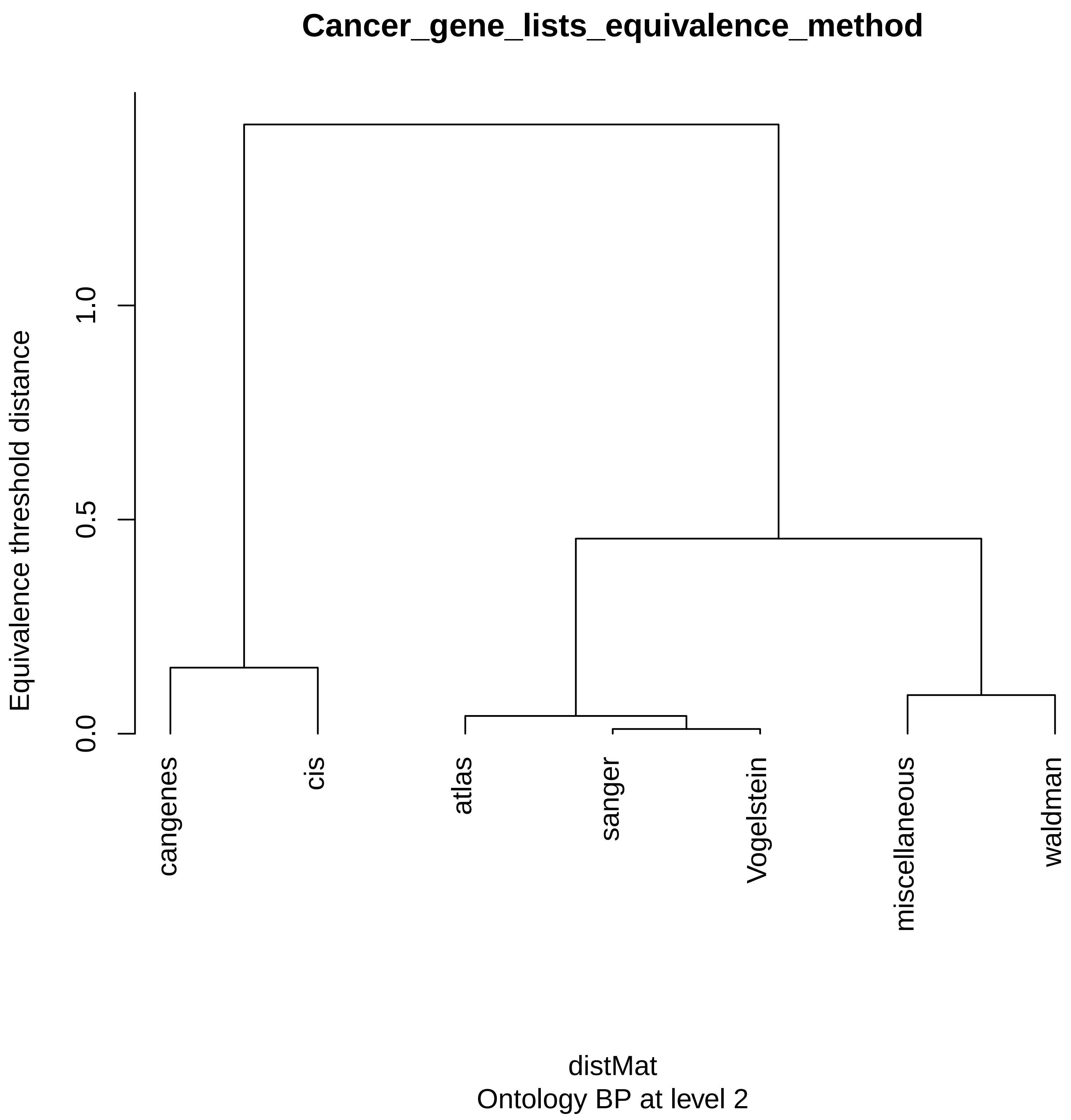
Cancer_gene_lists_equivalence_method
Equivalence threshold distance
0.0
0.5
1.0
cangenes
cis
atlas
sanger
Vogelstein
miscellaneous
waldman
distMat
Ontology BP at level 2

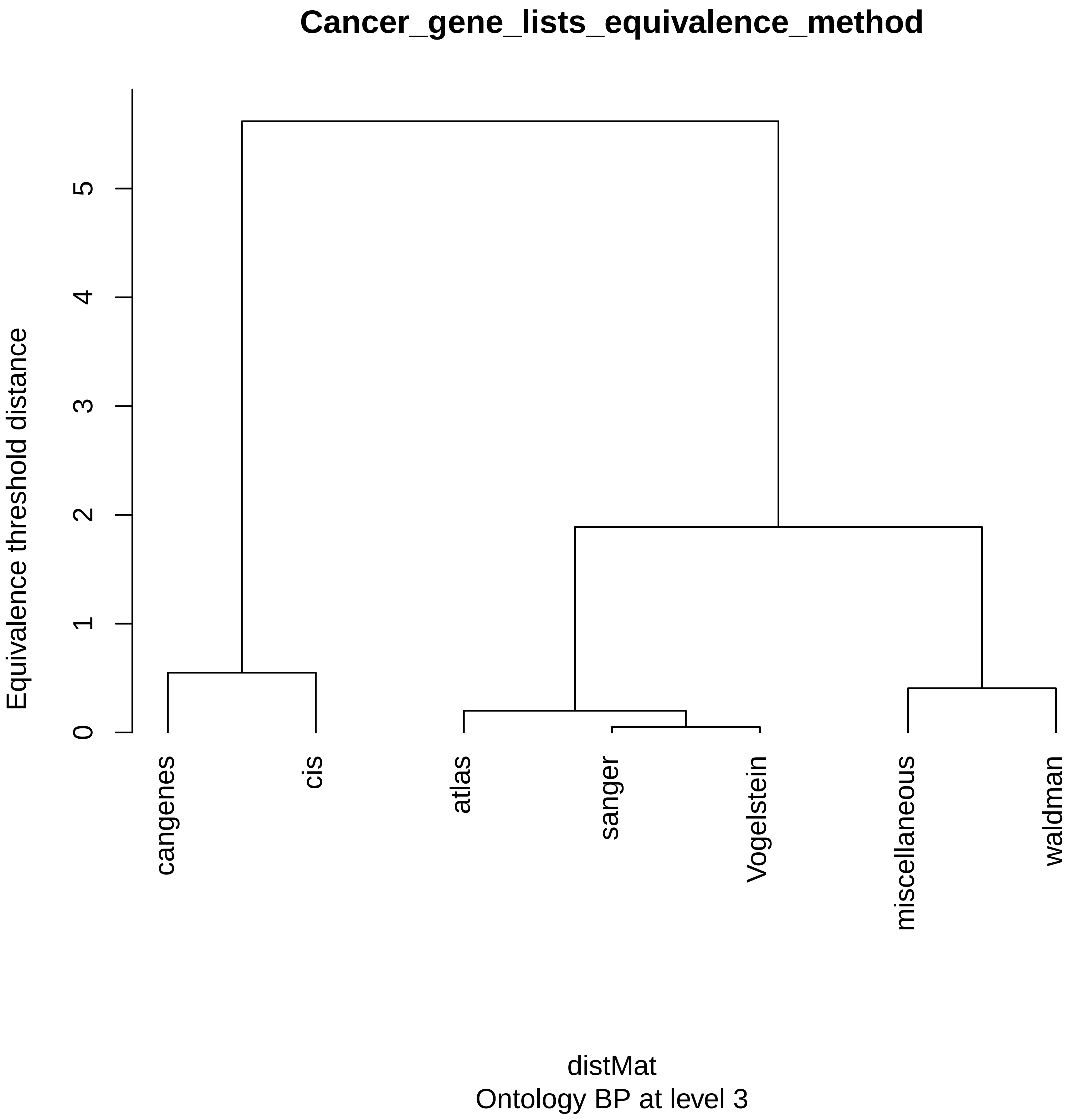
Cancer_gene_lists_equivalence_method
Equivalence threshold distance
0
1
2
3
4
5
cangenes
cis
atlas
sanger
Vogelstein
miscellaneous
waldman
distMat
Ontology BP at level 3

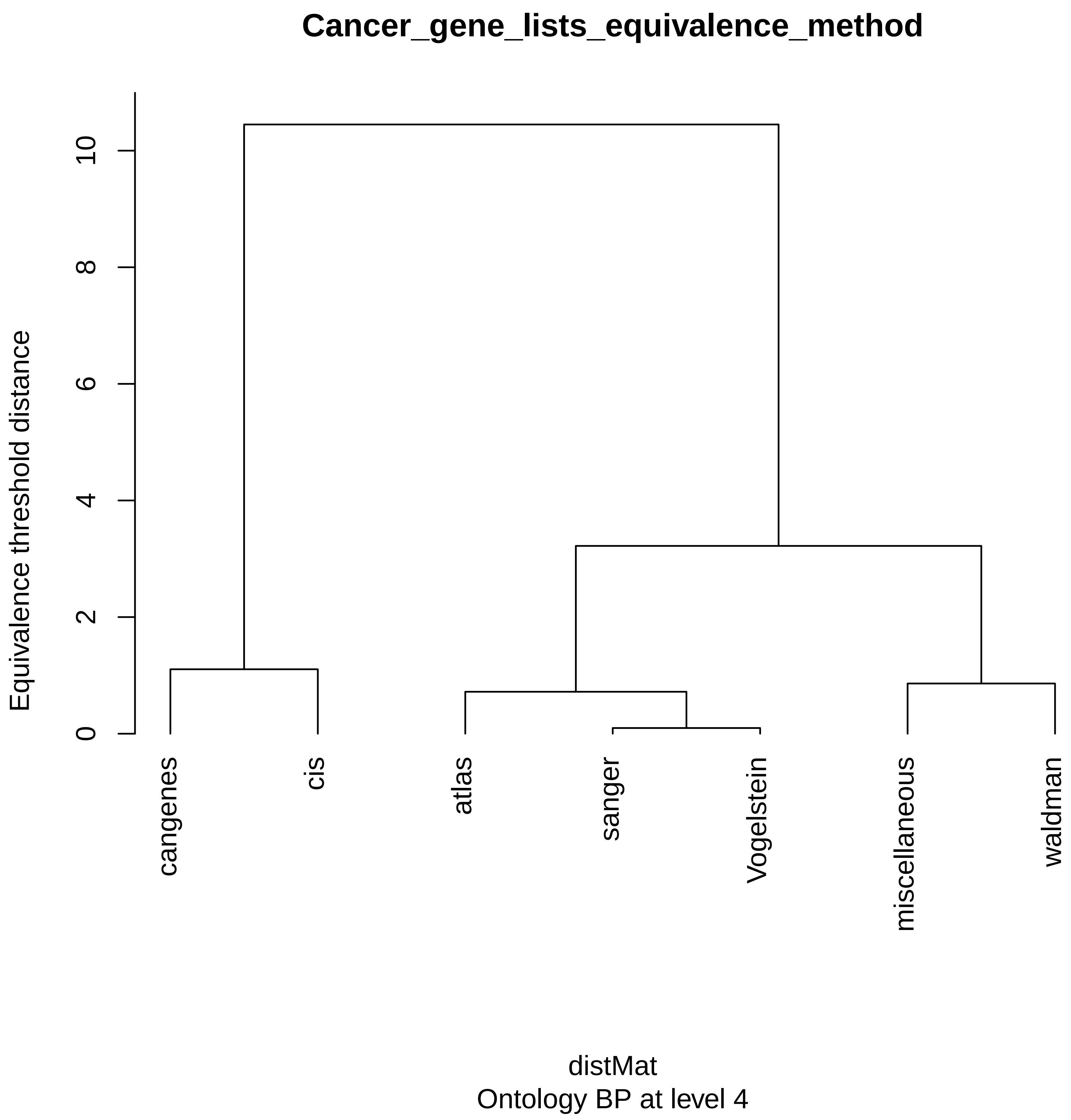
Cancer_gene_lists_equivalence_method
Equivalence threshold distance
0
2
4
6
8
10
cangenes
cis
atlas
sanger
Vogelstein
miscellaneous
waldman
distMat
Ontology BP at level 4

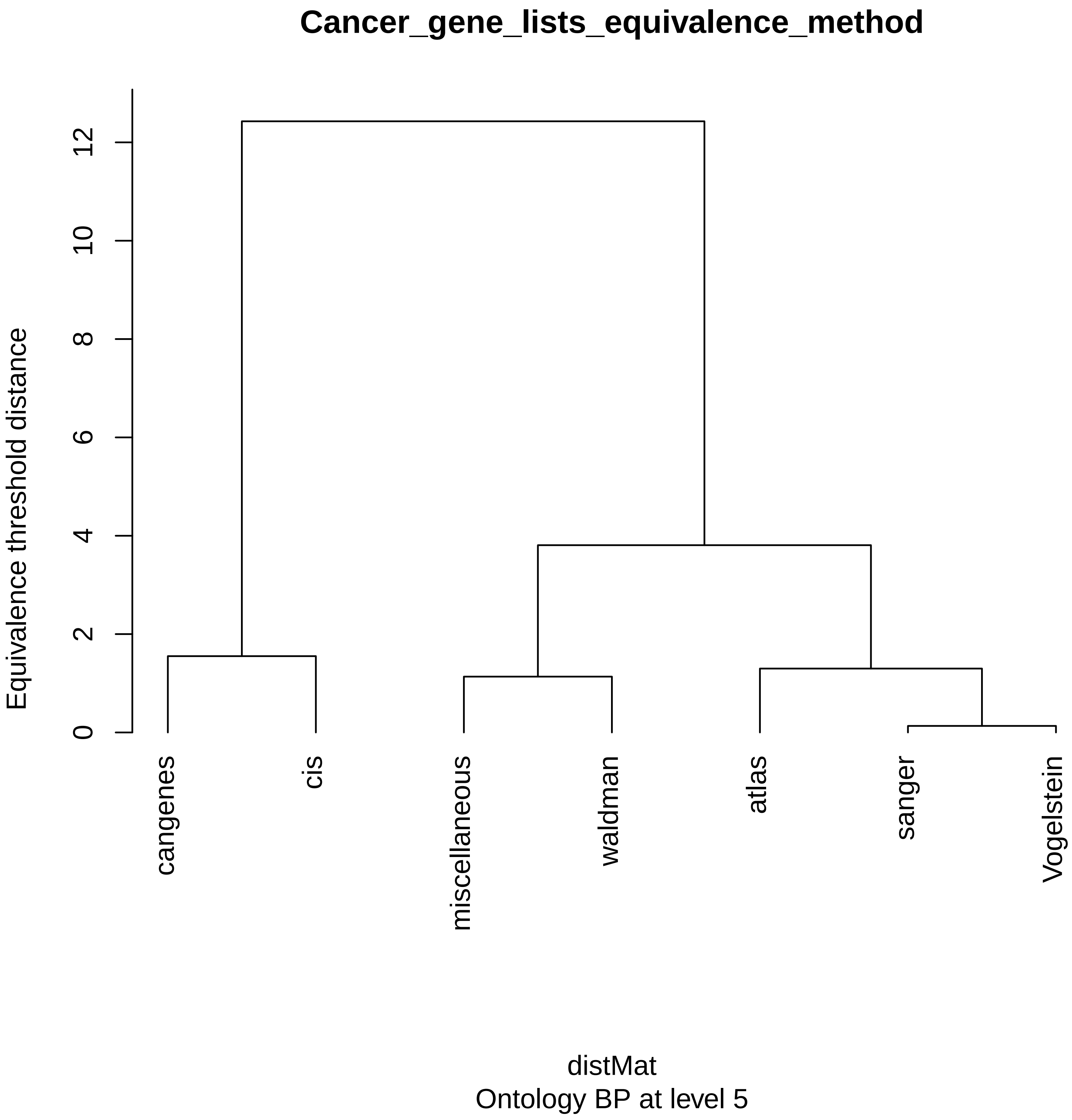
Cancer_gene_lists_equivalence_method
Equivalence threshold distance
0
2
4
6
8
10
12
cangenes
cis
miscellaneous
waldman
atlas
sanger
Vogelstein
distMat
Ontology BP at level 5

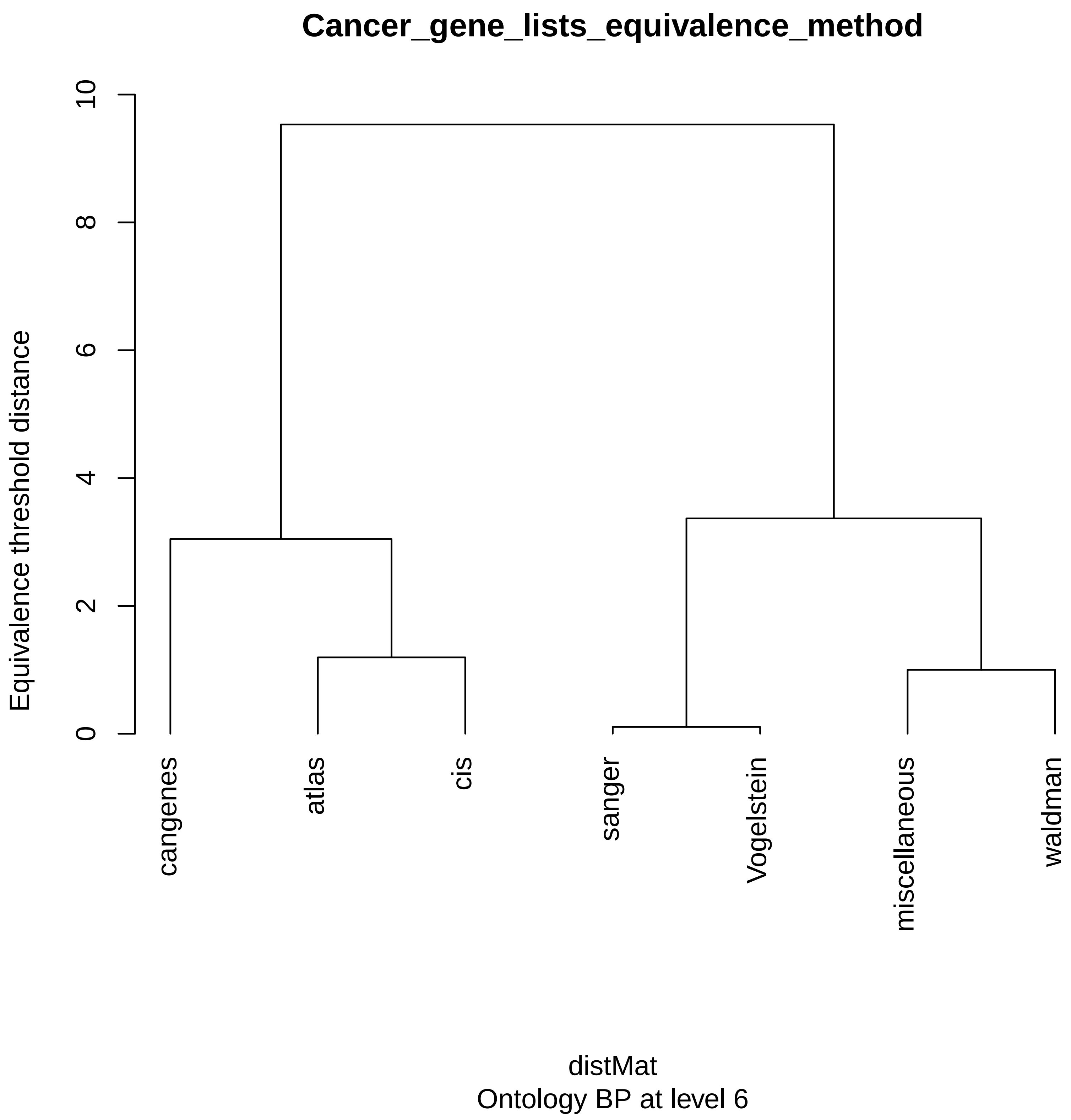
Cancer_gene_lists_equivalence_method
Equivalence threshold distance
0
2
4
6
8
10
cangenes
atlas
cis
sanger
Vogelstein
miscellaneous
waldman
distMat
Ontology BP at level 6

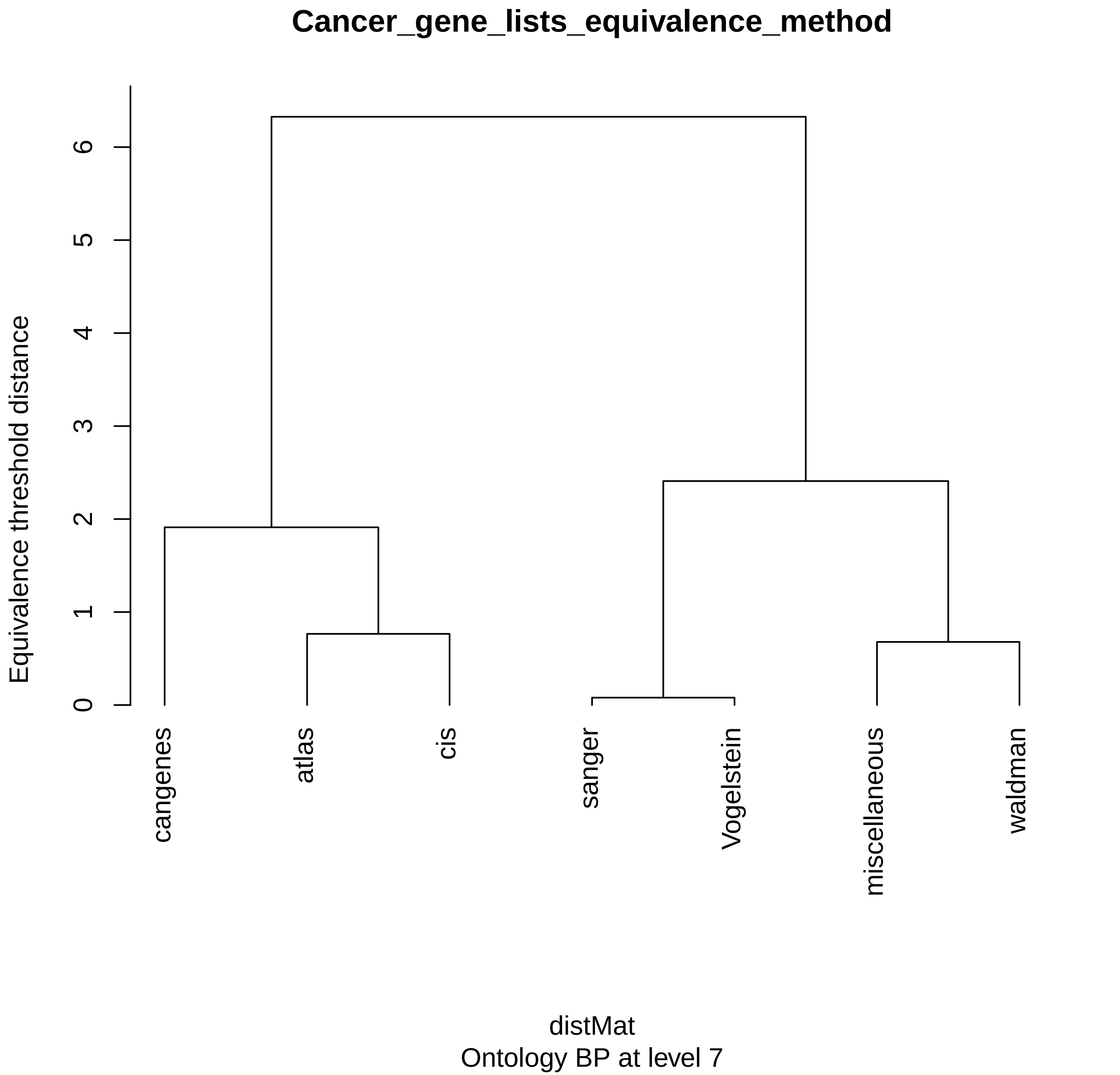
Cancer_gene_lists_equivalence_method
Equivalence threshold distance
0
1
2
3
4
5
6
cangenes
atlas
cis
sanger
Vogelstein
miscellaneous
waldman
distMat
Ontology BP at level 7

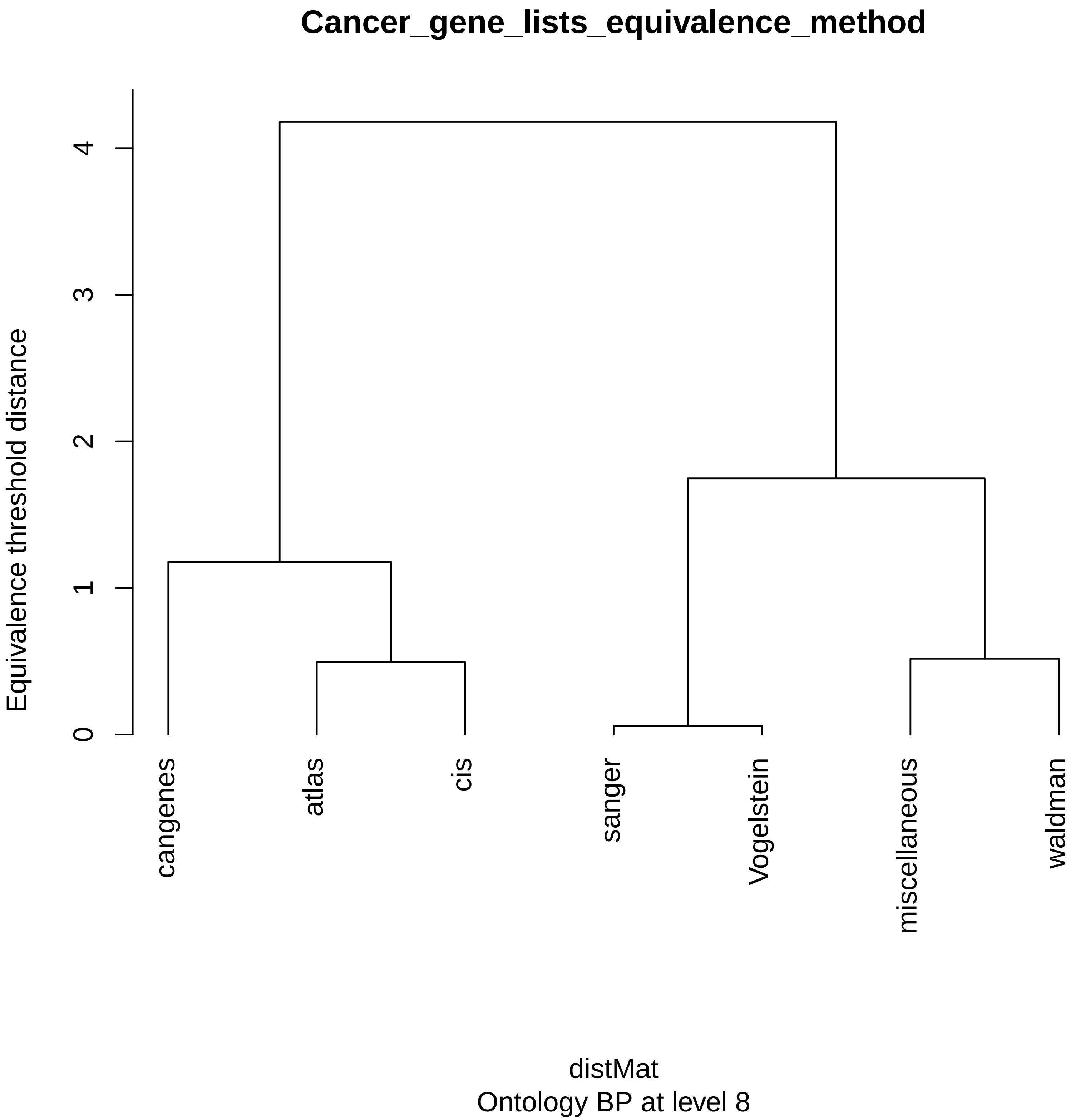
Cancer_gene_lists_equivalence_method
Equivalence threshold distance
0
1
2
3
4
cangenes
atlas
cis
sanger
Vogelstein
miscellaneous
waldman
distMat
Ontology BP at level 8

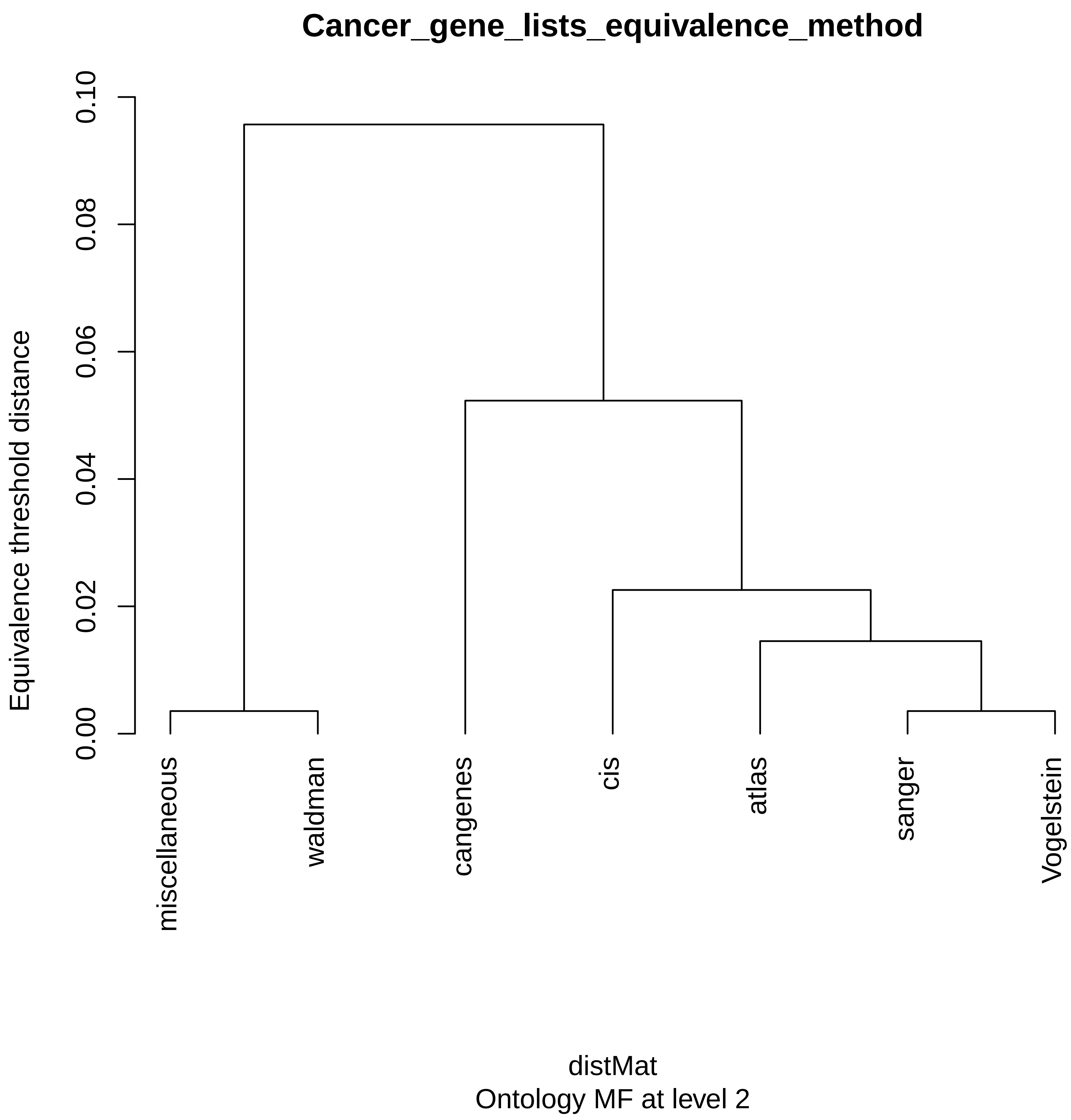
Cancer_gene_lists_equivalence_method
Equivalence threshold distance
0.00
0.02
0.04
0.06
0.08
0.10
miscellaneous
waldman
cangenes
cis
atlas
sanger
Vogelstein
distMat
Ontology MF at level 2

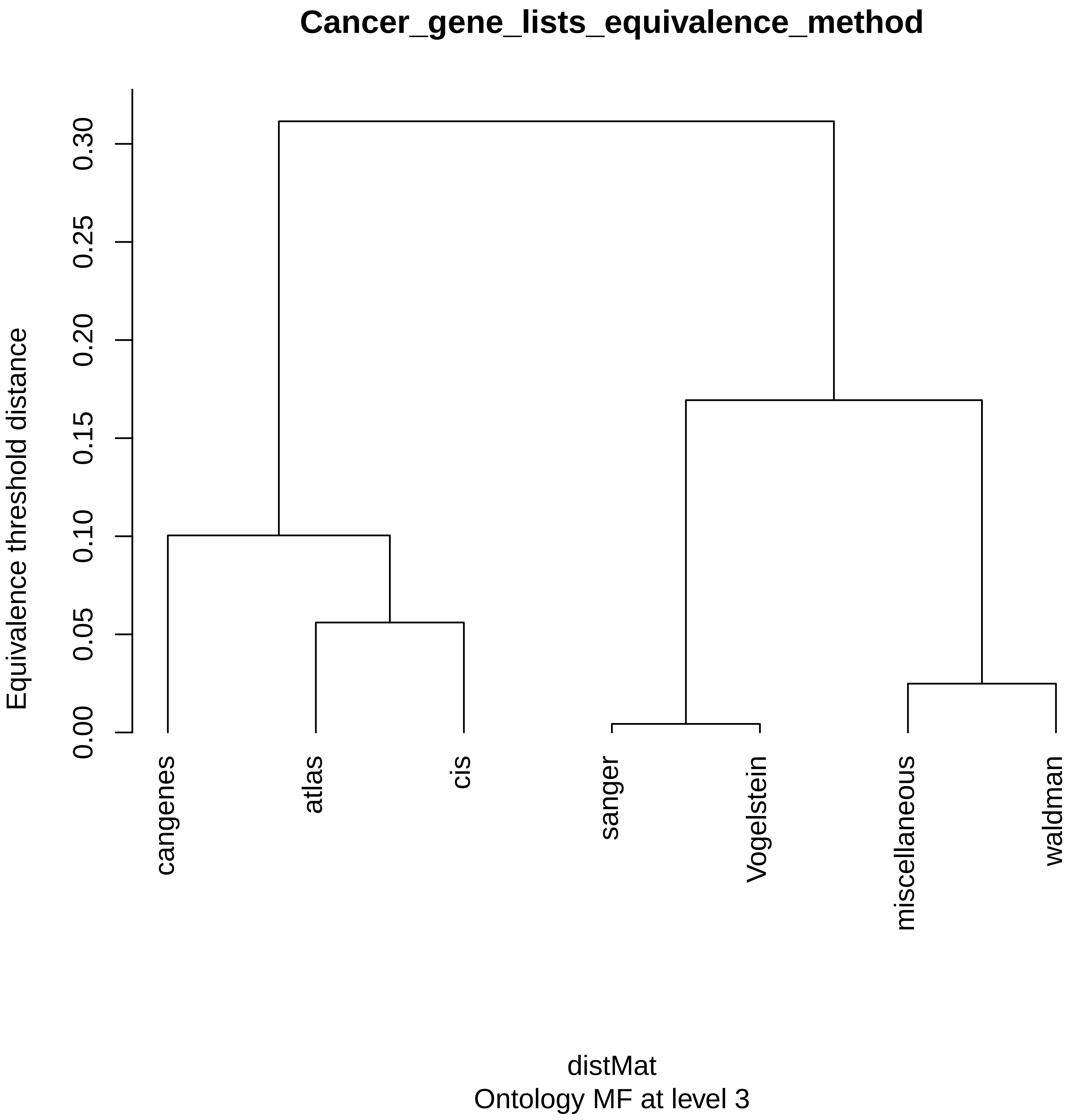

Cancer_gene_lists_equivalence_method
Equivalence threshold distance
0.00
0.05
0.10
0.15
0.20
0.25
0.30
cangenes
atlas
cis
sanger
Vogelstein
miscellaneous
waldman
distMat
Ontology MF at level 3

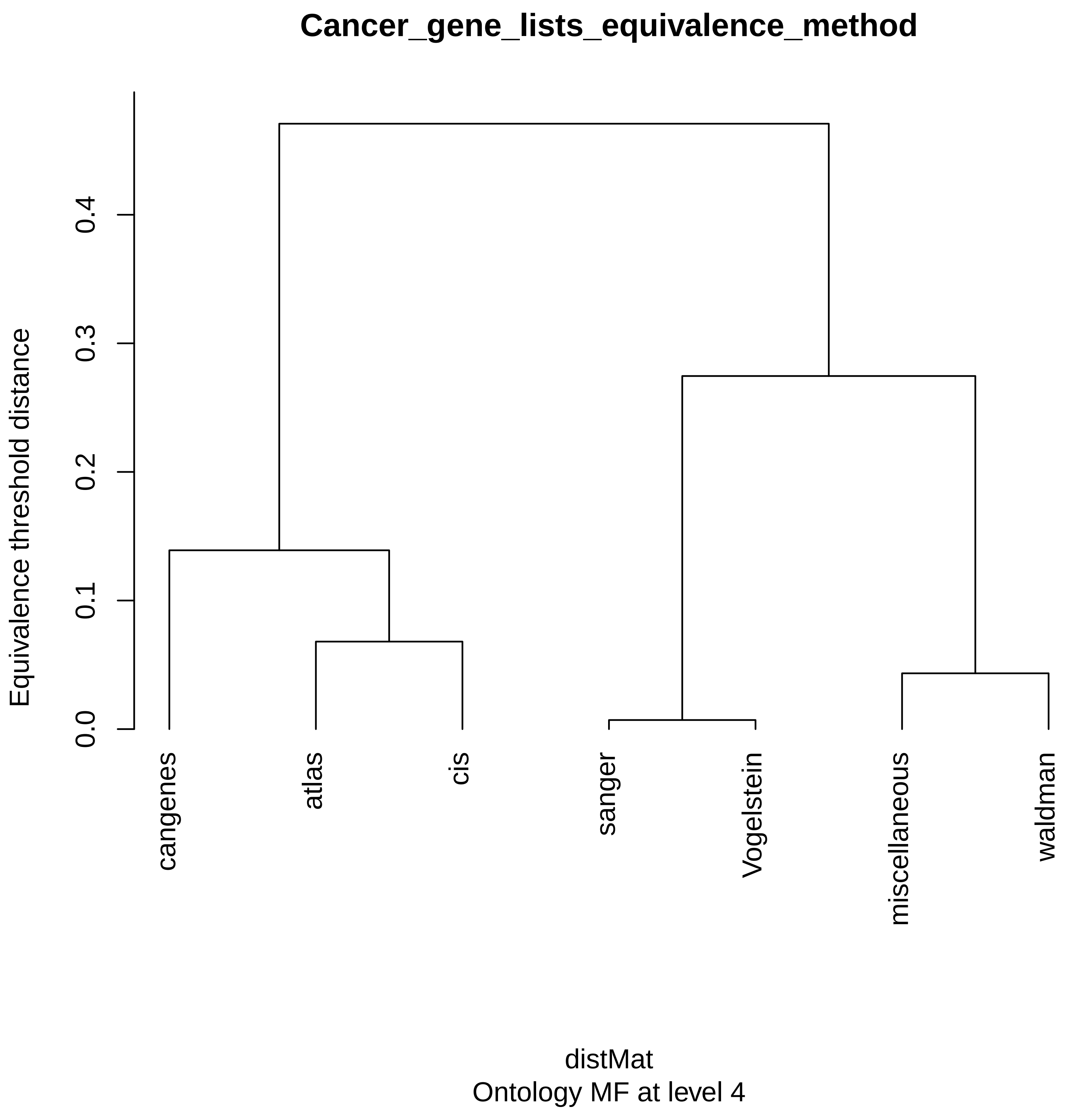
Cancer_gene_lists_equivalence_method
Equivalence threshold distance
0.0
0.1
0.2
0.3
0.4
cangenes
atlas
cis
sanger
Vogelstein
miscellaneous
waldman
distMat
Ontology MF at level 4

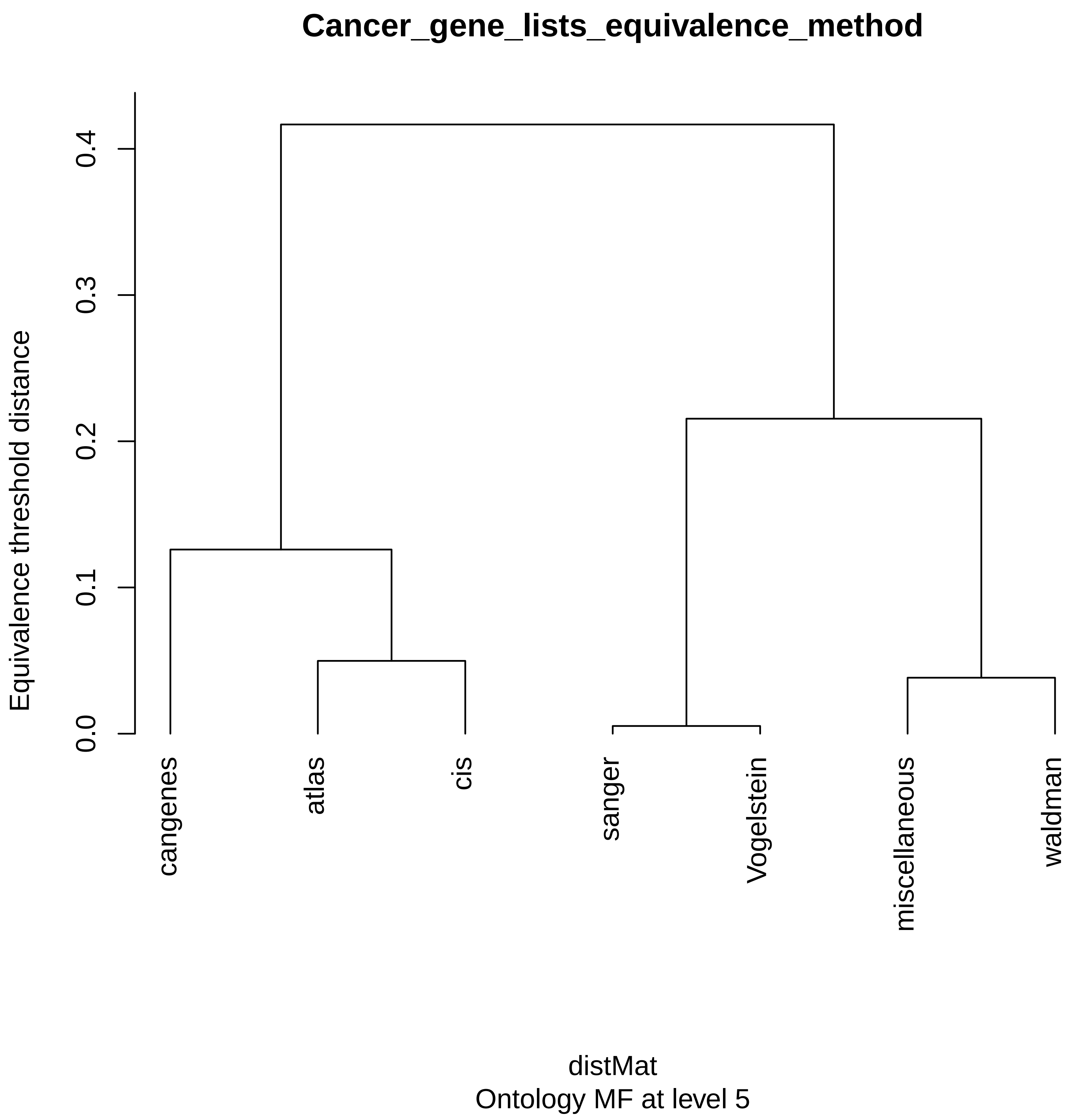
Cancer_gene_lists_equivalence_method
Equivalence threshold distance
0.0
0.1
0.2
0.3
0.4
cangenes
atlas
cis
sanger
Vogelstein
miscellaneous
waldman
distMat
Ontology MF at level 5

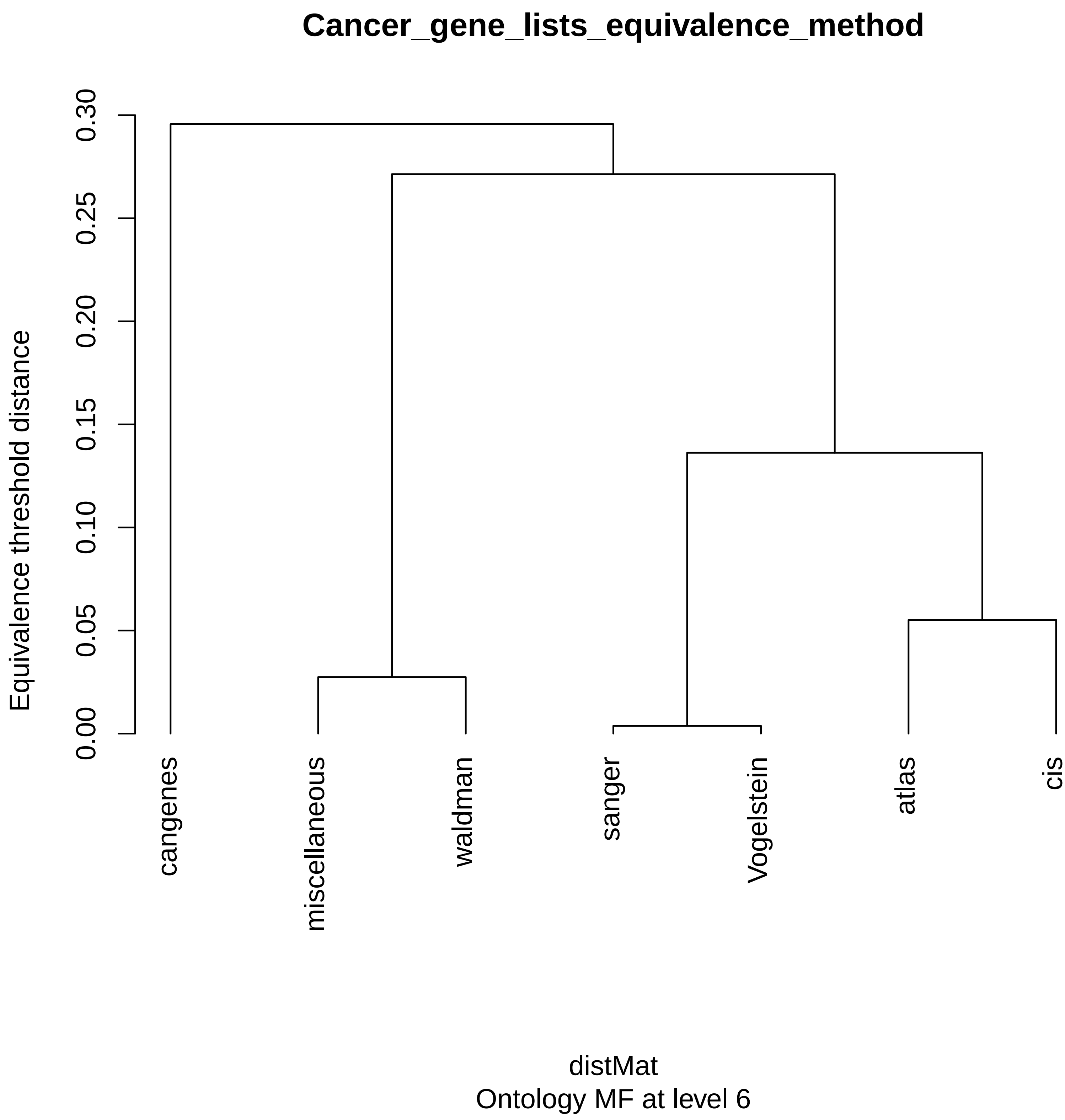
Cancer_gene_lists_equivalence_method
Equivalence threshold distance
0.00
0.05
0.10
0.15
0.20
0.25
0.30
cangenes
miscellaneous
waldman
sanger
Vogelstein
atlas
cis
distMat
Ontology MF at level 6

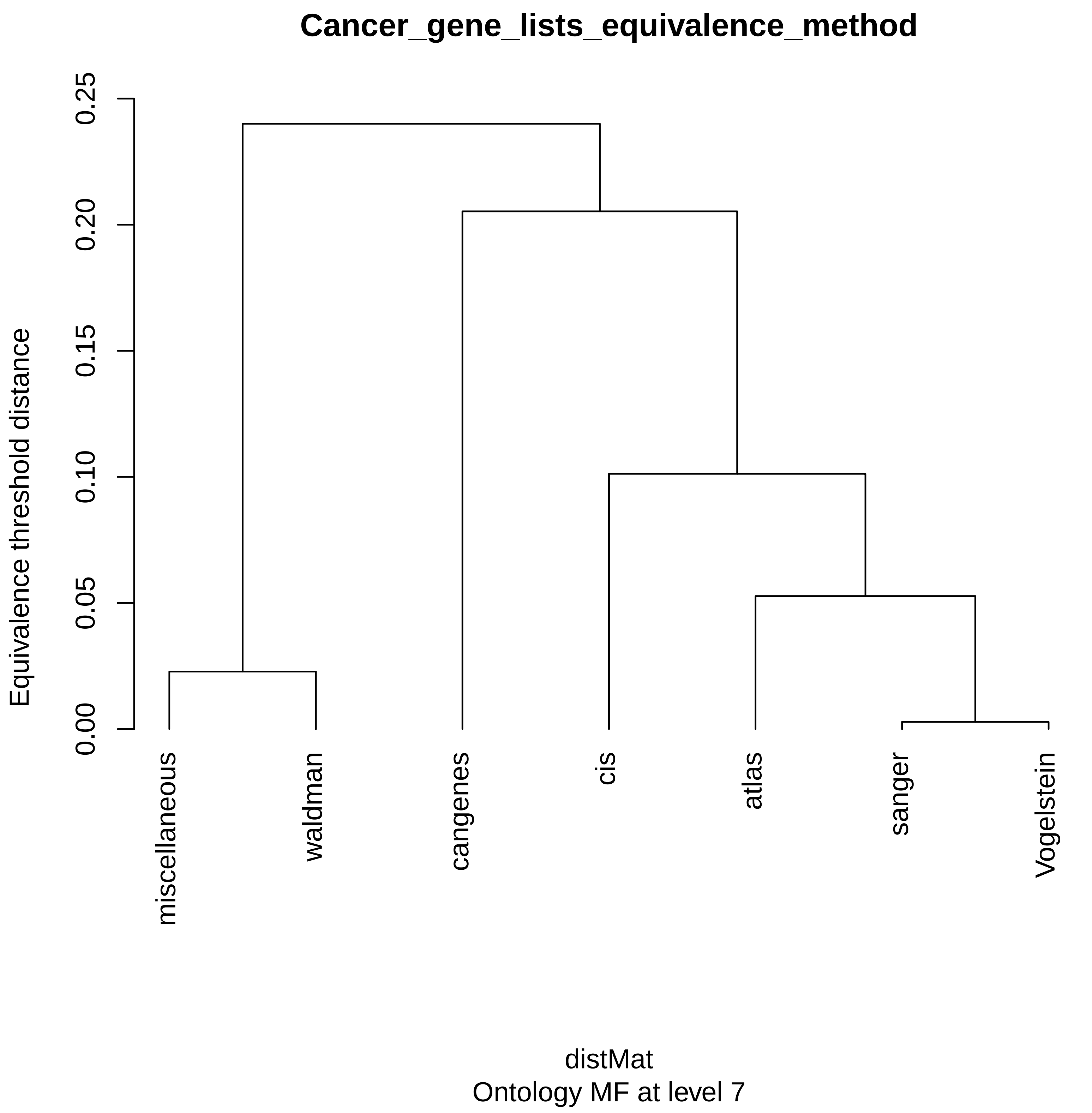
Cancer_gene_lists_equivalence_method
Equivalence threshold distance
0.00
0.05
0.10
0.15
0.20
0.25
miscellaneous
waldman
cangenes
cis
atlas
sanger
Vogelstein
distMat
Ontology MF at level 7

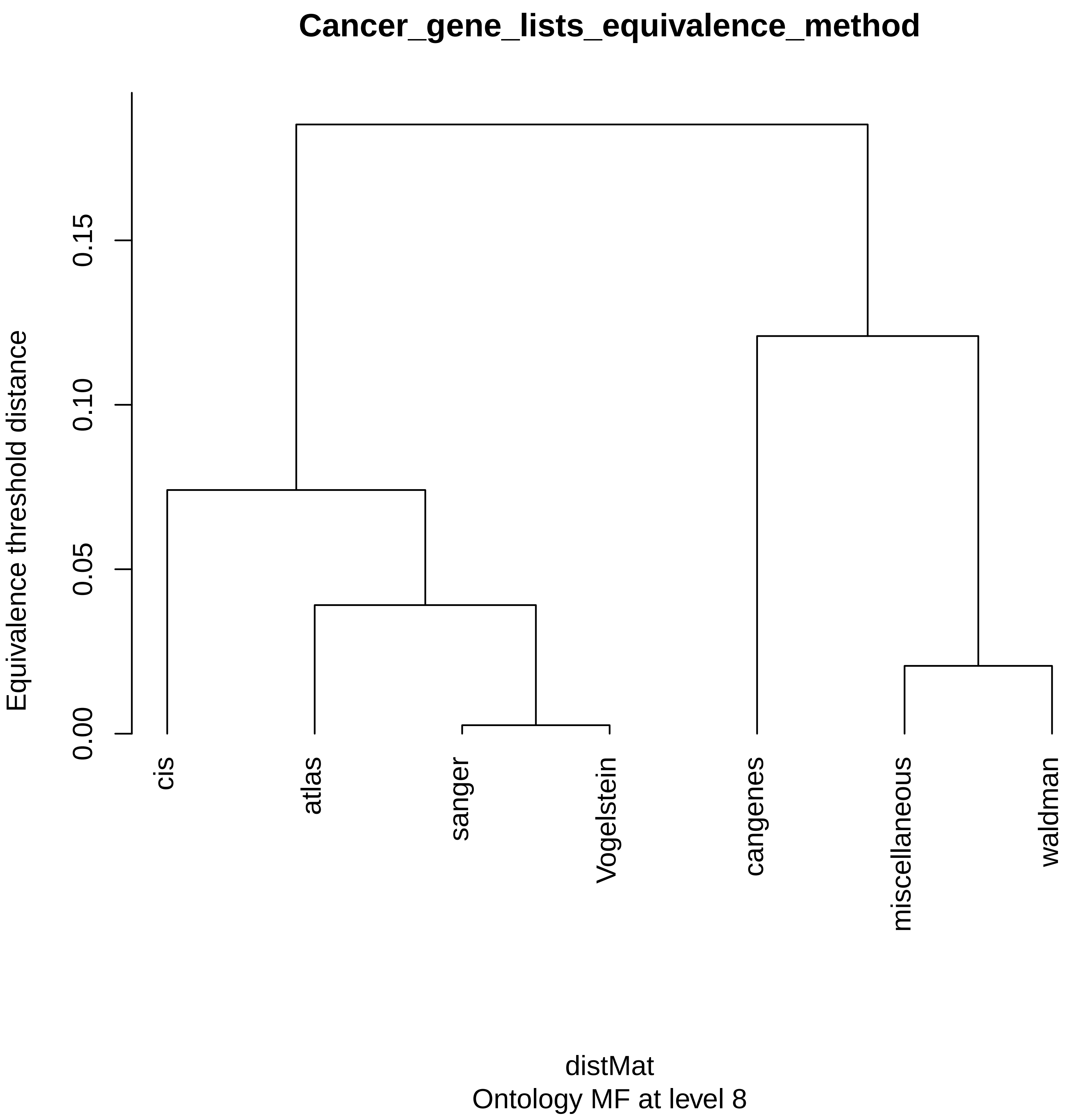

Cancer_gene_lists_equivalence_method
Equivalence threshold distance
0.00
0.05
0.10
0.15
cis
atlas
sanger
Vogelstein
cangenes
miscellaneous
waldman
distMat
Ontology MF at level 8

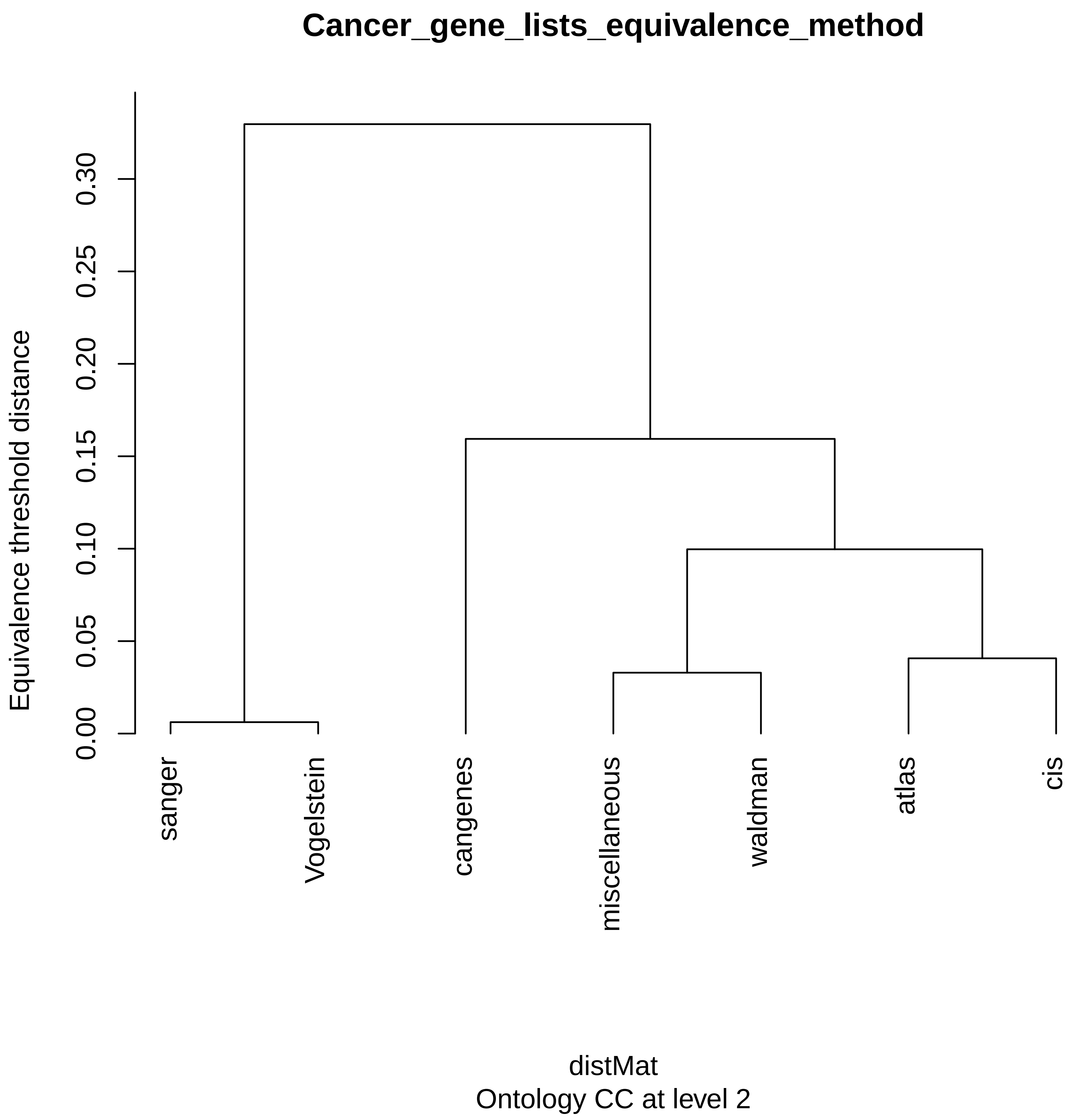
Cancer_gene_lists_equivalence_method
Equivalence threshold distance
0.00
0.05
0.10
0.15
0.20
0.25
0.30
sanger
Vogelstein
cangenes
miscellaneous
waldman
atlas
cis
distMat
Ontology CC at level 2

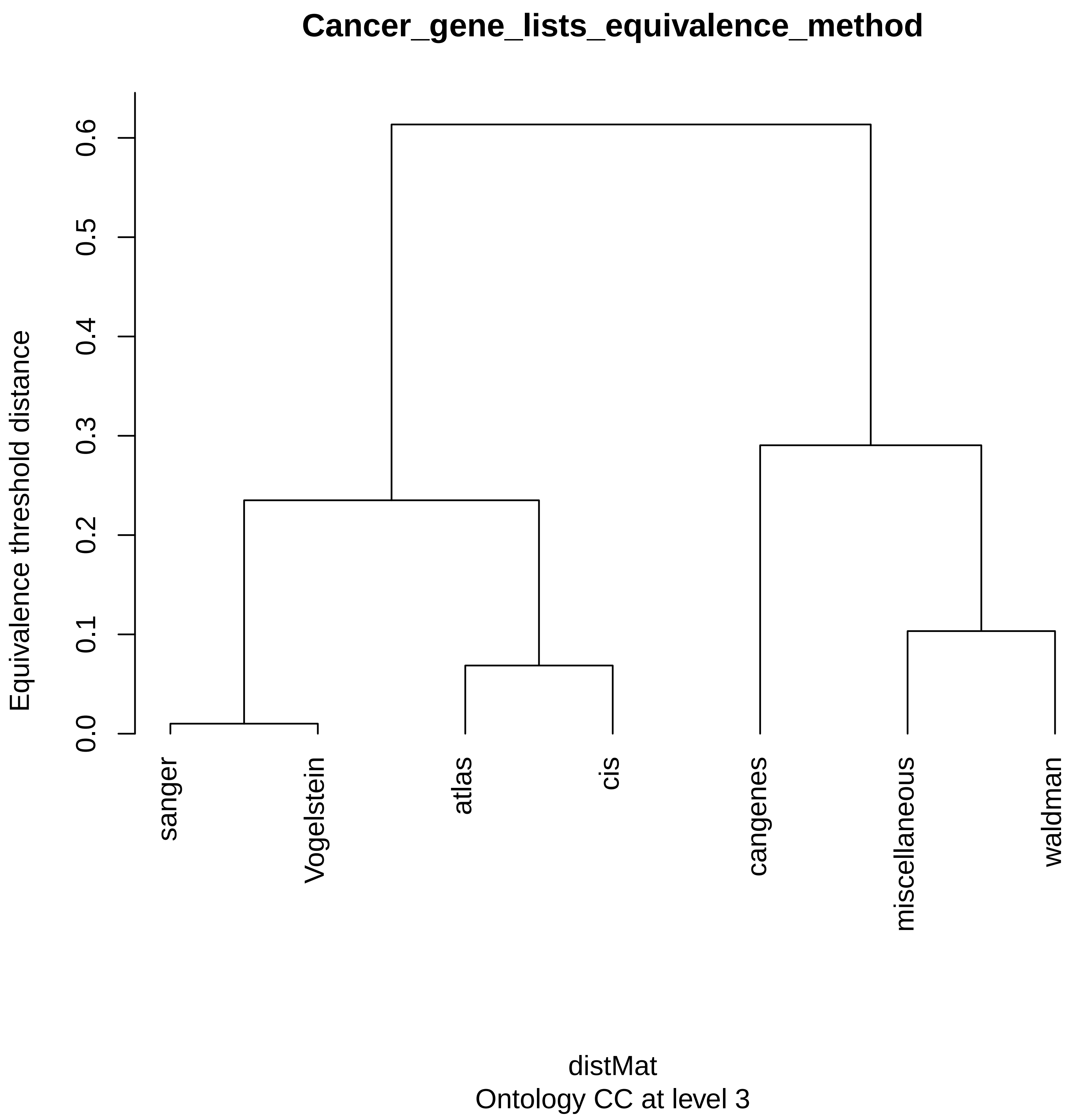
Cancer_gene_lists_equivalence_method
Equivalence threshold distance
0.0
0.1
0.2
0.3
0.4
0.5
0.6
sanger
Vogelstein
atlas
cis
cangenes
miscellaneous
waldman
distMat
Ontology CC at level 3

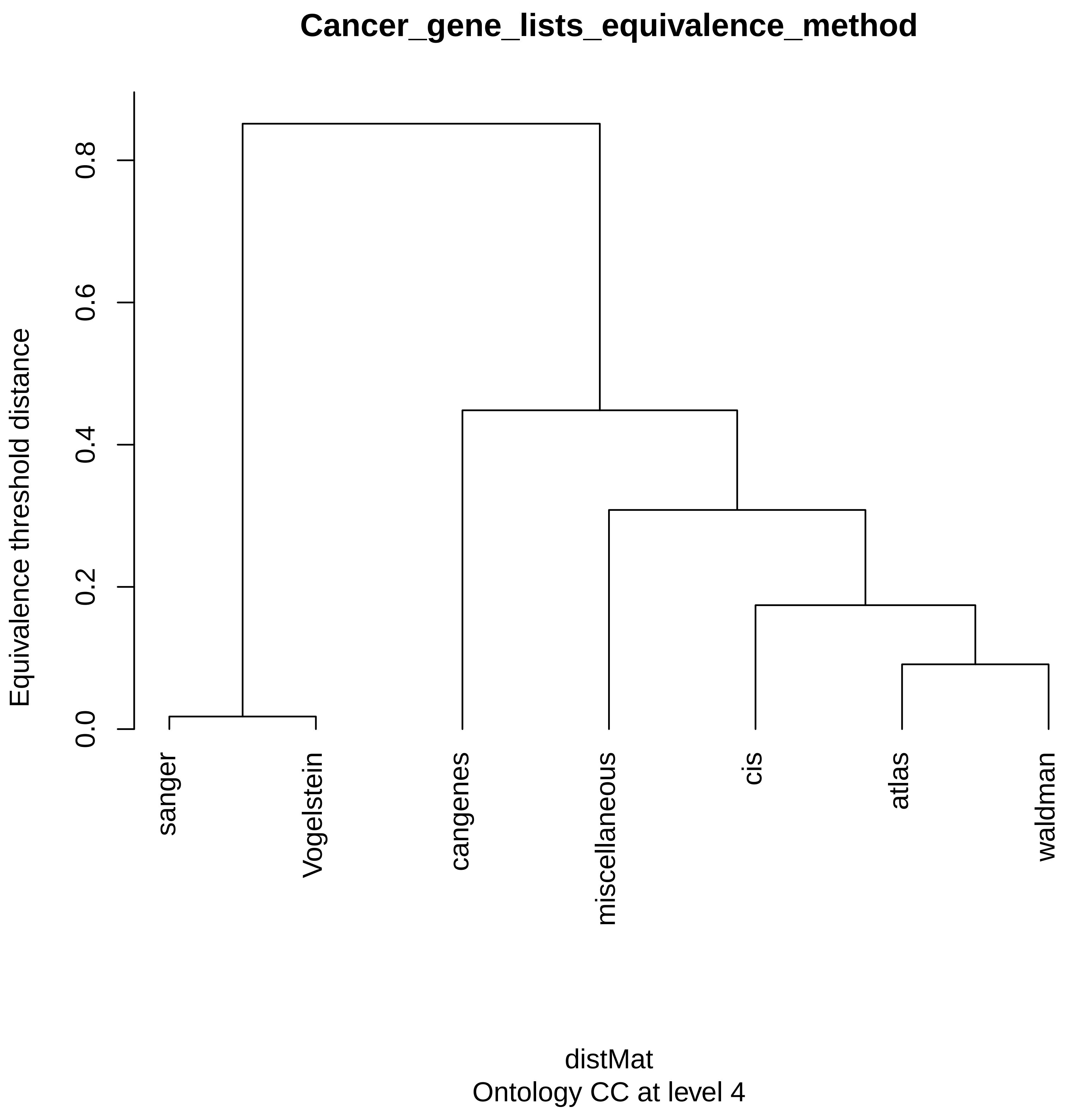
Cancer_gene_lists_equivalence_method
Equivalence threshold distance
0.0
0.2
0.4
0.6
0.8
sanger
Vogelstein
cangenes
miscellaneous
cis
atlas
waldman
distMat
Ontology CC at level 4

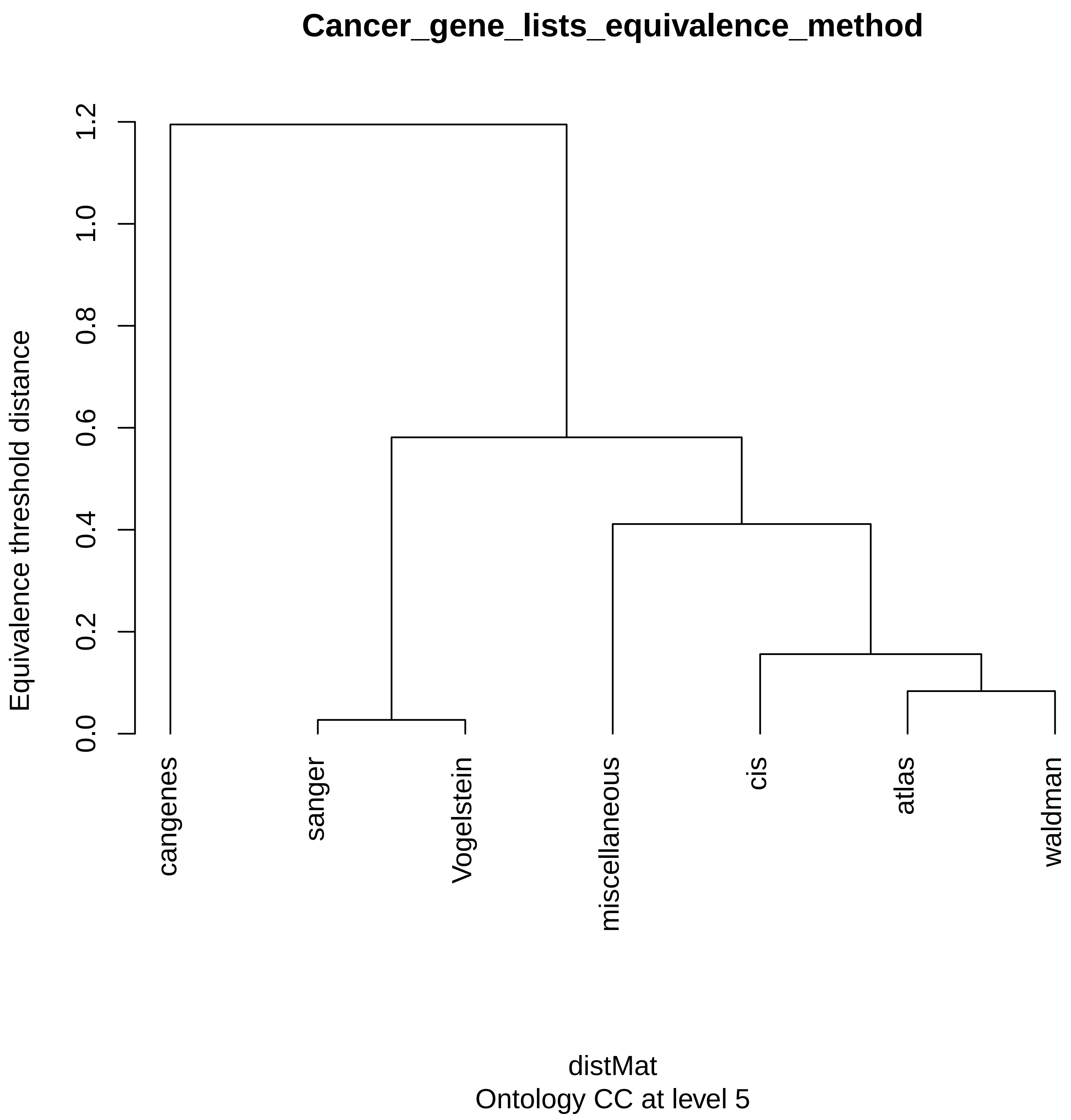

Cancer_gene_lists_equivalence_method
Equivalence threshold distance
0.0
0.2
0.4
0.6
0.8
1.0
1.2
cangenes
sanger
Vogelstein
miscellaneous
cis
atlas
waldman
distMat
Ontology CC at level 5

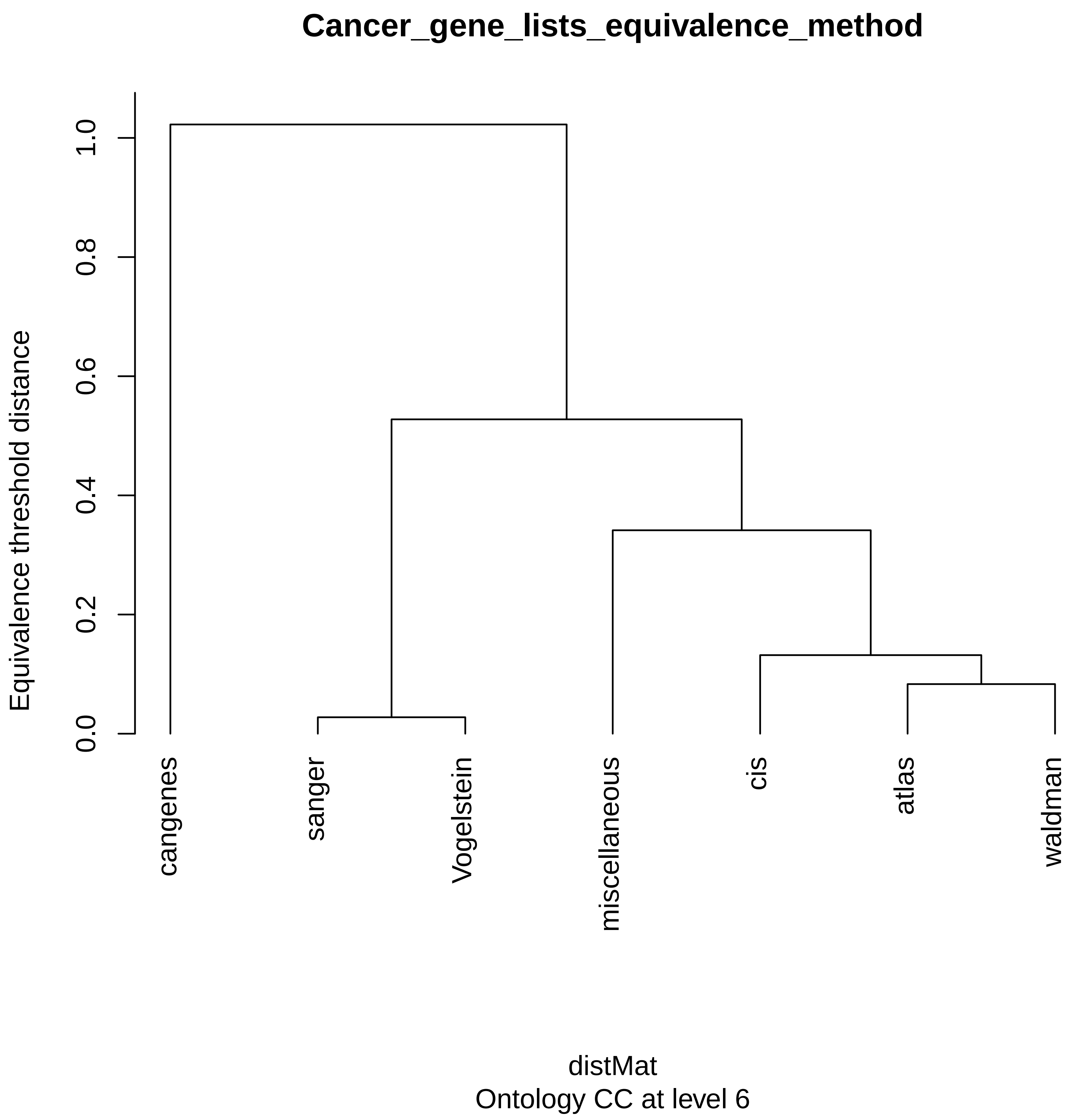
Cancer_gene_lists_equivalence_method
Equivalence threshold distance
1.0
0.8
0.6
0.4
0.2
0.0
cangenes
sanger
Vogelstein
miscellaneous
cis
atlas
waldman
distMat
Ontology CC at level 6

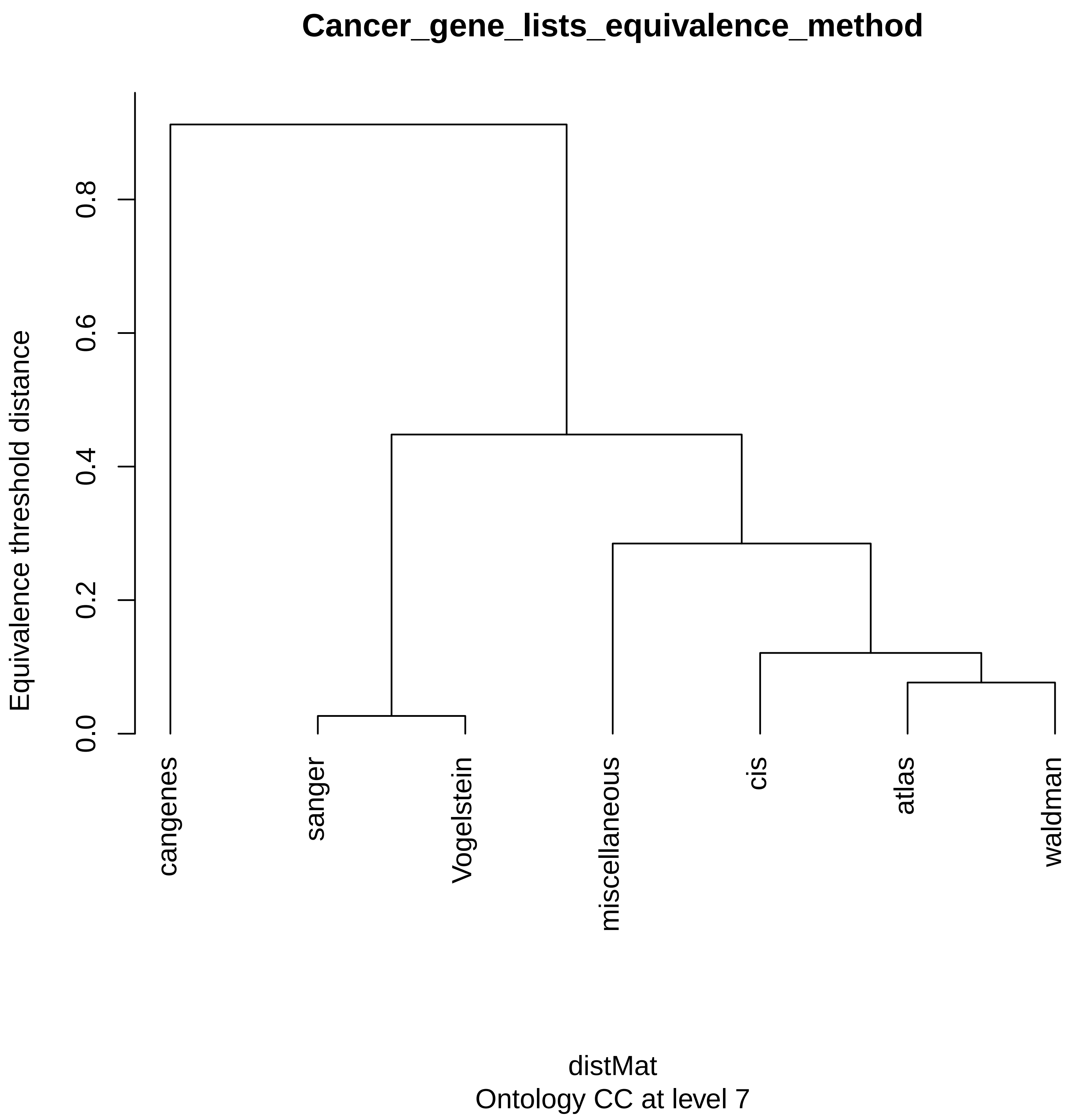

Cancer_gene_lists_equivalence_method
Equivalence threshold distance
0.0
0.2
0.4
0.6
0.8
cangenes
sanger
Vogelstein
miscellaneous
cis
atlas
waldman
distMat
Ontology CC at level 7

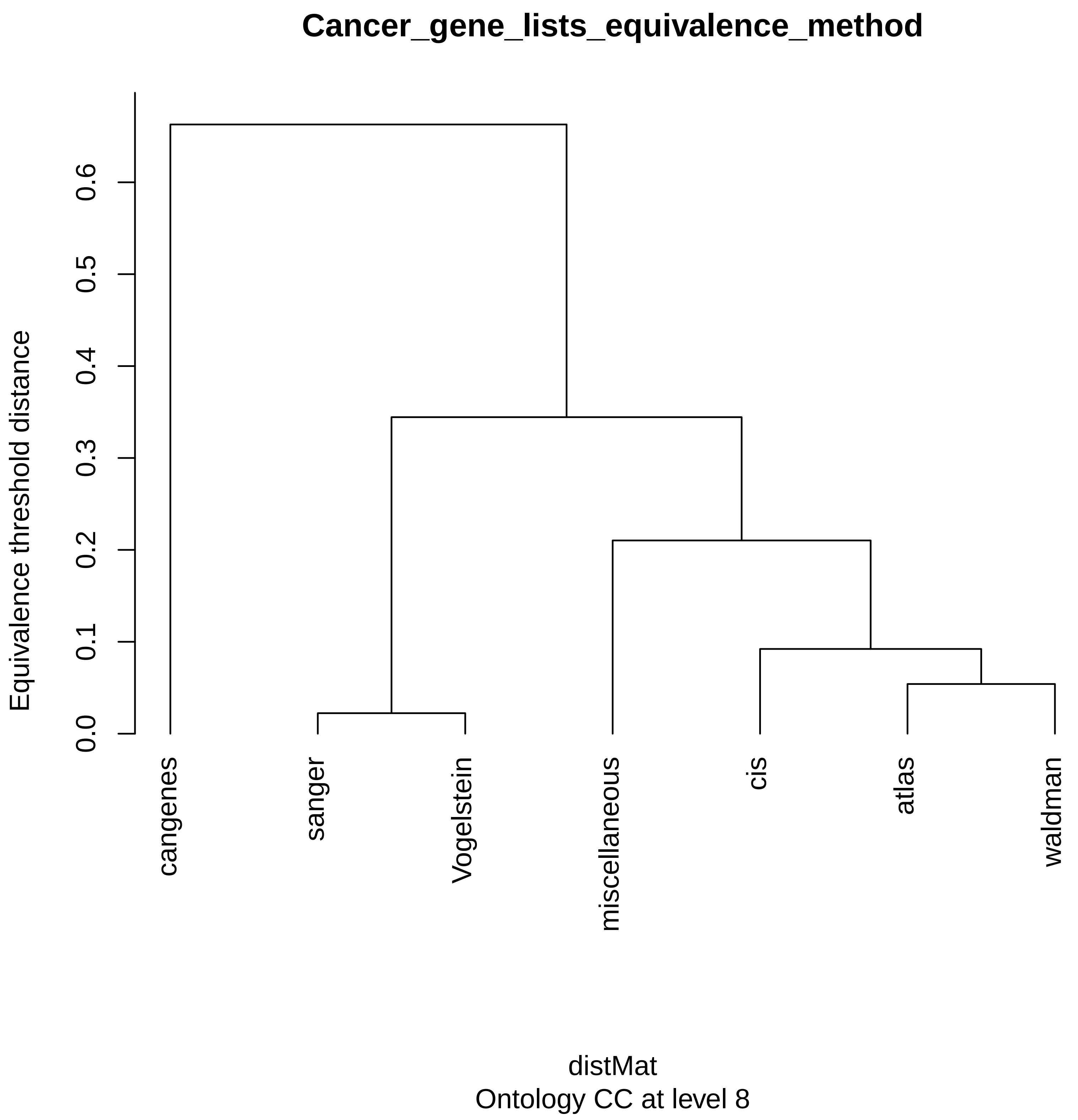
Cancer_gene_lists_equivalence_method
Equivalence threshold distance
0.0
0.1
0.2
0.3
0.4
0.5
0.6
cangenes
sanger
Vogelstein
miscellaneous
cis
atlas
waldman
distMat
Ontology CC at level 8

CANCER LISTS
Number of GO annotated genes for each list, ontology and GO level

GO level 2

	atlas	cangenes	cis	miscellaneous	sanger	Vogelstein	waldman
MF	957	173	559	185	424	409	410
BP	963	178	561	183	427	408	412
CC	974	183	567	185	430	412	413

GO level 3

	atlas	cangenes	cis	miscellaneous	sanger	Vogelstein	waldman
MF	938	168	546	182	421	404	400
BP	961	178	561	183	427	408	412
CC	966	182	566	184	428	410	412

GO level 4

	atlas	cangenes	cis	miscellaneous	sanger	Vogelstein	waldman
MF	921	166	513	181	412	396	403
BP	961	178	554	182	425	407	411
CC	966	181	566	184	428	410	412

GO level 5

	atlas	cangenes	cis	miscellaneous	sanger	Vogelstein	waldman
MF	892	161	496	181	394	378	403
BP	961	178	559	183	424	405	412
CC	947	179	558	173	425	406	398

GO level 6

	atlas	cangenes	cis	miscellaneous	sanger	Vogelstein	waldman
MF	799	139	442	171	351	340	375
BP	958	174	556	182	423	405	410
CC	929	167	543	171	419	401	394

GO level 7

	atlas	cangenes	cis	miscellaneous	sanger	Vogelstein	waldman
MF	620	100	336	136	281	273	303
BP	943	168	545	181	419	401	404
CC	908	161	524	168	416	399	381

GO level 8

	atlas	cangenes	cis	miscellaneous	sanger	Vogelstein	waldman
MF	475	76	269	115	234	227	250
BP	928	158	537	176	415	396	399
CC	901	157	520	167	413	397	376

GO level 9

	atlas	cangenes	cis	miscellaneous	sanger	Vogelstein	waldman
MF	390	63	219	92	206	207	193
BP	903	151	512	176	401	387	393
CC	739	117	405	134	352	341	302

GO level 10

	atlas	cangenes	cis	miscellaneous	sanger	Vogelstein	waldman
MF	178	37	106	33	111	112	89
BP	876	144	477	175	391	379	387
CC	708	112	384	121	339	329	282

GO level 11

	atlas	cangenes	cis	miscellaneous	sanger	Vogelstein	waldman
MF	53	14	21	5	35	30	15
BP	809	119	414	165	371	362	362
CC	641	101	351	112	321	313	265

GO level 12

	atlas	cangenes	cis	miscellaneous	sanger	Vogelstein	waldman
MF	12	3	4	0	4	4	6
BP	724	100	359	157	339	333	329
CC	386	64	187	65	191	197	150

GO level 13

	atlas	cangenes	cis	miscellaneous	sanger	Vogelstein	waldman
MF	1	0	2	0	0	0	2
BP	601	78	293	138	283	280	273
CC	247	40	114	42	135	142	90

GO level 14

	atlas	cangenes	cis	miscellaneous	sanger	Vogelstein	waldman
MF	0	0	2	0	0	0	1
BP	351	50	147	82	171	173	153
CC	128	15	43	25	84	86	44

GO level 15

	atlas	cangenes	cis	miscellaneous	sanger	Vogelstein	waldman
MF	0	0	1	0	0	0	0
BP	174	29	62	48	93	92	82
CC	42	2	10	8	23	24	14

GO level 16

	atlas	cangenes	cis	miscellaneous	sanger	Vogelstein	waldman
MF	0	0	0	0	0	0	0
BP	73	14	30	22	35	40	40
CC	13	1	3	2	1	1	0

Among other possibilities, semantic similarity methods are also a very interesting approach for analyzing the possible similarity between lists of genes. Despite many coincidences in the pattern of grouping, there is considerable variability among them, which is comparable to the variability among GO levels in the equivalence method. Here we display the resulting dendrograms (complete method as before) for all semantic similarity methods implemented in R package `GOSemSim` and for all three GO ontologies.

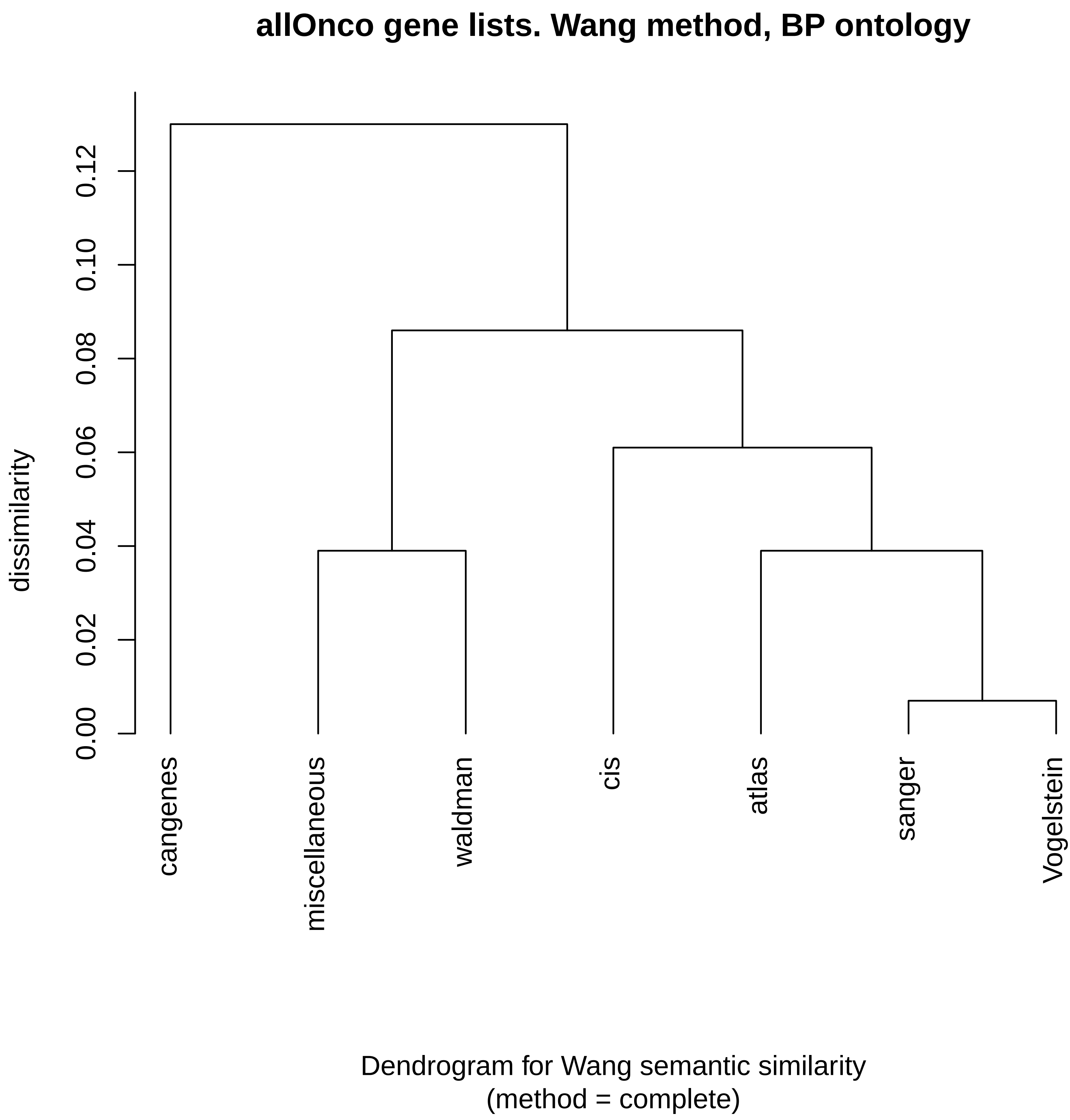
allOnco gene lists. Wang method, BP ontology
dissimilarity
0.00
0.02
0.04
0.06
0.08
0.10
0.12
cangenes
miscellaneous
waldman
cis
atlas
sanger
Vogelstein
Dendrogram for Wang semantic similarity
(method = complete)

allOnco gene lists. Resnik method, BP ontology

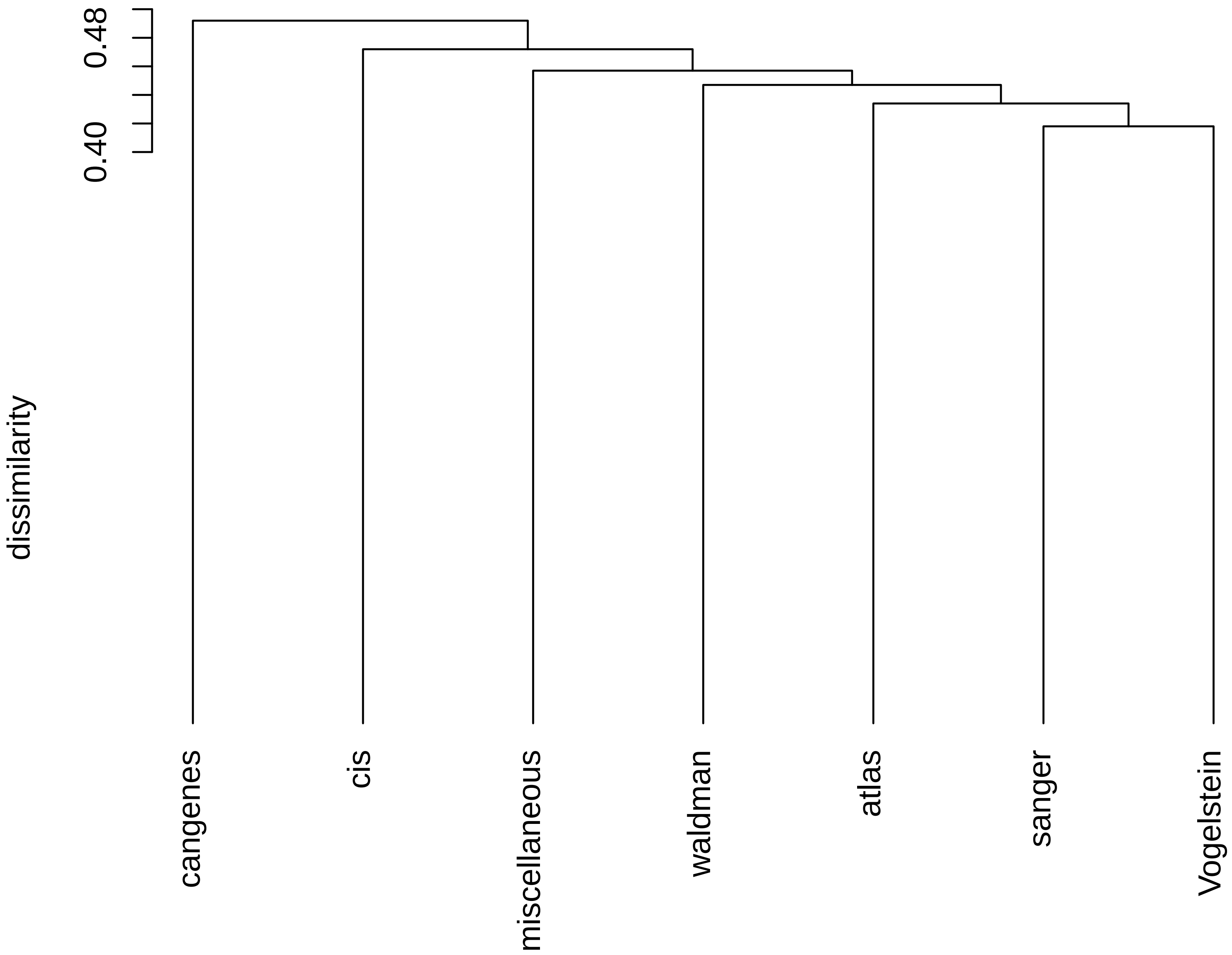

Dendrogram for Resnik semantic similarity
(method = complete)

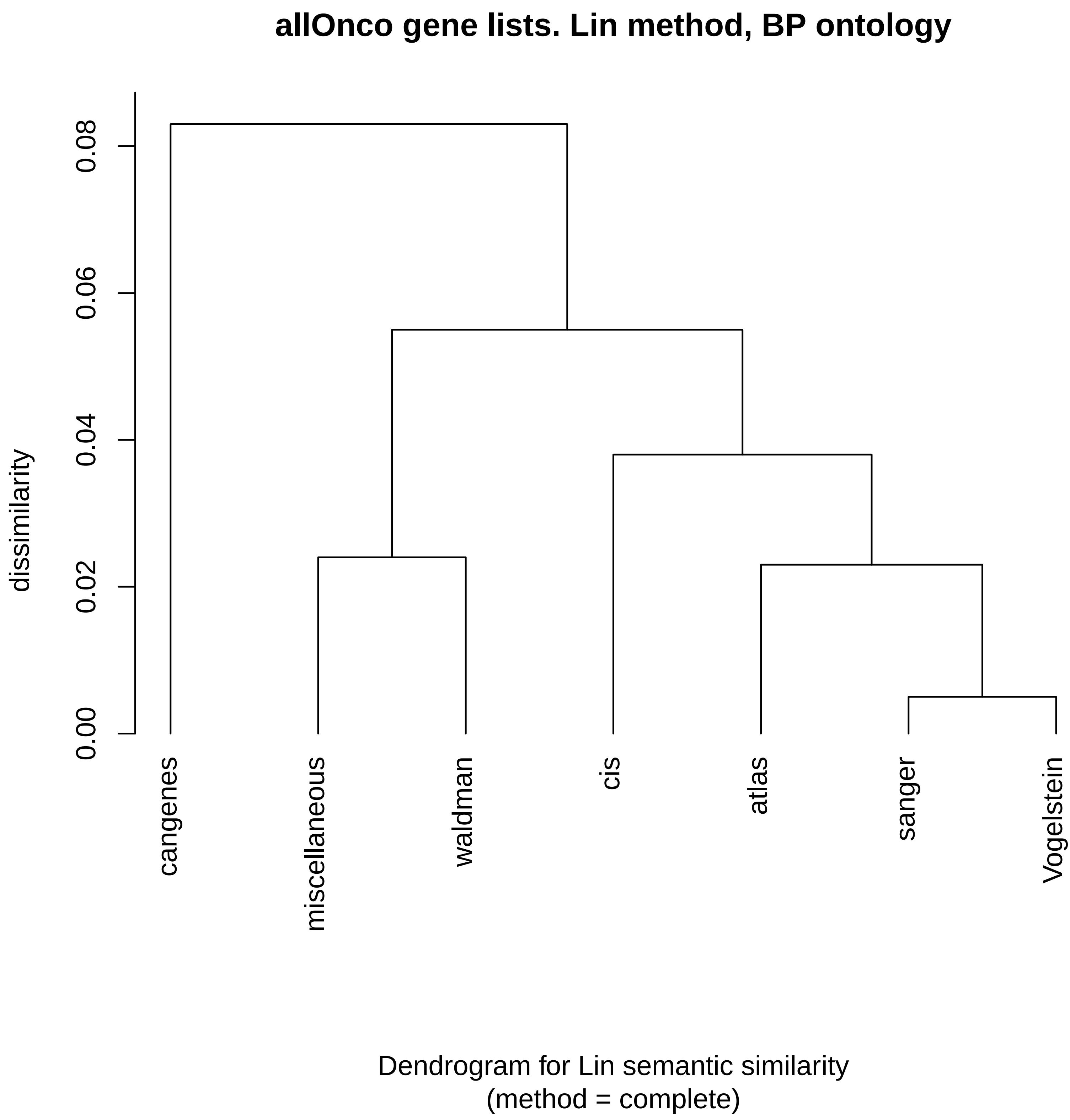
allOnco gene lists. Lin method, BP ontology
dissimilarity
0.00
0.02
0.04
0.06
0.08
cangenes
miscellaneous
waldman
cis
atlas
sanger
Vogelstein
Dendrogram for Lin semantic similarity
(method = complete)

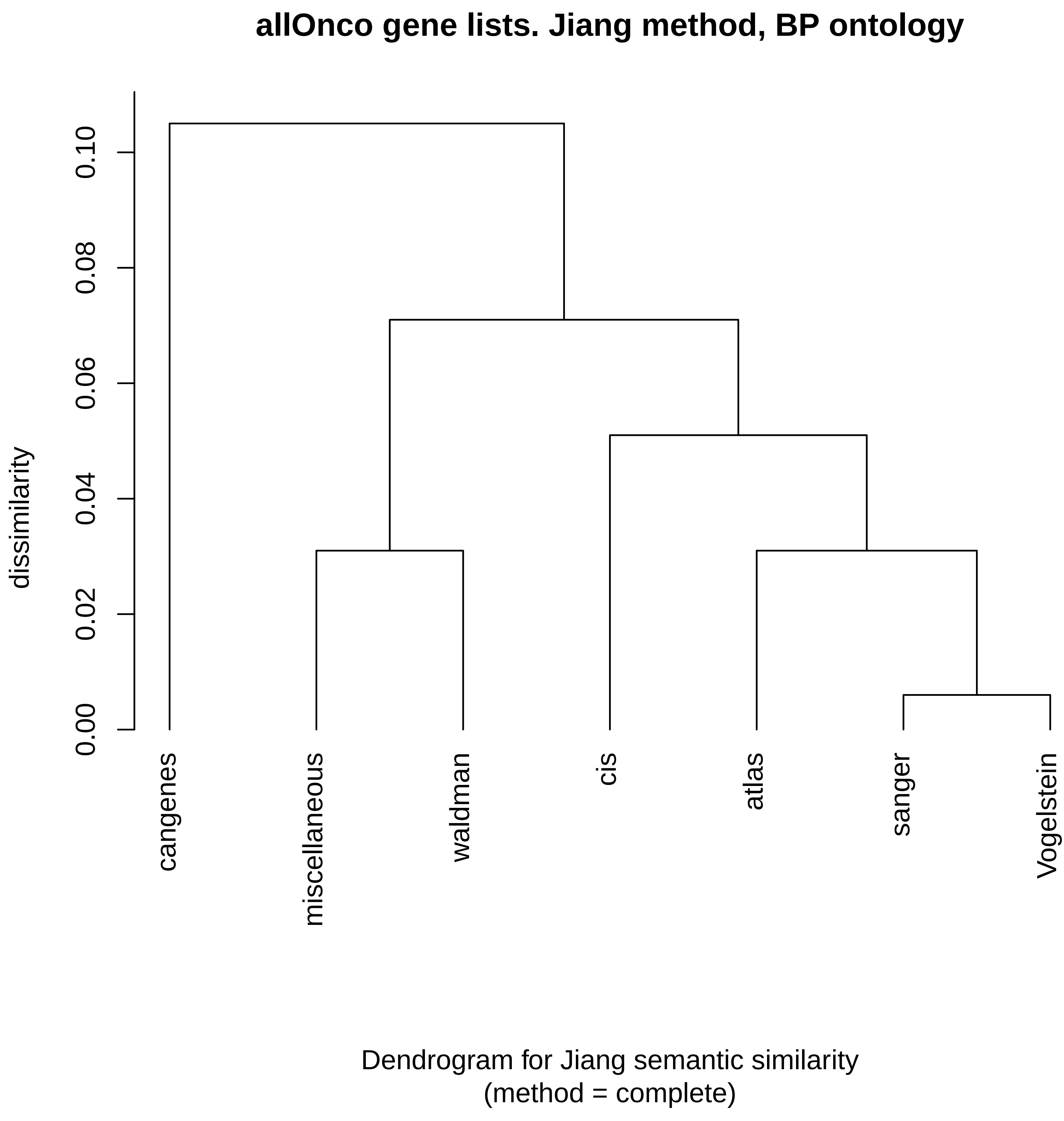
allOnco gene lists. Jiang method, BP ontology
dissimilarity
0.00
0.02
0.04
0.06
0.08
0.10
cangenes
miscellaneous
waldman
cis
atlas
sanger
Vogelstein
Dendrogram for Jiang semantic similarity
(method = complete)

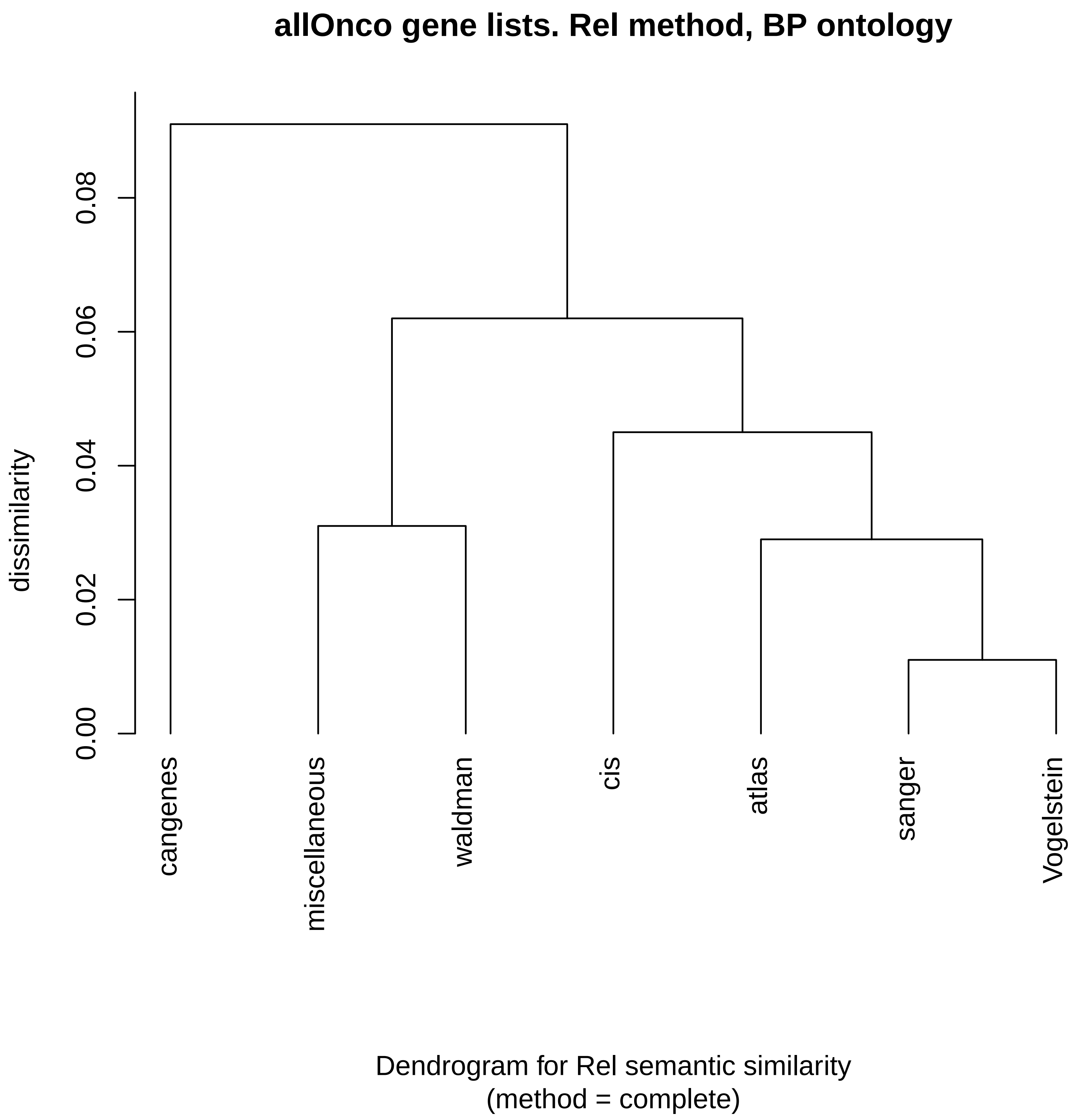
allOnco gene lists. Rel method, BP ontology
dissimilarity
0.00
0.02
0.04
0.06
0.08
cangenes
miscellaneous
waldman
cis
atlas
sanger
Vogelstein
Dendrogram for Rel semantic similarity
(method = complete)

allOnco gene lists. Wang method, MF ontology

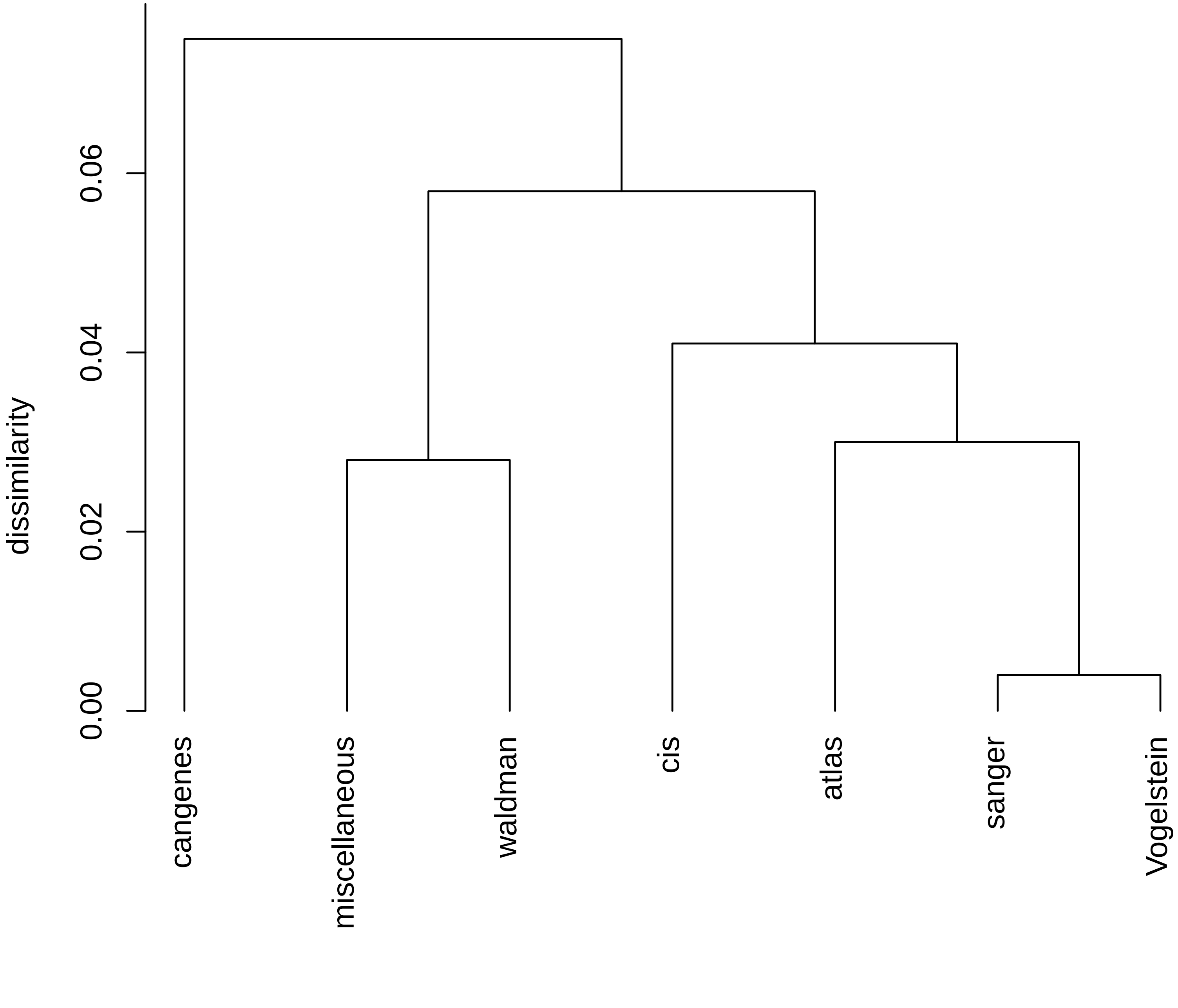

Dendrogram for Wang semantic similarity
(method = complete)

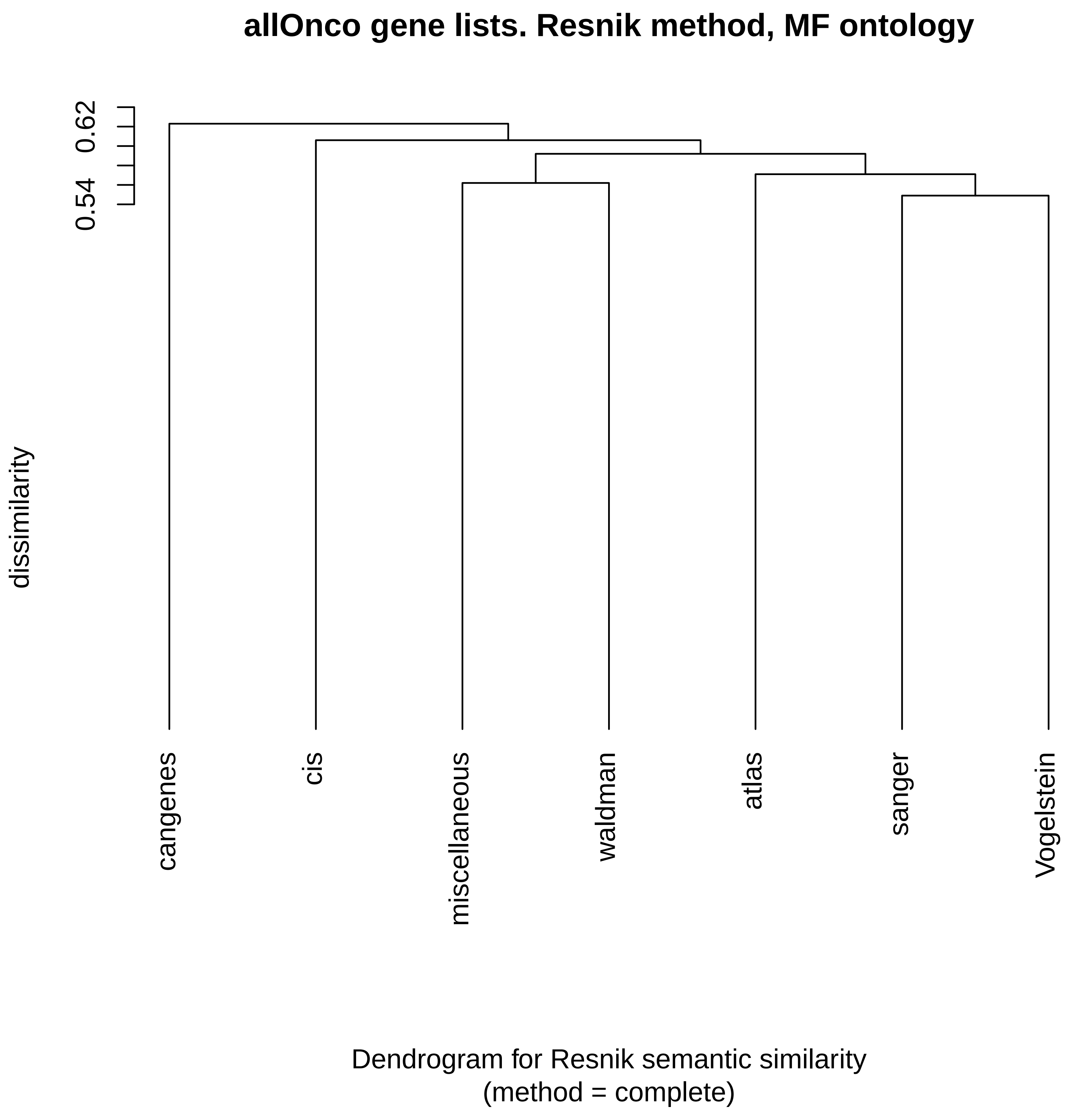
allOnco gene lists. Resnik method, MF ontology
0.62
0.54
dissimilarity
cangenes
cis
miscellaneous
waldman
atlas
sanger
Vogelstein
Dendrogram for Resnik semantic similarity
(method = complete)

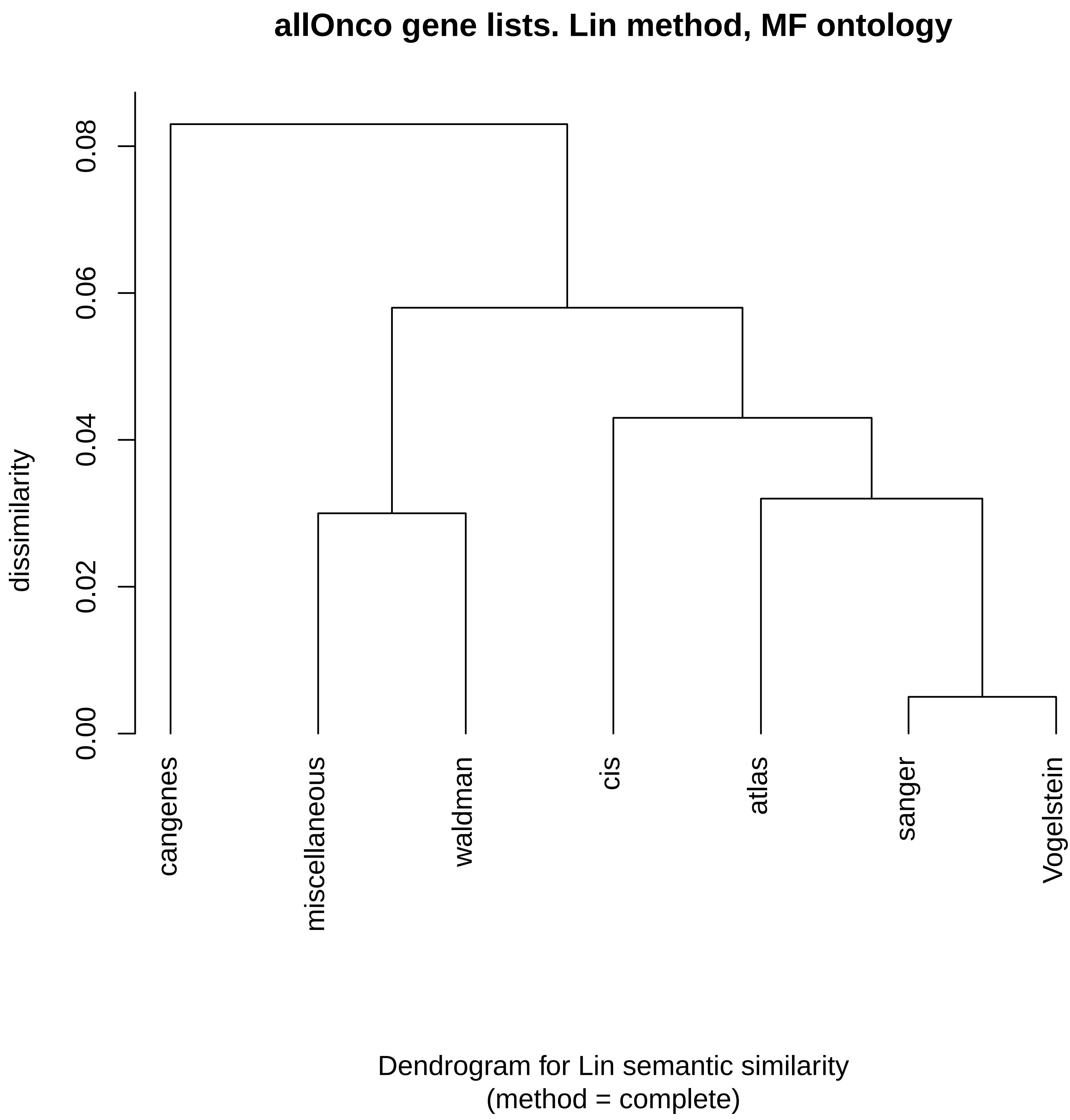
allOnco gene lists. Lin method, MF ontology
dissimilarity
0.00
0.02
0.04
0.06
0.08
cangenes
miscellaneous
waldman
cis
atlas
sanger
Vogelstein
Dendrogram for Lin semantic similarity
(method = complete)

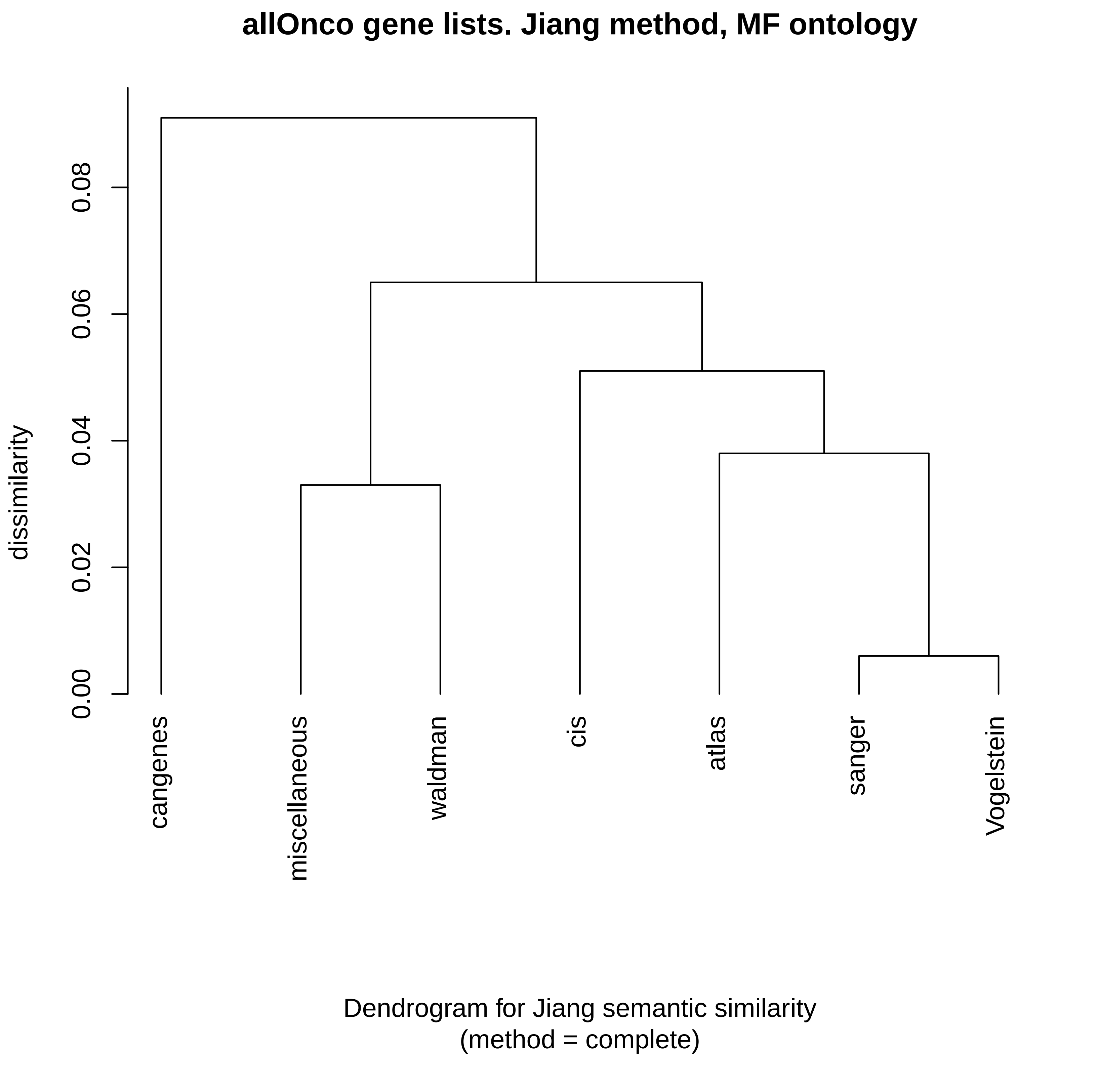
allOnco gene lists. Jiang method, MF ontology
dissimilarity
0.00
0.02
0.04
0.06
0.08
cangenes
miscellaneous
waldman
cis
atlas
sanger
Vogelstein
Dendrogram for Jiang semantic similarity
(method = complete)

allOnco gene lists. Rel method, MF ontology

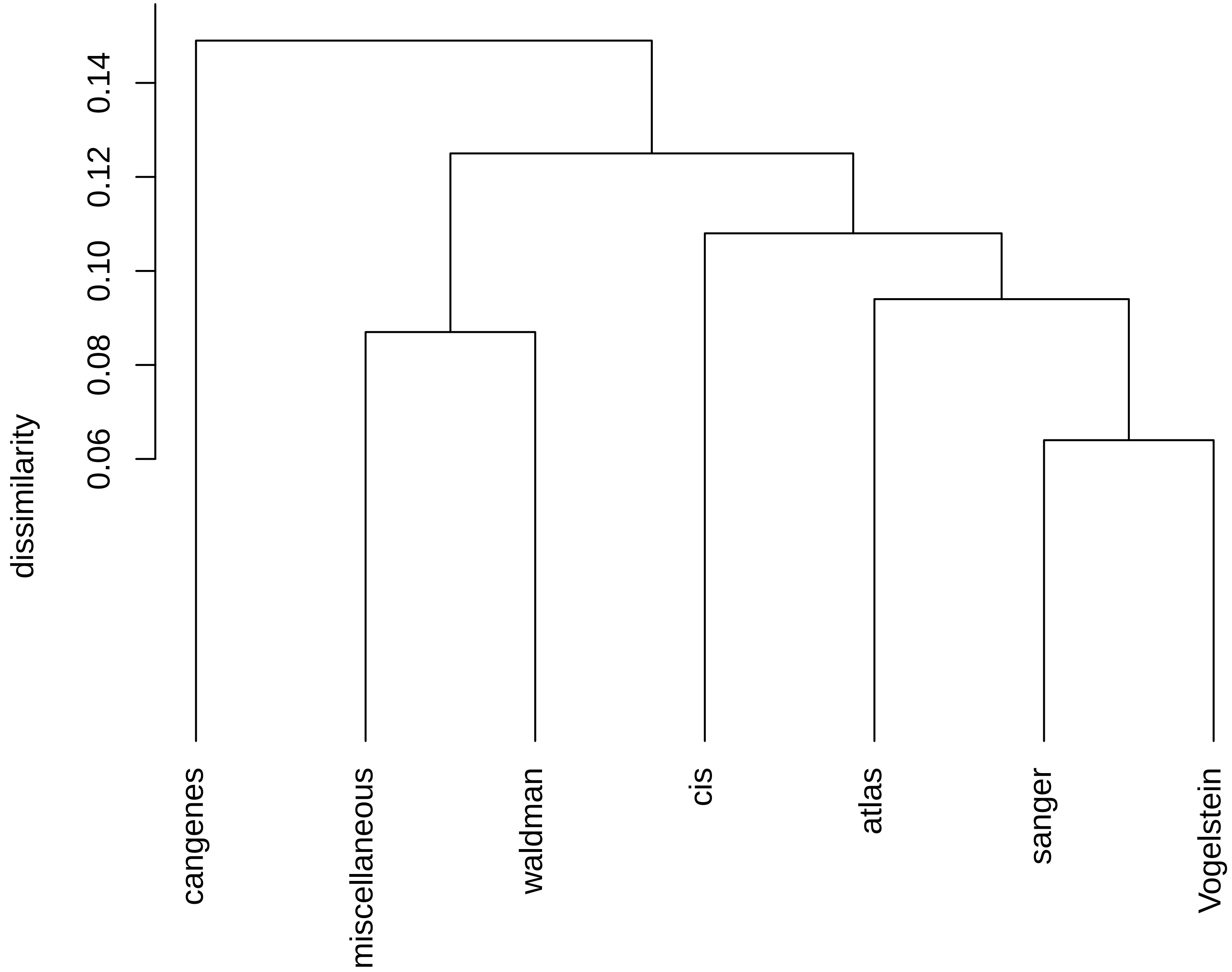

Dendrogram for Rel semantic similarity
(method = complete)

allOnco gene lists. Wang method, CC ontology

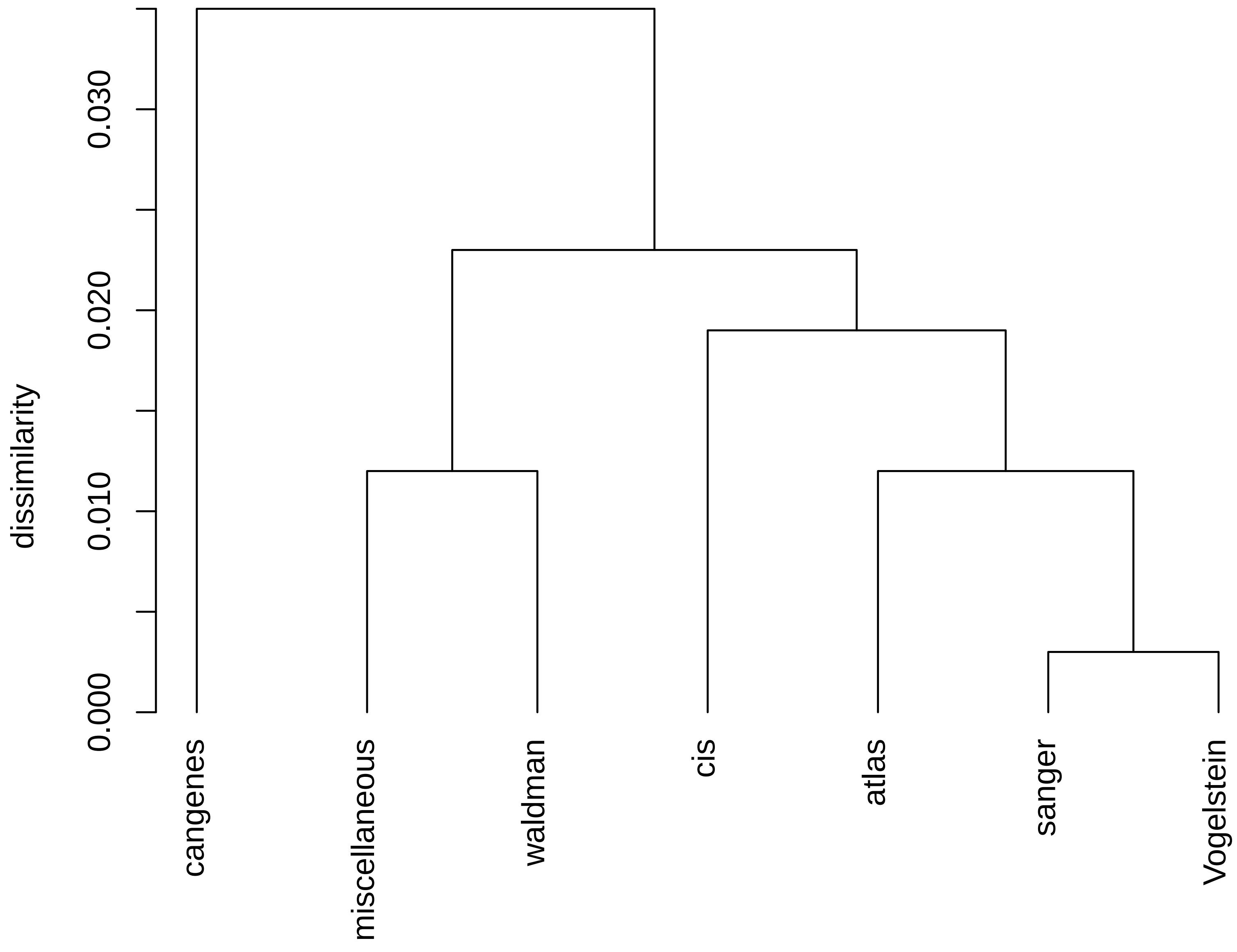

Dendrogram for Wang semantic similarity
(method = complete)

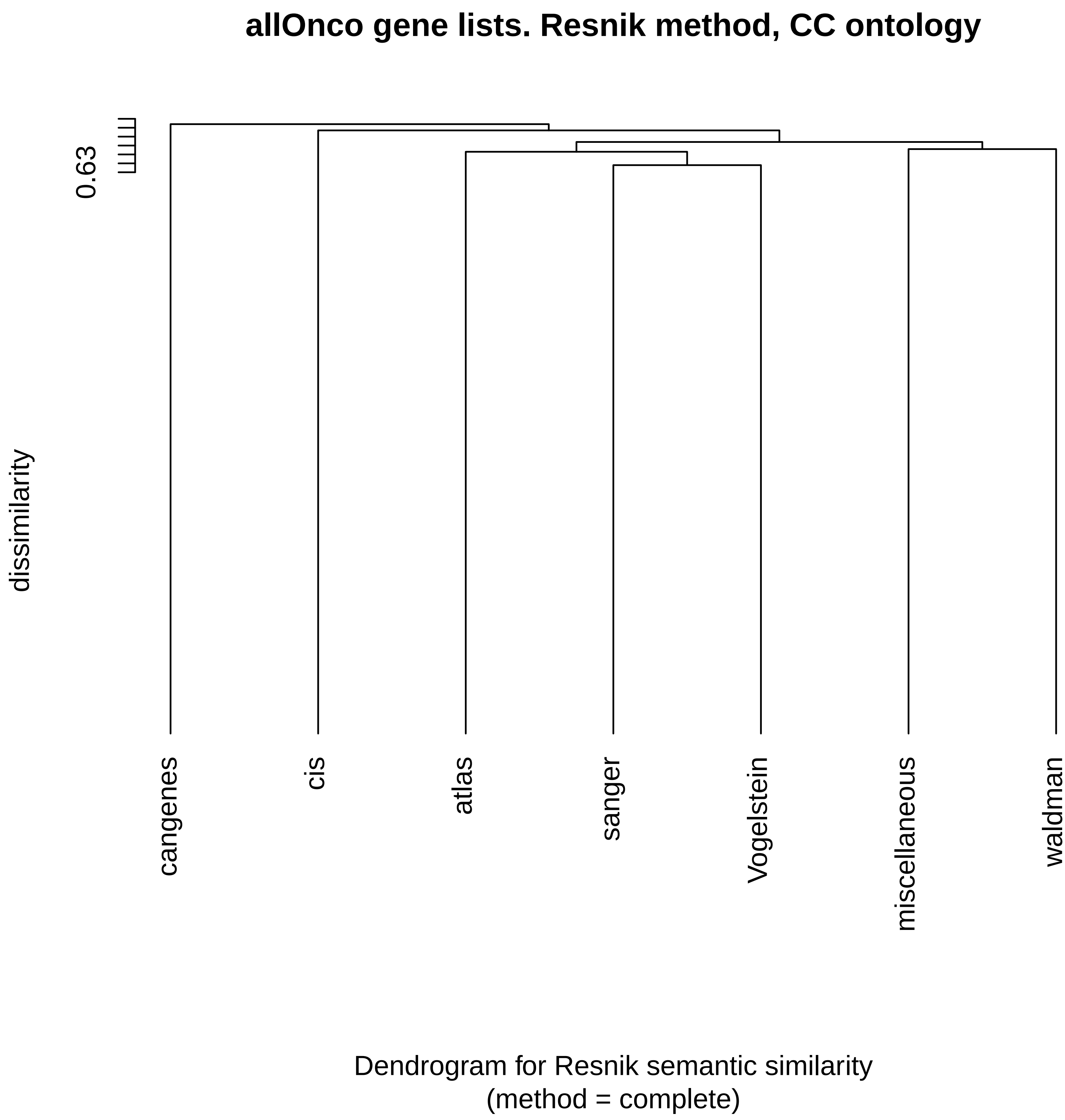
allOnco gene lists. Resnik method, CC ontology
0.63
dissimilarity
cangenes
cis
atlas
sanger
Vogelstein
miscellaneous
waldman
Dendrogram for Resnik semantic similarity
(method = complete)

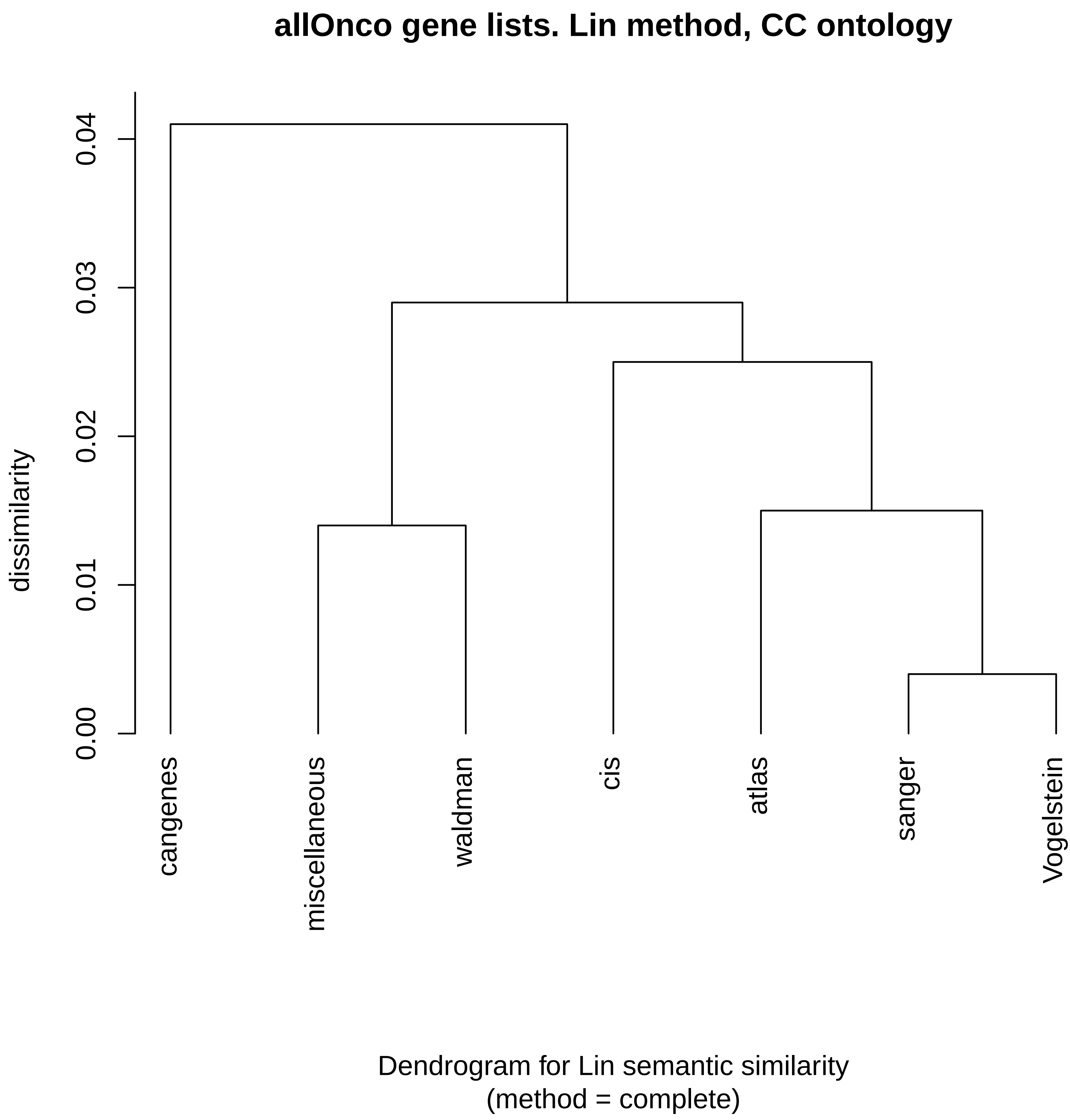
allOnco gene lists. Lin method, CC ontology
dissimilarity
0.00
0.01
0.02
0.03
0.04
cangenes
miscellaneous
waldman
cis
atlas
sanger
Vogelstein
Dendrogram for Lin semantic similarity
(method = complete)

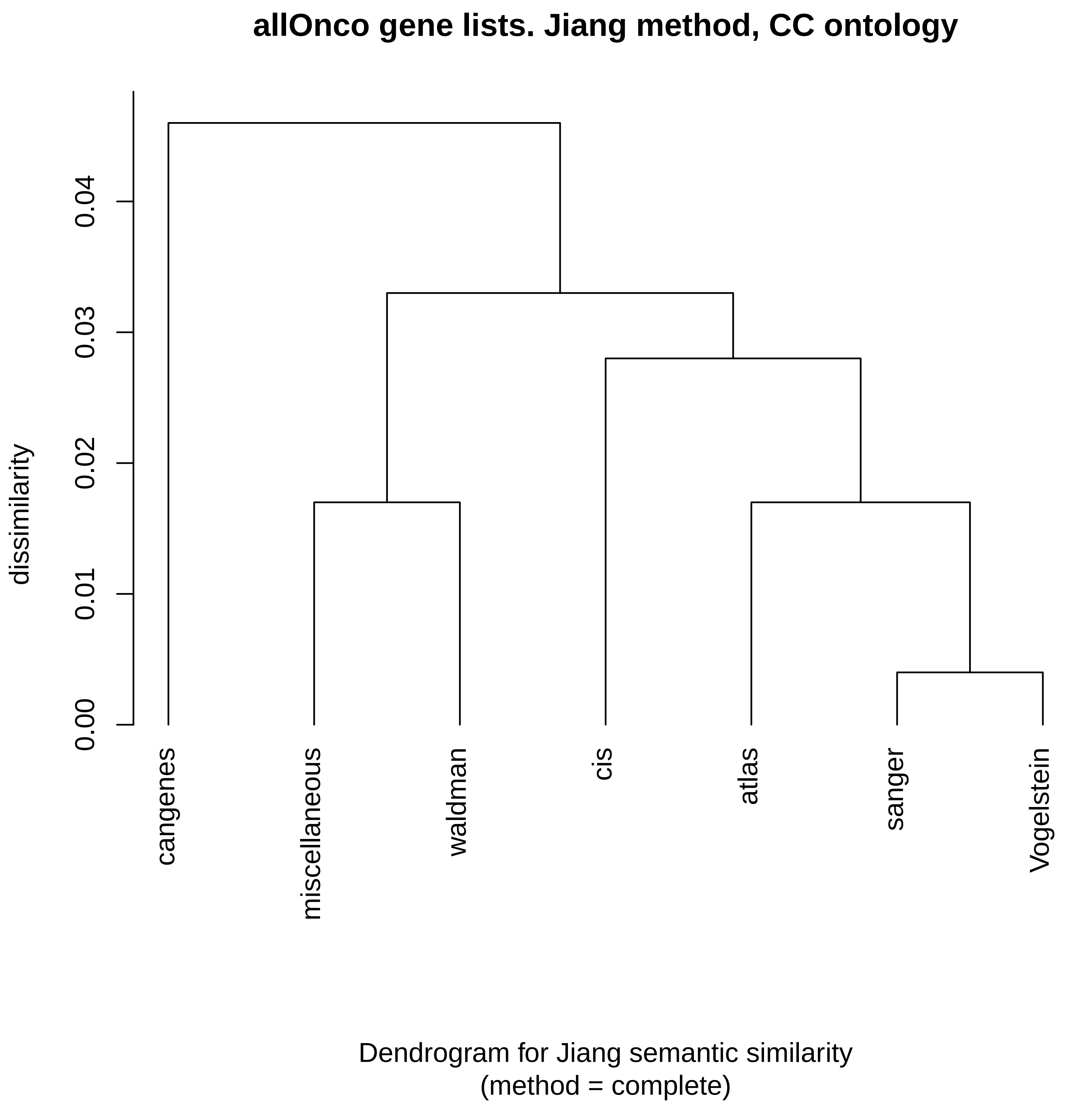
allOnco gene lists. Jiang method, CC ontology
dissimilarity
0.00
0.01
0.02
0.03
0.04
cangenes
miscellaneous
waldman
cis
atlas
sanger
Vogelstein
Dendrogram for Jiang semantic similarity
(method = complete)

allOnco gene lists. Rel method, CC ontology

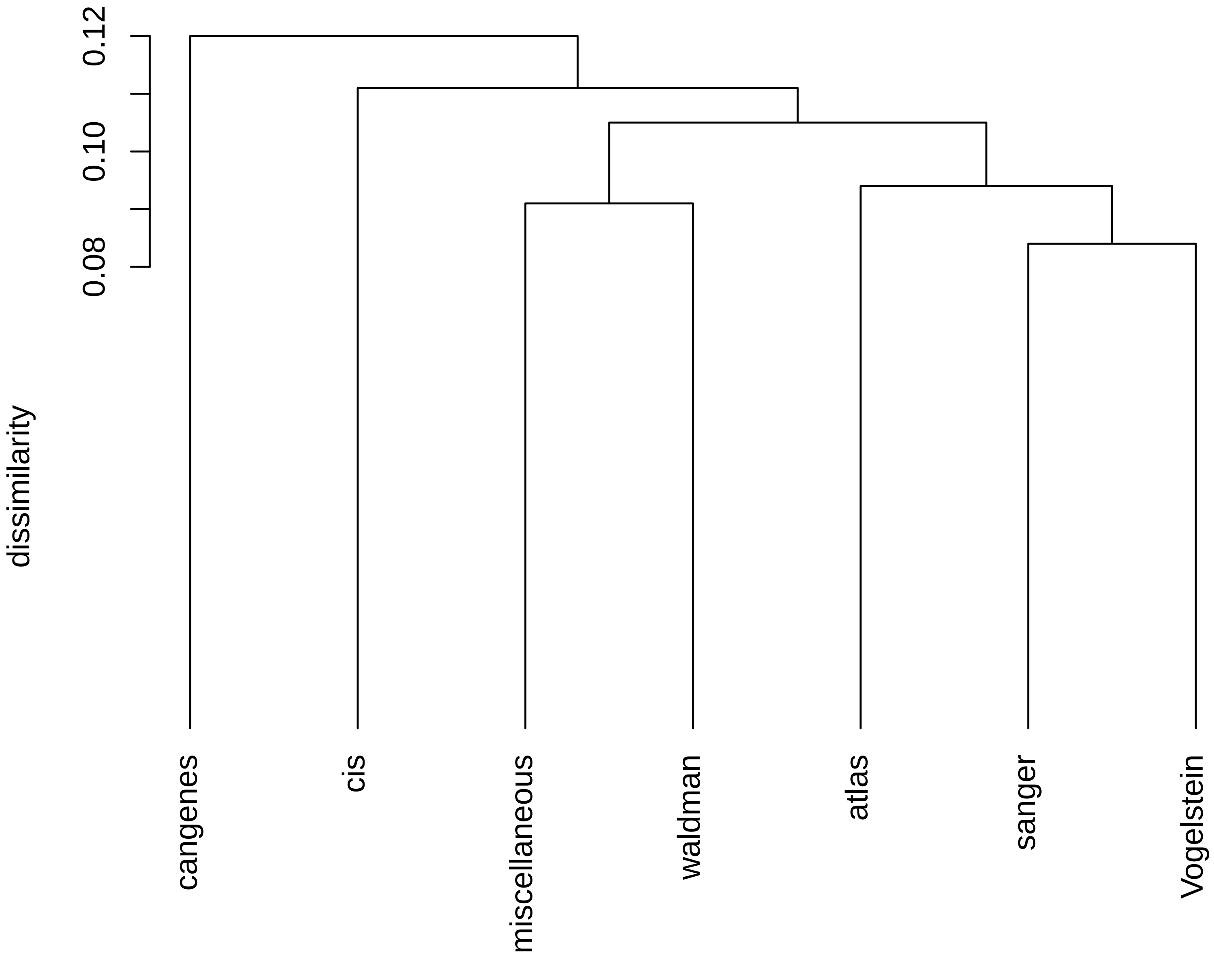

Dendrogram for Rel semantic similarity
(method = complete)
