# Supplementary material for: An equivalence approach to the integrative analysis of feature lists
Source: BMC Bioinformatics. 2019 Aug 27;20:441. doi: 10.1186/s12859-019-3008-x (PMC6712676; doi:10.1186/s12859-019-3008-x)

Figure 1: False negatives (in blue) and positives (in red), as a function of some values of the true squared Euclidean distance d. Balanced case of two gene lists of size 200 with 20 genes in common. Equivalence limit set at $\Delta = 0.25$. The null hypothesis of the equivalence test states that the true squared Euclidean distance, d, is greater than or equal to Δ, that is to say, that both lists are sufficiently dissimilar according to the Δ limit criterion. Thus, rejecting this hypothesis corresponds to declaring equivalence. When the true simulated distance is $d < \Delta$, not rejecting the null hypothesis (not declaring equivalence) corresponds to a false negative. Its probability is computed as $1 - Pr\{Reject H_0\}$. When $d \geq \Delta$, declaring equivalence is a false positive. Its probability is directly $Pr\{Reject H_0\}$.

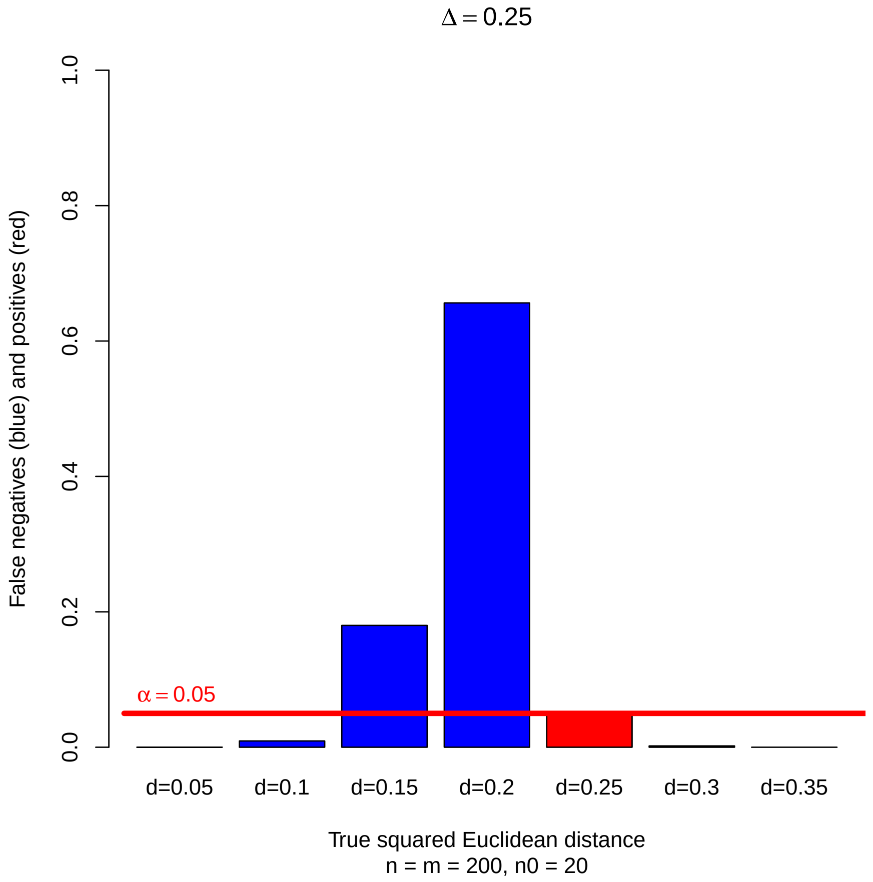

Figure 2: False negatives (in blue) and positives (in red), at different scenarios as a function of the true squared Euclidean distance. Balanced case of two gene lists of size 1000 with 100 genes in common. Equivalence limit at $\Delta = 0.25$. The null hypothesis of the equivalence test states that the true squared Euclidean distance, d, is greater than or equal to Δ, that is to say, that both lists are sufficiently dissimilar according to the Δ limit criterion. Thus, rejecting this hypothesis corresponds to declaring equivalence. When the true simulated distance is $d < \Delta$, not rejecting the null hypothesis (not declaring equivalence) corresponds to a false negative. Its probability is computed as $1 - Pr\{Reject H_0\}$. When $d \geq \Delta$, declaring equivalence is a false positive. Its probability is directly $Pr\{Reject H_0\}$.

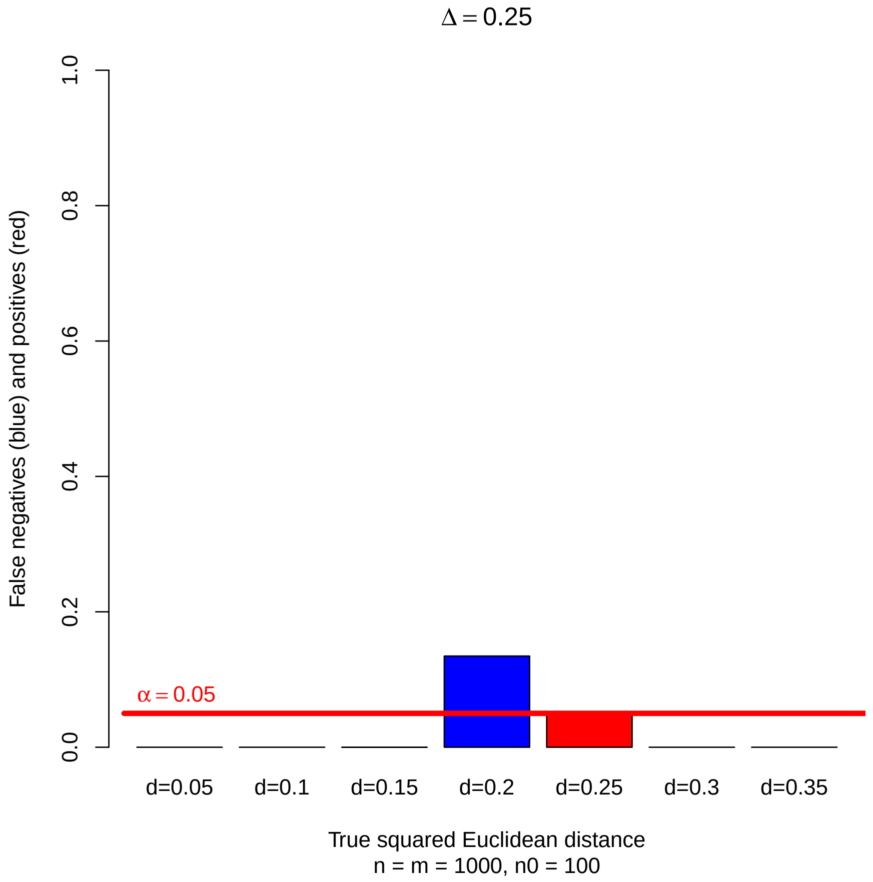

Figure 3: False negatives (in blue) and positives (in red), at different scenarios as a function of the true squared Euclidean distance. Balanced case of two gene lists of size 200 with 20 genes in common. Equivalence limit at $\Delta = 0.025$. The null hypothesis of the equivalence test states that the true squared Euclidean distance, d, is greater than or equal to Δ, that is to say, that both lists are sufficiently dissimilar according to the Δ limit criterion. Thus, rejecting this hypothesis corresponds to declaring equivalence. When the true simulated distance is $d < \Delta$, not rejecting the null hypothesis (not declaring equivalence) corresponds to a false negative. Its probability is computed as $1 - Pr\{Reject H_0\}$. When $d \geq \Delta$, declaring equivalence is a false positive. Its probability is directly $Pr\{Reject H_0\}$.

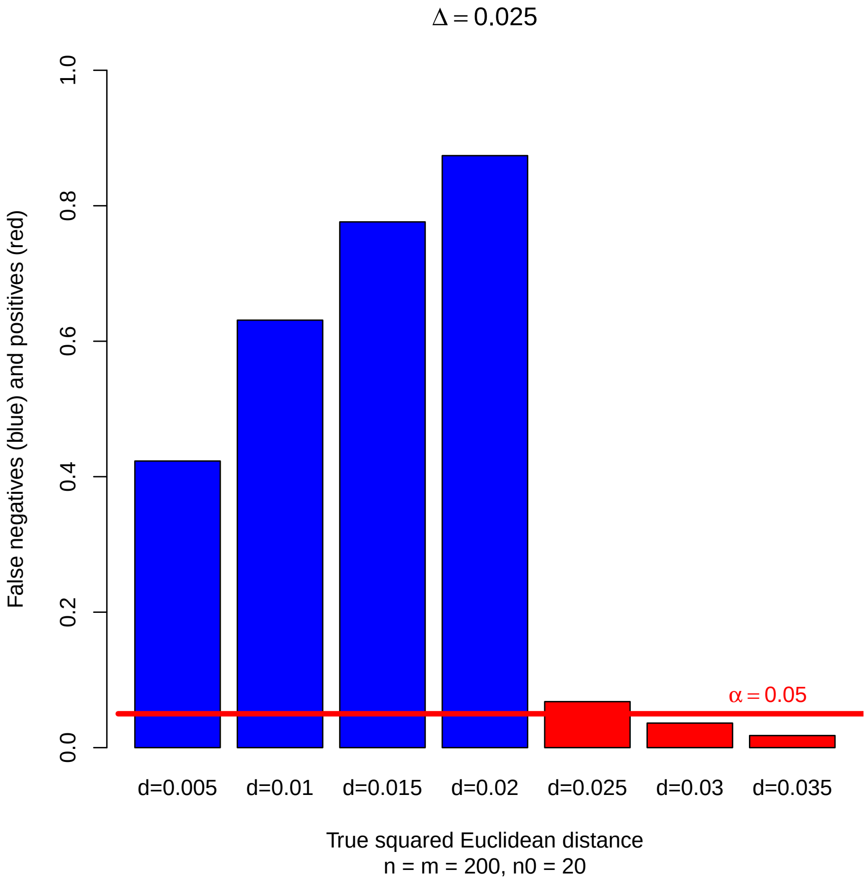

Supplement: Supplementary file 5 — The simulation study performed shows the ROC curves but a reviewer suggested that depicting False Positive and False Negative rates could also be interesting. This file shows three plots with FN (in blue) and FP (in red), as a function of some values of the true squared Euclidean distance. The plots differ in the total number of genes and the number of genes in common between the three lists. (PDF 30 kb) [file 12859_2019_3008_MOESM5_ESM.pdf]
