# Supplementary material for: An equivalence approach to the integrative analysis of feature lists
Source: BMC Bioinformatics. 2019 Aug 27;20:441. doi: 10.1186/s12859-019-3008-x (PMC6712676; doi:10.1186/s12859-019-3008-x)
Supplement: Supplementary file 6 — Comparison between the equivalence test with a standard test of positive dependency suggested by a reviewer. (PDF 348 kb) [file 12859_2019_3008_MOESM6_ESM.pdf]

SUPPLEMENTARY DISCUSSION ON EQUIVALENCE vs. STANDARD SIGNIFICANCE TESTING

One of the reviewers of the paper suggested that we should compare our equivalence test with an alternative approach. He/She suggested that a “standard test” can be built by:

- First, look for enriched categories in each gene list and then
- Compute, using a Fisher test, a similarity measure based in the number of common enriched and non-enriched categories among the lists.

All the proposed methodology in our paper is based on the “Functional (GO) profile” concept and on calculating a dissimilarity index between these profiles, more specifically (but not necessarily excluding other indexes) on the squared Euclidean distance. In order to make our approach easily comparable with that suggestion, both profiles being compared may be represented as follows:

	Enriched in both lists			Enriched only in list 1			Enriched only in list 2			Non-enriched in both lists		
GO item	I_1	…	I_a	I_{a+1}	…	I_{a+b}	I_{a+b+1}	…	I_{a+b+c}	$I_{a+b+c+1}$	…	$I_{a+b+c+d}$
Annotation freq. in gene list 1	F_{11}	…	F_{1a}	$F_{1,a+1}$	…	$F_{1,a+b}$	$F_{1,a+b+1}$	…	$F_{1,a+b+c}$	$F_{1,a+b+c+1}$	…	$F_{1,a+b+c+d}$
Annotation freq. in gene list 2	F_{21}	…	F_{2a}	$F_{2,a+1}$	…	$F_{2,a+b}$	$F_{2,a+b+1}$	…	$F_{2,a+b+c}$	$F_{2,a+b+c+1}$	…	$F_{2,a+b+c+d}$

The distinction between enriched and non-enriched GO items and this particular ordering of items has been added for further discussion. It is not relevant in our approach, at its present form, where all annotated items are considered, and their contribution is evaluated through their annotation frequencies.

In this alternative approach, the frequencies are changed by zeros and ones. In the “Enriched in both lists” columns, both rows are filled by ones, in the “Enriched only in list…” columns, the corresponding row is filled by ones and the other row by zeros, and in the “Non-enriched…” columns all values are zeros. A convenient summary of these data is a contingency table:

	**Enriched in list 2**	**Non-enriched in list 2**	**Totals**
**Enriched in list 1**	a	b	a + b
**Non-enriched in list 1**	c	d	c + d
**Totals**	a + c	b + d	a + b + c + d

The proposal was to perform a test of positive dependency between GO item annotations in both lists. More precisely, to take a decision in view of the outcome of a Fisher’s exact test, with an alternative hypothesis of positive dependency. Rejecting the null hypothesis would be taken as an evidence of equivalence between both lists (in terms of the biological information provided by the GO) because “there is a significant” positive dependency between the enriched GO items in both lists.

Unfortunately, even for existing but very small degrees of positive dependency, a conclusion of “statistical significance” would be taken always for all tests dealing with such contingency tables (Fisher’s test, chi-square test…) for sufficiently high sample sizes. Except if chance brings to a zero value in the main diagonal of the table, a = 0. In such a case, the Fisher’s test p-value is one.

"Statistically significant" should not be confused with "relevant up to a given degree" which is the approach adopted in an equivalence test, as the one proposed in our paper.

We illustrate these ideas with two sets of evidences:

The first one is based on the pathogenesis based transcript sets (PBTs) data related to kidney rejection after transplantation. In Table 1 below, for levels 3, 4 and 5 at the BP ontology, all Fisher tests according to the suggested idea are compared with the equivalence test presented in our paper. As we go deeper in the ontology, the number of GO items also grows –in other words, the sample size of the contingency table grows. This increase, however, is due mainly, in most cases, to the increase in double negative frequency ("d" cell), that is, those items where neither list 1 nor list 2 show an enrichment. This is a natural consequence of the way that enrichment is established.

Consequently, except for those still having a zero in the main diagonal, nearly all Fisher tests provide significant results without any distinction between high and low levels of dependency. This does not supply any interesting information on which gene lists should be considered equivalent and which should not, because all are considered equivalent. On the other hand, the equivalence test based on the squared Euclidean distance only declares as equivalent some pairs of lists (here with an equivalence threshold proportional to the number of annotated GO items), in a way compatible with their origin.

Go level	Fisher-p value	Equivalence-p Value	list1	list2	a	b	c	d
**3**	2,91144989250101E-10	0,705567	ENDAT	IRITD3	6	7	1	544
**4**	1,68130863548538E-26	0,014252	ENDAT	IRITD3	19	42	17	3812
**5**	1,25058000314978E-34	0	ENDAT	IRITD3	24	102	25	10866
**3**	3,91835905413807E-05	0,514164	ENDAT	IRITD5	3	10	1	544
**4**	5,79219972857665E-16	0,007176	ENDAT	IRITD5	12	49	14	3815
**5**	7,63685110743352E-28	0	ENDAT	IRITD5	20	106	25	10866
**3**	0,000801099668945	0	IRITD3	IRITD5	2	5	2	549
**4**	4,74656902195339E-13	0	IRITD3	IRITD5	9	27	17	3837
**5**	3,67022425134025E-17	0	IRITD3	IRITD5	11	38	34	10934
**3**	0,068396640769274	0,999794	ENDAT	KT1	1	12	2	543
**4**	1,20557226296662E-09	0,925061	ENDAT	KT1	9	52	25	3804
**5**	1,73542995086793E-09	0,12135	ENDAT	KT1	11	115	70	10821
**3**	1	0,921374	IRITD3	KT1	0	7	3	548
**4**	0,000235233250668	2E-06	IRITD3	KT1	4	32	30	3824
**5**	1	0	IRITD3	KT1	0	49	81	10887
**3**	1	0,959606	IRITD5	KT1	0	4	3	551
**4**	0,204662103114894	0,006359	IRITD5	KT1	1	25	33	3831
**5**	0,043116285137276	0	IRITD5	KT1	2	43	79	10893
**3**	1	0,999987	ENDAT	KT1.1	0	13	1	544
**4**	1	0,998166	ENDAT	KT1.1	0	61	17	3812
**5**	1	0,910698	ENDAT	KT1.1	0	126	43	10848
**3**	1	0,998937	IRITD3	KT1.1	0	7	1	550
**4**	0,146480161633295	0,634653	IRITD3	KT1.1	1	35	16	3838
**5**	1	5E-06	IRITD3	KT1.1	0	49	43	10925
**3**	1	0,999276	IRITD5	KT1.1	0	4	1	553
**4**	1	0,908175	IRITD5	KT1.1	0	26	17	3847
**5**	1	0,003045	IRITD5	KT1.1	0	45	43	10929
**3**	0,005376344086017	4E-05	KT1	KT1.1	1	2	0	555
**4**	4,58283962334172E-28	0	KT1	KT1.1	14	20	3	3853
**5**	4,75838592654191E-68	0	KT1	KT1.1	34	47	9	10927

Table 1 Comparison between Fisher and Equivalence p-values for all pairwise comparisons between kidney gene lists at levels 3, 4 and 5 of the BP ontology

We also illustrate these ideas with a simple numerical example: consider a contingency table such as:

	Enriched in list 2	Non-enriched in list 2	Totals
**Enriched in list 1**	1	3	4
**Non-enriched in list 1**	3	13	16
**Totals**	4	16	20

The probability estimates from this table suggest a positive but weak dependency between the enriched items in both lists. The observed estimate of the enrichment concordance probability is 1 / 20 = 0.05, while under a hypothesis of independency the estimated probability would be (4 / 20) × (4 / 20) = 0.04. This makes a positive difference of 0.01. As would be expected, for these low sample sizes the Fisher test does not reject the null hypothesis of independency, it

supplies a p-value of 0.624355. If we iterate the process of repeatedly doubling all the frequencies in the table, the estimated degree of positive dependency remains constant, but the p-values progressively decrease. When the original table is multiplied by 2^6 = 1,280, the p-value is 0.01719419. Obviously, the above example is just one among many similar illustrative possibilities.

MORE ON THE FISHER'S TEST PROPERTIES

Fisher's exact test does not allow distinguishing between equivalent and non-equivalent gene lists at levels 3 or deeper in the Gene Ontology. Comparing enriched and non-enriched categories for two gene lists at these levels yields 2x2 contingency tables with a large number of non-enriched categories.

Using a more standard notation than in the previous section we can write:

	Enriched in list 2	Non-enriched in list 2	
Enriched in list 1	n_{11}	n_{12}	$n_{1\cdot}$
Non-enriched in list 1	n_{21}	n_{22}	n_2
	$n_{\cdot 1}$	$n_{\cdot 2}$	n

being, n_{11} / n_{22} the number of common categories enriched / unenriched in both lists and n_{12} / n_{21} the number of categories enriched in a list only (n_{11} = a; n_{12} = b; n_{21} = c; n_{22} = d). To illustrate the effect of n_{22} in the p-value we have considered two experimental situations:

- Situation 1. (n_{11}=5, n_{12}=0, n_{21}=0), (n_{11}=5, n_{12}=1, n_{21}=0), (n_{11}=5, n_{12}=1, n_{21}=1), (n_{11}=5, n_{12}=5, n_{21}=5), (n_{11}=5, n_{12}=10, n_{21}=10) (n_{11}=5, n_{12}=20, n_{21}=20)
- Situation 2. (n_{11}=15, n_{12}=0, n_{21}=0), (n_{11}=15, n_{12}=0, n_{21}=1), (n_{11}=15, n_{12}=1, n_{21}=1), (n_{11}=15, n_{12}=5, n_{21}=5), (n_{11}=15, n_{12}=15, n_{21}=15), (n_{11}=15, n_{12}=30, n_{21}=30)

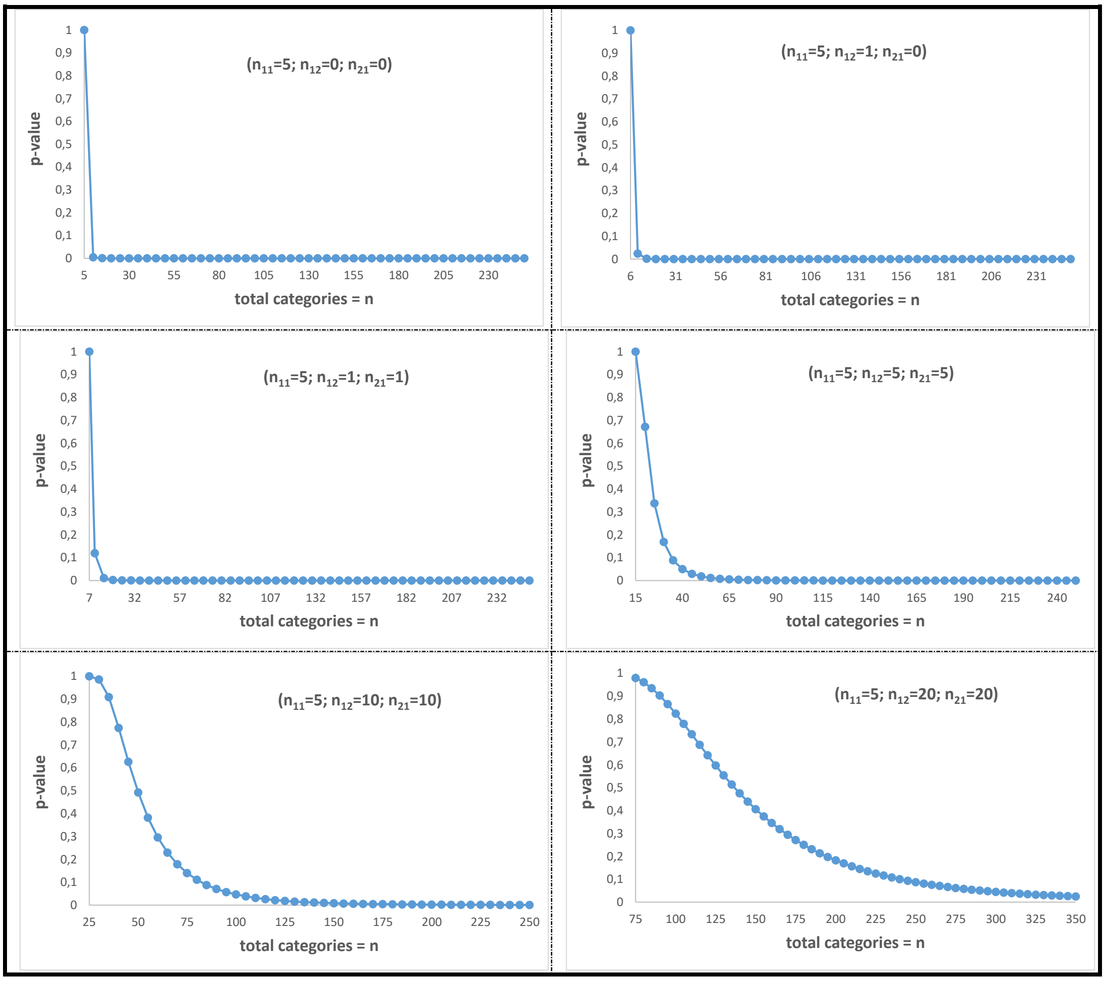

Figure 1. Experimental Situation 1: p-value varying the value of n_{22}

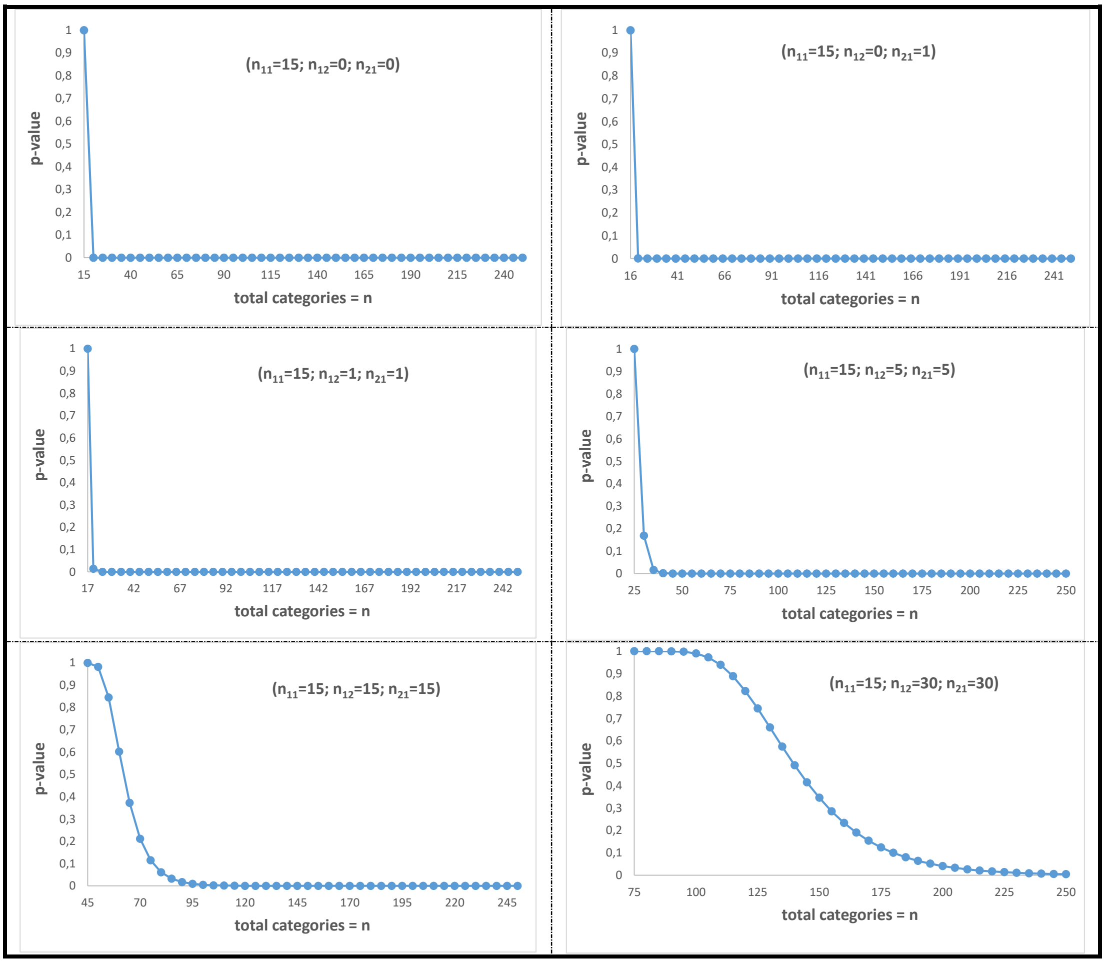

Figure 2. Experimental Scenario 2; p-value varying the value of n_{22}

The results obtained (Figures 1 and 2) allow to realize that the p-value tends to 0 when the total number of categories is high (level 3 and above in the GO). Specifically, rejection of null hypothesis in 2x2 contingency table has happened when n≥7, n≥9, n≥12, n≥40, n≥99 and n≥291 (experimental situation 1) and when n≥17, n≥18, n≥20, n≥33, n≥82 and n≥196 (experimental situation 2). Addition, the p-value is always 1 when $n_{22} = 0$.

Formally, it is possible to prove that the results obtained experimentally are also satisfied in general.

Holding constant marginal values in the contingency table,

$$p = \sum_{n_{11} \geq n_{11}(obs)} P[X = n_{11}] = \sum_{n_{11} \geq n_{11}(obs)} \frac{\binom{n_{1\cdot}}{n_{11}}\binom{n-n_{1\cdot}}{n_{\cdot 1}-n_{11}}}{\binom{n}{n_{\cdot 1}}}$$

is the p-value provided by the Fisher exact test when we test the hypotheses

$$\left.\begin{array}{l} H_0\colon p_{1/1} = p_{1/2} \\ H_0\colon p_{1/1} > p_{1/2} \end{array}\right\}$$

Under the null hypothesis, the variable X (X = number of categories enriched in both lists) follows a hypergeometric distribution H(n, $n_{\cdot 1}$, $n_{1\cdot}$). In this context, when n_{22} or $n_{11} = 0$ all possible contingency tables (n_{11}, n_{12}, n_{21} and n_{22} nonnegative and marginal values held constant) satisfy the condition $n_{11} \geq n_{11}$ (Obs) and, so, the p-value is always 1. Thus, when the overlap in enriched categories is 0 ($n_{11} = 0$) the null hypothesis (no equivalence) cannot be rejected. If the contingency table is reduced to categories enriched in at least one of the lists ($n_{22} = 0$ and non-

enriched categories in any of the two lists not being considered) the p-value will always be 1 (not equivalent). Furthermore, for high values of n (levels 3 and higher in GO) and $n_{1\cdot} < 0.1 \cdot n$ (which is a reasonable ratio in this field) it is possible to estimate the value of p considering the normal approximation of the hypergeometric. Specifically,

$$X \approx H(n, n_{\cdot 1}, n_{1\cdot}) \approx B(n_{\cdot 1}, \frac{n_{1\cdot}}{n}) \approx N\left(\frac{n_{\cdot 1} \cdot n_{1\cdot}}{n}, \sqrt{n_{\cdot 1} \cdot \frac{n_{1\cdot}}{n} \cdot (1 - \frac{n_{1\cdot}}{n})}\right)$$

One has that

$$p = P[X \geq n_{11}(obs)] = P\left[Z_{N(0,1)} \geq \frac{n_{11}(obs) - \frac{n_{\cdot 1} \cdot n_{1\cdot}}{n}}{\sqrt{n_{\cdot 1} \cdot \frac{n_{1\cdot}}{n} \cdot \left(1 - \frac{n_{1\cdot}}{n}\right)}}\right] \xrightarrow{n \to \infty} P\left[Z_{N(0,1)} \geq \infty\right] = 1$$

since $f(n) = \frac{n_{11}(obs) - \frac{n_{\cdot 1} \cdot n_{1\cdot}}{n}}{\sqrt{n_{\cdot 1} \cdot \frac{n_{1\cdot}}{n} \cdot (1 - \frac{n_{1\cdot}}{n})}}$ is an increasing function when n is large enough
