# Supplementary material for: An equivalence approach to the integrative analysis of feature lists
Source: BMC Bioinformatics. 2019 Aug 27;20:441. doi: 10.1186/s12859-019-3008-x (PMC6712676; doi:10.1186/s12859-019-3008-x)
Supplement: Supplementary file 7 — Summary results from a small simulation study performed to provide information about execution times in a realistic scenario. (PDF 166 kb) [file 12859_2019_3008_MOESM7_ESM.pdf]

# Supplementary simulation study. Analysis of execution times for 5 replicates

*Alex Sanchez, Jordi Ocaña and Miquel Salicrú*

## Computational efficiency and execution times

An important issue to be accounted for is computational efficiency. It depends on many factors like the computer where the programs are run, the number of lists to compare, the size (number of genes) of these lists and the number of GO nodes in the profiles being compared.

A small simulation study has been performed to provide information of execution times in a realistic scenario: Using a basic bioinformatic station (i7-4790 processor with 8GB of RAM running R 3.4 on 64 bits Windows 7 Enterprise) the process of determining the equivalence of a certain number of *random* gene lists has been executed repeteadly. This was done using the `equivClust` function of the current version (1.44.0) of package `goProfiles`. Times were measured by means of the R package `microbenchmark` which provides summary statistics for the running time along the replicates. The number of lists to be compared was **5, 10, 25 and 50**. For simplicity all lists had the same number of genes and this number was set at _ random gene lists of sizes **100, 200, 1000, 2000 and 5000**. The comparison was made at levels 2 gene and 3 of the "Biological Process" (BP) ontology.

Table 1 shows the summaries of the execution times in 5 replicates (if the number of genes was 5000 only one replicate was used). Notice that at level 3, the required time to build an equivalence dendrogram for 50 gene lists and 5000 genes is more than 32 hours (115858.16/3600=32.1828222) which is clearly not assumable for "ordinary calculations".

Table 1: Table 1, Summaries of the execution times in 5 replicates

| GOlevel | numLists | numGenes | min | lq | mean | median | uq | max | neval |
|---|---|---|---|---|---|---|---|---|---|
| 2 | 5 | 100 | 21.78 | 22.54 | 22.46 | 22.60 | 22.61 | 22.75 | 5 |
| 2 | 5 | 200 | 39.94 | 39.95 | 40.49 | 40.60 | 40.79 | 41.18 | 5 |
| 2 | 5 | 1000 | 190.93 | 193.90 | 196.88 | 197.42 | 200.28 | 201.85 | 5 |
| 2 | 5 | 2000 | 402.09 | 403.05 | 405.43 | 406.30 | 406.49 | 409.25 | 5 |
| 2 | 5 | 5000 | 1162.33 | 1162.33 | 1162.33 | 1162.33 | 1162.33 | 1162.33 | 1 |
| 2 | 10 | 100 | 64.60 | 64.86 | 65.09 | 65.10 | 65.36 | 65.53 | 5 |
| 2 | 10 | 200 | 120.94 | 121.94 | 122.83 | 122.04 | 123.43 | 125.78 | 5 |
| 2 | 10 | 1000 | 580.55 | 581.73 | 586.29 | 582.88 | 586.43 | 599.88 | 5 |
| 2 | 10 | 2000 | 1191.84 | 1193.99 | 1198.12 | 1194.65 | 1204.39 | 1205.71 | 5 |
| 2 | 10 | 5000 | 3569.97 | 3569.97 | 3569.97 | 3569.97 | 3569.97 | 3569.97 | 1 |
| 2 | 25 | 100 | 334.57 | 337.67 | 340.24 | 337.71 | 343.40 | 347.86 | 5 |
| 2 | 25 | 200 | 585.72 | 589.02 | 590.32 | 592.04 | 592.08 | 592.72 | 5 |
| 2 | 25 | 1000 | 2823.50 | 2830.39 | 2856.94 | 2861.41 | 2882.41 | 2887.00 | 5 |
| 2 | 25 | 2000 | 5861.17 | 5921.54 | 5930.98 | 5933.15 | 5962.99 | 5976.05 | 5 |
| 2 | 25 | 5000 | 18638.63 | 18638.63 | 18638.63 | 18638.63 | 18638.63 | 18638.63 | 1 |
| 2 | 50 | 100 | 1179.01 | 1203.87 | 1225.43 | 1242.87 | 1244.95 | 1256.47 | 5 |
| 2 | 50 | 200 | 2269.67 | 2271.40 | 2331.32 | 2285.38 | 2387.36 | 2442.80 | 5 |
| 2 | 50 | 1000 | 10236.89 | 10253.71 | 10605.71 | 10515.83 | 10882.41 | 11139.72 | 5 |
| 2 | 50 | 2000 | 21524.27 | 22231.70 | 22514.92 | 22541.41 | 22612.63 | 23664.61 | 5 |
| 2 | 50 | 5000 | 68553.81 | 68553.81 | 68553.81 | 68553.81 | 68553.81 | 68553.81 | 1 |
| 3 | 5 | 100 | 24.27 | 24.27 | 24.27 | 24.27 | 24.27 | 24.27 | 1 |
| 3 | 5 | 200 | 55.17 | 55.17 | 55.17 | 55.17 | 55.17 | 55.17 | 1 |

| GOlevel | numLists | numGenes | min | lq | mean | median | uq | max | neval |
|---|---|---|---|---|---|---|---|---|---|
| 3 | 5 | 1000 | 253.43 | 253.43 | 253.43 | 253.43 | 253.43 | 253.43 | 1 |
| 3 | 5 | 2000 | 542.17 | 542.17 | 542.17 | 542.17 | 542.17 | 542.17 | 1 |
| 3 | 5 | 5000 | 1430.19 | 1430.19 | 1430.19 | 1430.19 | 1430.19 | 1430.19 | 1 |
| 3 | 10 | 100 | 91.58 | 91.58 | 91.58 | 91.58 | 91.58 | 91.58 | 1 |
| 3 | 10 | 200 | 169.92 | 169.92 | 169.92 | 169.92 | 169.92 | 169.92 | 1 |
| 3 | 10 | 1000 | 929.68 | 929.68 | 929.68 | 929.68 | 929.68 | 929.68 | 1 |
| 3 | 10 | 2000 | 1923.07 | 1923.07 | 1923.07 | 1923.07 | 1923.07 | 1923.07 | 1 |
| 3 | 10 | 5000 | 5237.34 | 5237.34 | 5237.34 | 5237.34 | 5237.34 | 5237.34 | 1 |
| 3 | 25 | 100 | 509.27 | 509.27 | 509.27 | 509.27 | 509.27 | 509.27 | 1 |
| 3 | 25 | 200 | 983.91 | 983.91 | 983.91 | 983.91 | 983.91 | 983.91 | 1 |
| 3 | 25 | 1000 | 4904.14 | 4904.14 | 4904.14 | 4904.14 | 4904.14 | 4904.14 | 1 |
| 3 | 25 | 2000 | 10889.39 | 10889.39 | 10889.39 | 10889.39 | 10889.39 | 10889.39 | 1 |
| 3 | 25 | 5000 | 29216.93 | 29216.93 | 29216.93 | 29216.93 | 29216.93 | 29216.93 | 1 |
| 3 | 50 | 100 | 1887.05 | 1887.05 | 1887.05 | 1887.05 | 1887.05 | 1887.05 | 1 |
| 3 | 50 | 200 | 3583.98 | 3583.98 | 3583.98 | 3583.98 | 3583.98 | 3583.98 | 1 |
| 3 | 50 | 1000 | 18767.41 | 18767.41 | 18767.41 | 18767.41 | 18767.41 | 18767.41 | 1 |
| 3 | 50 | 2000 | 40803.71 | 40803.71 | 40803.71 | 40803.71 | 40803.71 | 40803.71 | 1 |
| 3 | 50 | 5000 | 115858.16 | 115858.16 | 115858.16 | 115858.16 | 115858.16 | 115858.16 | 1 |

Figure 1 shows the median execution times in minutes for different numbers of lists and different numbers of genes at GO levels 2 and 3.

```
## pdf
##   2
```

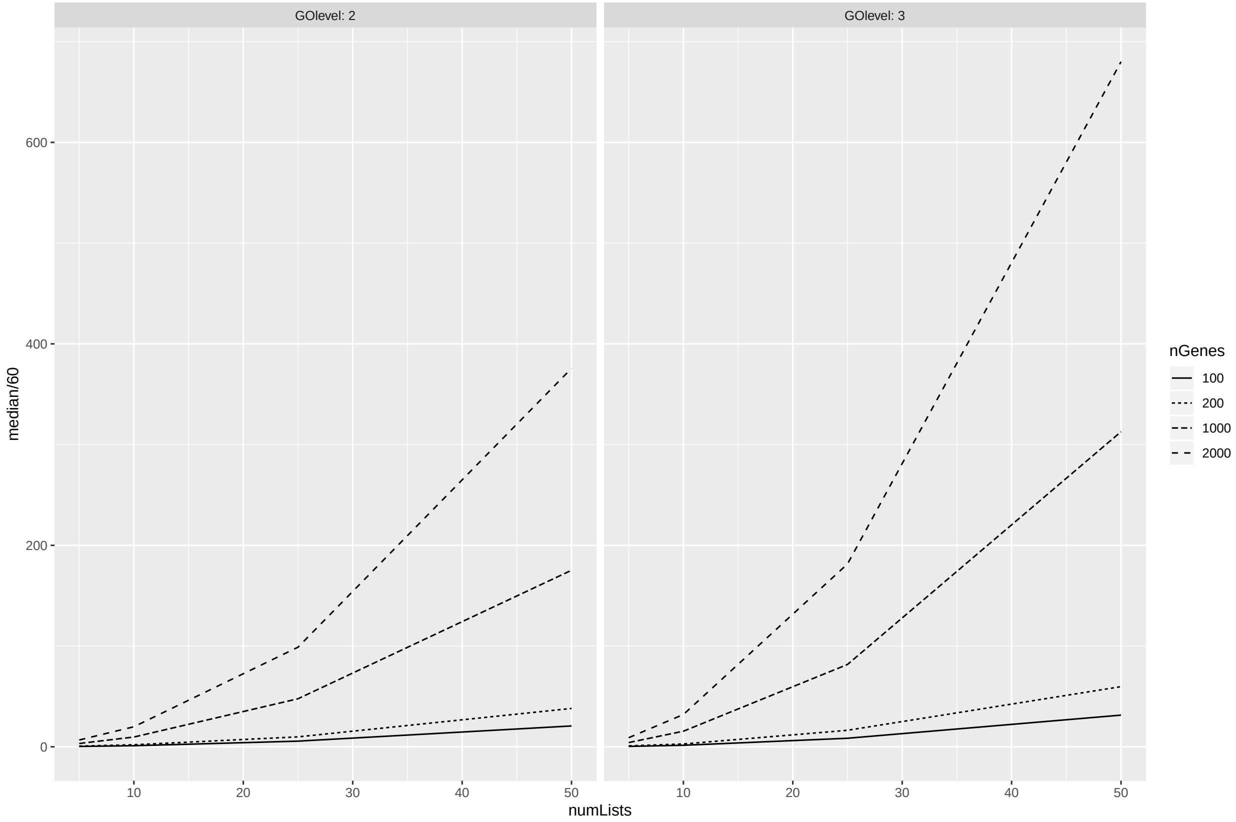


Figure 1: Figure 1. Median execution times in minutes for different numbers of lists and different numbers of genes at GO levels 2 and 3
